# The political polarization of health outcomes in the USA

**DOI:** 10.1038/s41562-026-02474-9

**Published:** 2026-05-14

**Authors:** Elizabeth Elder, Neil A. O’Brian

**Affiliations:** 1 Hoover Institution, Stanford University, Stanford, CA USA; 2 Department of Political Science, University of North Carolina at Chapel Hill, Chapel Hill, NC USA

**Keywords:** Politics and international relations, Human behaviour

## Abstract

Public health disparities provide an important lens for understanding social and political change in the USA. Using individual-level medical data and death records, this study shows that conservative Americans experienced worsening health and higher mortality than liberals during the 2010s. Here we find evidence consistent with two potential mechanisms. First, demographic realignment within political coalitions brought less healthy individuals into the conservative camp. Yet by the 2020s, demographic change, public policy and COVID-19 do not fully account for the widening gap in mortality rates. Public opinion data are consistent with a second mechanism: declining trust in medical professionals among right-leaning individuals, including lower willingness to seek care, follow clinical advice or believe in medication effectiveness, even for issues unrelated to COVID-19. These patterns suggest that growing ideological divides in health behaviours are leaving conservative Americans increasingly vulnerable to preventable health risks.

## Abstract (web summary)

Using individual-level medical data and death records, this study finds that conservatives in the USA experienced worse health and higher mortality than liberals during the 2010s. No significant gaps in biomarkers or mortality were present before the 2010s.

## Main

A prominent literature in public health investigates ‘social determinants of health’—non-medical factors that shape health outcomes—such as race, ethnicity, income, education, public policy and environmental factors. This article argues that political beliefs, by shaping trust, engagement and adherence to medical advice, have become an important social determinant of health, even beyond coronavirus disease 2019 (COVID-19)-related matters. Liberals and conservatives differ more today in their health outcomes than in decades past, and these differences are poised to persist as attitudes towards seeking and trusting care—factors that affect health outcomes—now divide the political left and right.

While previous work has investigated diverging health outcomes along political lines, much of this existing research relies on ecological data or self-reported health outcomes. These two approaches have led to different conclusions: ecological data that (for example) correlate county-level mortality rates and presidential vote show that people who live in ‘redder’ (that is, more Republican) counties have higher mortality rates^1–4^, perhaps due to differences in health policies between red and blue (that is, Democratic) places^5–9^. Conversely, a different set of studies rely on self-reported health outcomes to show that conservative people, for various psychological or political reasons, self-report better health^10–12^. Issues of aggregation or the misperception of one’s own health may well explain these contradictory findings, but their inconsistent directions prevent conclusions about the relationship between individuals’ politics and their health.

So far, the clearest evidence on the relationship between health and political beliefs at the individual level comes from studies linking death records to survey results. These studies^13–15^, conducted using data from the end of the twentieth century and the first decade of the 2000s, find modest evidence that conservatives have higher all-cause mortality rates than liberals, but that Republicans have lower mortality rates than Democrats. However, these data leave important questions unanswered: they do not capture causes of death (for example, car crashes versus disease-based deaths), health during the lifetime or changes in health over time, making it difficult to understand the mechanisms behind political differences. What is more, the health and political landscapes have drastically changed in the last 15 years as political coalitions have shifted and health has become increasingly politicized. New evidence on the relationship between politics and health is urgently needed.

This article explores three questions. First, we seek to reconcile the existing mixed findings on the relationship between politics and health using longitudinal, individual-level data. Second, we ask how this relationship has changed since 2010, when previous mortality analyses conclude. Third, we seek potential explanations for these changes, identifying promising candidates for future research.

To understand the relationship between politics and health in recent decades, we draw on individual-level medical data and death records from the National Longitudinal Study of Adolescent to Adult Health (referred to as the Add Health survey), which has tracked a nationally representative cohort of people who were adolescents in the 1990s (most born between 1976 and 1982) over the course of their lifetimes^16^. Unusually among health studies, Add Health includes a measure of political beliefs: self-reported liberal–conservative placement. It thus captures individual political orientation and medically validated health measures (for example, haemoglobin A1c (HbA1c) levels) and their changes over time, and individual-level cause-of-death data that extend into the COVID-19 era. Although Add Health’s only political measure is ideological self-placement (there is no usable measure of partisanship or vote choice), ideology is increasingly correlated with partisanship in recent years^17^.

We find that conservative Americans in this cohort, who were about as healthy as liberals in the early 2010s, experienced worsening health through the 2010s and higher mortality in the early 2020s. Roughly half of this new health gap is due to people changing their ideology over time, with new entrants to the conservative coalition being less healthy than new liberals. But another sizeable share is due to people who were already liberal or conservative diverging more in health over time. Changes in the socio-economics of the liberal and conservative coalitions—including education, income and insurance status—contribute to both processes.

By the 2020s, conservatives were dying at significantly higher rates than liberals, with the gap concentrated in internal causes (for example, heart disease, cancer and stroke). The divide since 2020 is substantial: while only 0.2% of ‘very liberal’ respondents died of internal causes between 2020 and 2022, the probability for people who identified as ‘very conservative’ was 1.14 percentage points higher (*P* = 0.021; 95% confidence interval (CI), (0.18, 2.11)). This gap is not limited to deaths from COVID-19 and is not reducible to demographic or geographic differences between the groups, nor is it a pure function of ageing: previous cohorts’ death patterns in older data did not show a similar correlation between health and ideology before 2010.

We suggest that these growing health gaps are consistent with a mechanism of politically rooted changes in engagement with the health system. Using a large public opinion survey, we find that people on the right, particularly Trump voters and Republicans, express less trust in their personal doctor and are less willing to seek care for non-COVID-19-related health problems^18,19^. We also find that people on the right with chronic illnesses are more sceptical than people on the left that medicines to treat those illnesses are safe and effective. This political divide in consumption of care may sustain or deepen the health divide that has emerged in recent decades. However, both these findings and those on health outcomes are purely descriptive; more work is needed to uncover causal relationships.

Our findings raise serious concerns about the equity of health outcomes between Americans of different political backgrounds: conservatives are becoming a less healthy population, and their growing disinclination towards seeking and following medical advice means that these differences may be difficult to address. Although many institutions have lost the trust of Americans in recent decades, the case of medicine is a particularly stark illustration of the consequences that can follow when politics leads people to divest from institutions that promote their welfare.

## Results

### Health data

To explore the relationship between politics and health, we draw on the Add Health survey. Add Health is an ongoing survey tracking a nationally representative cohort in the USA, first interviewed while in grades 7–12 in 1994. So far, this cohort has been interviewed five times.

Add Health interviews include survey questions on demographic and health attributes and measurements of ‘biomarkers’ collected in survey takers’ homes by trained interviewers. Five biomarkers are measured in both waves 4 and 5, which are our focus here: body mass index, lipids (that is, cholesterol), HbA1c levels, blood pressure and C-reactive protein levels. Add Health has also checked respondents’ vital status against the National Death Index up to 2022 to determine whether respondents have died. Because of their age, mortality is a rare, although important, outcome in this sample: those interviewed in wave 5 were born between 1974 and 1983, with over 92% born between 1977 and 1982. By 2021–2022, the cohort was largely between 40 and 45 years old.

In the third (2001–2002), fourth (2008–2009) and fifth (2016–2018) survey waves, Add Health also asked respondents whether they identified as very liberal, liberal, moderate, conservative or very conservative. Although Add Health does not have a usable measure of party identification or vote choice, it is worth emphasizing that its inclusion of any questions about political orientation makes it unusual among health surveys.

Due to Add Health’s school-based sampling design, standard errors are clustered at the school level. All data are weighted to be representative of the cohort in the given wave.

### Ideology and health markers during the lifetime

**Fig. 1:**
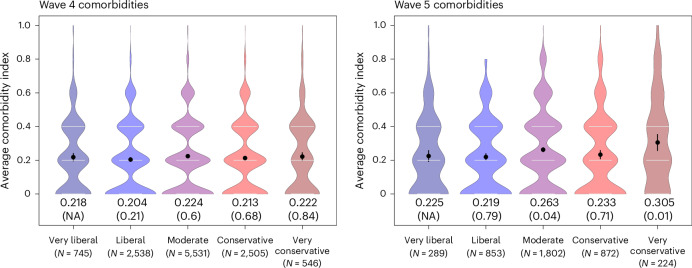
Biomarkers by ideological self-identification in waves 4 and wave 5. Health status by ideological self-identification in wave 4 (2008–2009) and wave 5 (2016–2018) of the Add Health survey. Both ideology and health are measured in wave 4 and wave 5; each represents contemporaneous health and ideological measures. Black points represent the average and black vertical lines are the 95% CI. The thin horizontal grey lines represent quartiles. Density is underlaid. The comorbidity index is a combined measure of the five health indicators for respondents with each measure. *P* values, in parentheses, are the difference between the category and those identified as very liberal using OLS regression, with two-sided test for significance. Full statistical reporting is provided in Supplementary Section 1.2. *P* value is not applicable (NA) for very liberal respondents.

Figure 1 shows the average number of comorbidities (out of five measured biomarkers; see Methods for details) for liberals and conservatives, as measured in waves 4 and 5. Higher numbers represent more total comorbidities and thus poorer health (Supplementary Section 1 presents results by individual measure). In 2008–2009 (wave 4), no discernible relationship exists between biomarkers (taken in 2008–2009) and ideological identification (as expressed in the 2008–2009 survey). An equivalence test presented in Supplementary Table 4 suggests that there is no difference greater than 0.04 between the most liberal group and any other.

However, by 2016–2018, the most conservative respondents (as measured in 2016–2018) were the least healthy (as measured in 2016–2018) (mean (*M*) = 0.305; 95% CI, (0.26, 0.36)). Table 1 shows the differences in the wave 4 and wave 5 cross-sections. The gap in health between the most liberal and conservative respondents in wave 5 represents one-third of the comorbidity scale’s standard deviation, making it statistically and substantively significant (0.08, 95% CI; (0.02, 0.14); *P* = 0.014). Supplementary Section 1.2 shows full tabular regression results.

**Table 1:** Differences in biomarkers by ideological self-identification between wave 5 and wave 4

Ideology	Wave 5 − wave 4, difference (s.e.) [95% CI]	*P* value
Very liberal	0.01 (0.02) [−0.03, 0.05]	0.740
Liberal	0.02 (0.01) [−0.01, 0.04]	0.139
Moderate	0.04 (0.01) [0.02, 0.05]	<0.001
Conservative	0.02 (0.01) [−0.01, 0.05]	0.144
Very conservative	0.08 (0.02) [0.03, 0.13]	0.001

The table represents the difference between wave 5 and wave 4 cross-section comorbidities using the index discussed and shown in Fig. 1. The 95% CI was constructed using a two-sided *t*-test.

What explains conservatives becoming less healthy than liberals between 2008 and 2016? The panel structure of the Add Health data allows us to separate two competing explanations: within-individual changes in health, where people who were already conservative in 2008 declined in health by 2016, or changes in ideology, where people who were less healthy in 2008 became more conservative by 2016.

**Fig. 2:**
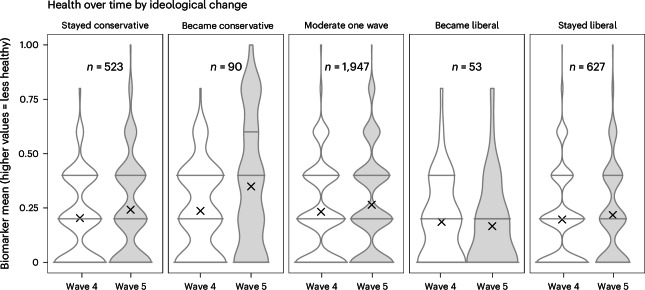
Health over time by pattern of ideological change. Health in waves 4 and 5 among people who were liberal or conservative in wave 4 or 5, divided by their pattern of ideological change or stasis between waves (listed across the top of each plot). ‘Stayed conservative (liberal)’ respondents were conservative or very conservative (liberal) in both waves. ‘Became conservative (liberal)’ respondents were liberal or very liberal (conservative) in wave 4 and conservative or very conservative (liberal) in wave 5. ‘Moderate one wave’ respondents were moderates in either wave 4 or 5 and liberal or conservative in the other wave. Health is measured by a comorbidity index, a combined measure of the five health indicators for respondents with each measure, so higher levels indicate worse health. Means for each group wave are marked with an X; density is underlaid, with 25th, 50th and 75th percentiles marked with grey lines.

Figure 2 shows health in waves 4 and 5 among respondents who maintained and changed their ideological beliefs over time. Each plot shows health among the same group of respondents in both waves. For simplicity and power, we group together very liberal and liberal (and very conservative and conservative) respondents; Supplementary Fig. 2 suggests this decision to group categories is not consequential for our substantive conclusions. Nearly every group became less healthy (that is, a higher comorbidity score) over time, as respondents aged between 2008 and 2016. But this change was not equal across groups: respondents who identified as liberals in wave 4 but ‘became conservative’ by wave 5 (Fig. 2, second plot) were slightly less healthy than other liberals in wave 4, but they became much less healthy than liberals (and indeed other conservatives) by wave 5. Meanwhile, people who became liberal between waves were healthier in wave 4 and became, if anything, more healthy by wave 5. This suggests that small differences in health in wave 4 predicted changes in ideology—and magnified differences in health—by wave 5.

Supplementary Table 5 presents a similar analysis using a cross-lagged dependent variable, with wave 4 health predicting wave 5 ideology and wave 4 ideology predicting wave 5 health. Those who are less healthy in wave 4 became significantly more conservative between waves 4 and 5, although the relationship loses statistical significance once controlling for demographics. Wave 4 ideology does not predict changes in health by wave 5.

**Fig. 3:**
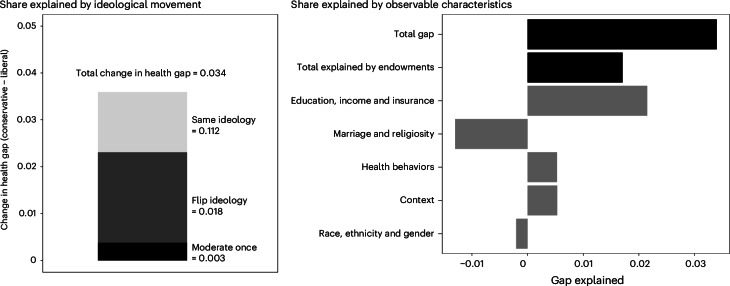
Decomposition of the change in the health gap over time. Left: Decomposition of the change in the gap in biomarkers between liberal and conservative respondents between wave 4 and wave 5 into the share of the gap attributable to three groups: ‘Same ideology’ (liberal or conservative in both waves), ‘flip ideology’ (liberal in one wave and conservative in the next, or vice versa) and ‘moderate once’ (liberal or conservative in one wave and moderate in the other). Right: the gap attributable to changes in the liberal and conservative groups’ ‘endowments’ of observable characteristics, followed by the gaps attributable to five categories of observable characteristics.

To understand the contribution of each group to the overall growth of the ideological gap, the left-hand plot in Fig. 3 decomposes the change along different dimensions. The overall gap in health between liberals and conservatives grew by 0.034 between waves. The left-hand plot in Fig. 3 shows that half of this change can be attributed to respondents who ‘flipped’ between liberal and conservative between waves (dark grey), while one-third is explained by subtler changes among the larger group of respondents who remained liberal or conservative in both waves (light grey). Only a small share is due to the respondents who shifted between moderate and either ideological fold (black).

So why might less healthy people become more conservative in this era—and already conservative people become less healthy? To explore this, the right-hand plot in Fig. 3 next presents the results of a Kitagawa–Oaxaca–Blinder decomposition^20^, a method that describes what proportion of the change in the health gap between liberals and conservatives over time can be attributed to changes in their observable characteristics, including demographics, health behaviours, contextual factors and socio-economic status.

Half of the increase in the health gap between liberals and conservatives over time (0.017 of 0.034) can be attributed to changes in these observable characteristics of the groups. A share of this is due to liberals’ relative gains in socio-economic status, including education, income and access to health insurance, all of which are associated with better health. For example, liberals went from being slightly less likely to have health insurance in wave 4 to slightly more likely in wave 5. This aligns with recent work showing more educated—and thus typically healthier^21^—Americans have become more liberal in recent years^22^.

Liberals were also helped, on balance, by relative gains in their lifestyle factors (including more exercise and less consumption of sugary drinks) and living in healthier places. Conversely, the gap between liberals and conservatives would have grown more if not for conservatives’ relative gains on two health-promoting dimensions: marriage and religiosity. Race, ethnicity and gender play small roles.

The remaining half of the increase in the health gap is not explainable by these observable factors. It could be due to the gaps in measured characteristics such as education or health behaviours becoming more impactful on health over time. Or, in a more intrinsically political sense, it could be due to unmeasured differences between liberals and conservatives—such as their values, beliefs or relationships to medicine—drifting further apart.

### Mortality over time

We next turn from measures of poor health during the lifetime to a downstream consequence: mortality. In addition to measuring biomarkers in wave 4 and wave 5, Add Health tracks each respondent’s vital status against the National Death Index, an index of deaths and their underlying cause administered by the US Centers for Disease Control and Prevention (CDC).

**Fig. 4:**
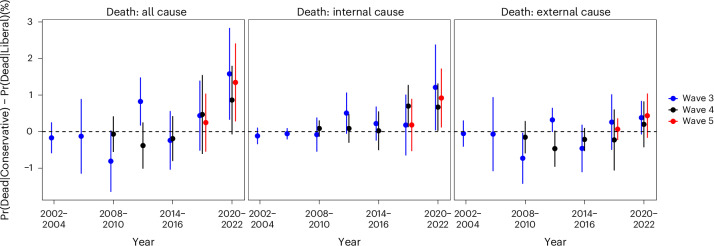
Mortality rate by ideological self-identification. Each point is the difference in the predicted probability (Pr) of death in a given 3-year period between the most liberal and most conservative respondents. A binary indicator of death (0–1) is regressed on a 5-point ideological scale (rescaled from 0 to 1) (using OLS; to display as a percentage point change, we multiply by 100). Higher values represent higher mortality rates for conservatives. Lines represent 95% CIs (2-sided *t*-test); standard errors are clustered at the school level (sampling unit). For example, the right-most points (at 2020) show the relationship between ideological as measured in waves 3–5 for deaths between 2020 and 2022. Ideological identification ranges from very liberal (0) to very conservative (1) as measured in wave 3, wave 4 and wave 5. Supplementary Section 5 has full statistical results. A total of 12,962, 13,892 and 11,477 respondents answered the question on ideology (and had a corresponding cross-sectional weight) in wave 3, wave 4 and wave 5, respectively.

Figure 4 shows the relationship between vital status in a given 3-year period, starting in the year listed along the *x* axis, and ideological self-identification as measured in a given wave. The left-hand plot shows deaths of all causes, the middle plot shows internal causes of death (sometimes called disease-based deaths, such as those from heart disease or cancer), and the right-most plot shows deaths from an external cause (for example, car accidents or drug overdoses). Using a linear probability model, we regress mortality on ideology measured on a 5-point scale from very liberal (coded as 0) to very conservative (coded as 1); the coefficient can be interpreted as the change in the probability of death going from the most liberal end of the political spectrum to the most conservative (presented in percentage points out of 100). The blue lines in Fig. 4 represent probability of death in the 3-year bin on the *x* axis by ideological self-identification as measured in wave 3 (2001–2002), black lines in wave 4 (2008–2009) and red lines in wave 5 (2016–2018).

We use 3-year bins to illustrate change over time while ensuring enough power given the relatively few deaths in any given year. As the timing of the survey waves did not overlap perfectly with 3-year bins, the 2008–2010 points for wave 4 and 2017–2019 points for wave 5 only reflect data from respondents who completed the survey by that year (wave 4 was conducted in 2008–2009 and wave 5 in 2016–2018). Supplementary Section 4 presents results by year and cumulatively, and the main results using a logit rather than ordinary least squares (OLS) model.

The negative coefficients in the first years of all-cause deaths show that liberals died at rates higher than or similar to conservatives in the early 2000s, although the coefficients do not statistically differ from zero. However, by 2020–2022, more conservative respondents (as measured in waves 3 and 5) were significantly more likely to die than more liberal ones (wave 3, coefficient (*β*) = 1.58, 95% CI, (0.32, 2.83), *P* = 0.014; wave 5, *β* = 1.35, 95% CI, (0.28, 2.41), *P* = 0.014).

Decomposing deaths between external and internal causes shows what is driving this shift. Conservatives began to trail their liberal peers in internally caused death in the late 2010s, although only wave 4 measures show statistical significance before 2020 (in 2017–2019, *β* = 0.7, 95% CI, (0.12, 1.28), *P* = 0.018). In the final time period (2020–2022), conservatives died more often of internal causes such as cancer, heart disease and diabetes—that is, causes related to attributes measured in the biomarker data, such as blood pressure and cholesterol. The linkage between ideology and mortality is consistently significant only after 2020, a point we will return to in the next section (wave 3, *β* = 1.21, 95% CI, (0.04, 2.38), *P* = 0.043; wave 4, *β* = 0.67, 95% CI, (0.02, 1.32), *P* = 0.04; wave 5, *β* = 0.92, 95% CI, (0.11, 1.73), *P* = 0.026). This pattern—the growth of death on the right—is weak or absent in externally caused deaths.

One explanation for this trend is that ideology generally becomes more linked to health outcomes as people age, meaning a similar gap would appear over time among similarly aged cohorts in previous years. Older data on politics and mortality provide evidence against a pure age effect: cohorts born in the decades before the Add Health group did not experience a growing link between their ideology and vital status over their 30s and 40s (Supplementary Section 3).

Another possibility is that ideology became more linked to health outcomes during the 2010s for reasons specific to this cohort of people, but not for older or younger cohorts. Without more recent data on a broader age group, we cannot say whether the years 2005–2022 represented a period of change across cohorts. The potential explanation we offer for these changes in the section ‘Politics and health after COVID-19’, however, is not specific to this cohort.

### Mortality in the COVID-19 era

Although ideology became more predictive of health during the 2010s, the relationship between politics and mortality became consistently significant only after 2020. This raises an important question: is the rise in conservatives’ mortality driven by, or even fully reducible to, the effects of COVID-19? Previous work^23^ has found that Republicans died of COVID-19 at higher rates than Democrats, especially after vaccine roll-out.

To test this, Table 2 investigates internally caused deaths in the final 3 years for which Add Health data are available: 2020, 2021 and 2022. Each model regresses mortality in 2020–2022 on political and demographic features of Add Health participants. Coefficients on each ideological subgroup represent the difference in mortality between people identifying as very liberal (the omitted category) and those in other groups, using a linear probability model. For reference, column 1 in Table 2 shows the predicted probability of internally caused death since 2020 as a function of ideological self-identification only. For these models, we use wave 5 ideology and demographics. Supplementary Section 7 presents results using various measures and weighting schemes.

**Table 2:** Predictors of death in 2020–2022

	(1) Internal deaths	(2) Internal deaths: non-COVID-19	(3) Internal deaths: COVID-19	(4) Internal deaths	(5) Internal deaths
Liberal	0.402 (0.214) [−0.02, 0.83]	0.377 (0.214) [−0.05, 0.80]	0.025 (0.025) [−0.02, 0.07]	0.571 (0.236) [0.10, 1.04]	0.492 (0.268) [−0.04, 1.02]
*P* value	0.063	0.080	0.318	0.017	0.069
Moderate	0.747 (0.191) [0.37, 1.12]	0.680 (0.182) [0.32, 1.04]	0.068 (0.039) [−0.01, 0.15]	0.648 (0.182) [0.29, 1.01]	0.507 (0.234) [0.04, 0.97]
*P* value	<0.001	<0.001	0.084	0.001	0.032
Conservative	0.745 (0.278) [0.19, 1.30]	0.468 (0.200) [0.07, 0.86]	0.279 (0.155) [−0.03, 0.59]	0.635 (0.296) [0.05, 1.22]	0.647 (0.372) [−0.09, 1.38]
*P* value	0.008	0.021	0.073	0.034	0.084
Very conservative	1.144 (0.489) [0.18, 2.11]	0.807 (0.423) [−0.03, 1.64]	0.343 (0.258) [−0.17, 0.85]	1.061 (0.529) [0.01, 2.11]	1.440 (0.685) [0.08, 2.80]
*P* value	0.021	0.059	0.186	0.047	0.037
Black				0.545 (0.555) [−0.55, 1.64]	0.873 (0.905) [−0.92, 2.66]
*P* value				0.327	0.337
Hispanic				−0.559 (0.253) [−1.06, −0.06]	−0.276 (0.393) [−1.05, 0.50]
*P* value				0.029	0.484
Other race				−0.497 (0.210) [−0.91, −0.08]	−0.693 (0.330) [−1.35, −0.04]
*P* value				0.019	0.038
Male				−0.232 (0.260) [−0.75, 0.28]	−0.261 (0.316) [−0.89, 0.36]
*P* value				0.374	0.410
Bachelor’s degree or more				−0.395 (0.206) [−0.80, 0.01]	−0.243 (0.301) [−0.84, 0.35]
*P* value				0.058	0.422
Income ≥ $100,000				−1.442 (0.367) [−2.17, −0.71]	−1.198 (0.521) [−2.23, −0.17]
*P* value				<0.001	0.023
Income between $30,000 and $100,000				−1.094 (0.390) [−1.86, −0.32]	−0.913 (0.507) [−1.92, 0.09]
*P* value				0.006	0.074
Health insurance				0.414 (0.415) [−0.41, 1.23]	0.204 (0.498) [−0.78, 1.19]
*P* value				0.321	0.683
Birth year				−0.139 (0.091) [−0.32, 0.04]	−0.143 (0.108) [−0.36, 0.07]
*P* value				0.127	0.188
Rural				−0.410 (0.342) [−1.09, 0.27]	−0.330 (0.715) [−1.75, 1.08]
*P* value				0.233	0.645
County health quartile				0.155 (0.134) [−0.11, 0.42]	
*P* value				0.249	
Constant	0.002 (0.002) [−0.00, 0.01]	0.002 (0.002) [−0.00, 0.01]	0.000 (0.000) [−0.00, 0.00]	276.593 (179.616) [−78.81, 631.99]	
*P* value	0.323	0.323	1.000	0.126	
*N*	11,183	11,171	11,138	10,278	10,278
Fixed effects	No	No	No	No	County

Values are given as estimate (s.e.) [95% CI], unless otherwise specified. Outcome variable is measure of death in 2020–2022 (1 if died, 0 if still alive); classification of death types are given in column titles. Coefficients represent percentage points from a linear probability model (OLS) with very liberal respondents as the reference group. Standard errors are clustered at the school level (sampling unit); *P* values computed using a two-sided *t*-test. Data are weighted to be representative of the population in wave 5. For example, the top-left cell can be interpreted as liberal respondents are 0.402 percentage points more likely to die than people who identify as very liberal. Ideology is measured by wave 5 ideological self-identification (and demographics) if available. Supplementary Section 7 presents various robustness checks and weighting schemes. See ‘Mortality analyses’ in the Methods for further details.

A strong relationship appears: participants who identify as very conservative were about 1.144 percentage points (95% CI, (0.18, 2.11); *P* = 0.021) more likely to die in 2020–2022 than those who were very liberal. Unlike in the biomarker analyses, moderate, conservative and very conservative respondents showed worse health—not only the most conservative.

Columns 2 and 3 in Table 2 draw on granular cause-of-death data to divide deaths from COVID-19 (column 3) from deaths from all other internal causes (column 2). Even once COVID-19 deaths are separated out, column 2 shows that liberals, moderates and conservatives were all more likely to die during the 2020–2022 period than the most liberal respondents.

One possibility is that COVID-19 deaths are misclassified as non-COVID-19 deaths. It is not possible to entirely rule out this explanation. However, the low COVID-19 mortality rate as reported by the CDC among this age group casts doubt on this explanation. We also checked second- and third-order causes of death (rather than underlying cause of death) to see if COVID-19 was a secondary factor; it was not.

What else might explain conservatives’ higher death rates during this period? As in our analysis of biomarkers, we can test the extent to which the gap is explained by observable differences between liberals and conservatives, including their demographics, resources and geographic contexts. The remaining columns of Table 2 regress internally caused deaths on ideology and a range of covariates related to these explanations.

Column 4 in Table 2 shows that a range of individual-level characteristics fail to fully explain the gap between the most liberal and conservative respondents. While race, ethnicity and income predict mortality, the coefficients on ideological groups remain large and statistically significant.

Column 5 in Table 2 moves beyond individual characteristics to geographic context. Much of the previous work on the relationship between health and politics compares health outcomes between more-and-less conservative localities (for example, counties or states). Differences in mortality between liberals and conservatives in these analyses can be explained by differences in the public policies, social norms or access to healthcare in the places where they live. The large sample matched to death records allows us to introduce county fixed effects, which compare liberals and conservatives within the same counties. This strategy rules out many plausible place-based factors and more cleanly isolates variation between individuals.

As with individual-level characteristics, county-level factors do not fully explain the relationship between ideology and mortality: even conservatives and liberals living in the same county have different health outcomes. These data cast doubt on entirely policy-driven explanations for differences in health outcomes^8,24^.

### Politics and health after COVID-19

By 2020–2022, conservative Americans were less healthy and more likely to die than their liberal peers. Although demographics and place-based characteristics explain some of this gap, a substantial share remains unexplained.

We propose one explanation for this persistent divide: political beliefs increasingly shape consumption of healthcare and thus health outcomes. This notion has been readily applied to COVID-19-related healthcare (for example, vaccines) and outcomes (for example, infection and death rates). But what if those on the political right are less likely to visit, trust and follow the advice of their doctor on health matters entirely unrelated to COVID-19? Existing research shows that while confidence in medicine was once non-partisan, Republicans began to show less confidence than Democrats during the pandemic^25,26^. At the same time, as liberals have come to trust more in scientific experts, they may have become more compliant with medical advice^19^. Lower trust and adherence to medical advice correlate with worse health outcomes^27^, so a broader gap in health could follow.

To measure the relationship between politics and health attitudes and behaviours today, we fielded an original survey that asked respondents a series of questions assessing their willingness to engage with the medical system (*N* = 21,751). The survey was fielded on an online sample and weighted to approximate national demographics (see ‘Survey methodology’ in Methods for details). A key intervention is analysing not just trust in medicine as an institution, but trust in and behaviours towards individuals’ direct health providers. As it was fielded after the end of the Add Health data, the survey cannot directly test trust as an explanation for changing patterns in health outcomes. Instead, our goal here is to test whether a political gap in trust exists today to motivate future work testing its causal power over time (but see Supplementary Section 10 for suggestive survey evidence on the timing of this gap).

**Fig. 5:**
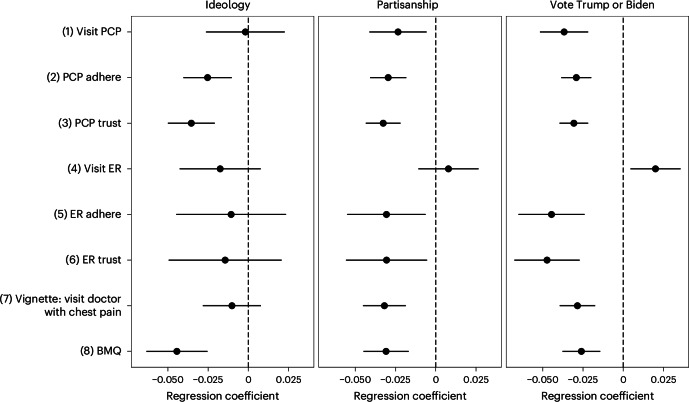
Engagement, trust and adherence to doctors and medicines. Points represent multivariate OLS regression coefficients of the political variable of interest labelled in the graphical header (covariates specified in Methods); lines represent 95% CIs. Each outcome variable is listed down the left-hand column; rescaled from 0 to 1, where higher values represent that the respondent is more likely to visit, trust and adhere to their PCP or ER physician. Measures of political preference are listed across the top of each model (higher values represent the rightward alternative; partisanship and ideology rescaled from 0 to 1). For example, the first row shows the relationship between visiting one’s PCP and ideology (left), partisanship (middle) and vote choice (right). A total of 16,541 respondents reported supporting either Trump or Biden, 21,694 reported a party identification and 21,722 reported an ideological identification. Full models, statistical reporting and alternative specifications in Supplementary Section 8.

Figure 5 shows the results of regressing measures of engagement with the medical system on respondents’ ideology, partisanship and vote choice (using OLS regression). The models in Fig. 5 include demographic controls and state fixed effects. Supplementary Section 8 presents results from bivariate models and among those respondents that report having chronic diseases (Methods for more details).

The left-hand plot in Fig. 5 shows the results of regressing measures of engagement with healthcare on self-reported ideology. The other two plots measure health engagement by political beliefs for two political indicators not available in the Add Health data: partisanship (Fig. 5, middle) and Trump–Biden vote choice (Fig. 5, right). We include partisanship and vote choice because they, like ideology, are central to understanding political divisions.

Lines 1–3 in Fig. 5 show the relationship between political beliefs and trust and engagement with one’s primary care provider (PCP). The left-hand plot in Fig. 5 shows no evidence that liberals are more likely than conservatives to have visited their PCP in the prior year (line 1, *β* = −0.002, 95% CI, (−0.03, 0.02), *P* = 0.88), but conservatives are significantly less likely to exhibit trust in their PCP (lines 2 and 3, *β* = −0.035, 95% CI, (−0.05, −0.02), *P* < 0.001) and follow the advice of their PCP (*β* = −0.025, 95% CI (−0.04, −0.01), *P* = 0.001).

The middle and right-hand plots in Fig. 5 show that Republicans and Trump voters are less likely to report having visited their PCP in the past year, and are less likely to trust and follow their advice (lines 1–3; for visit PCP, vote choice (*β* = −0.037, 95% CI, (−0.05, −0.02), *P* < 0.001) and partisanship (*β* = −0.023; 95% CI, (−0.04, −0.01), *P* = 0.01); for trusting PCP, vote choice (*β* = −0.031, 95% CI, (−0.04, −0.02), *P* < 0.001) and partisanship (*β* = −0.033, 95% CI, (−0.04, −0.02), *P* < 0.001); for following PCP advice, vote choice (*β* = −0.029, 95% CI, (−0.04, −0.02), *P* < 0.001) and partisanship (*β* = −0.03, 95% CI, (−0.04, −0.02), *P* < 0.001).

Lines 4–6 in Fig. 5 show the relationship between political beliefs and engagement and trust in an emergency room (ER) doctor, rather than a PCP. Ideology and partisanship are not significantly related to having visited the ER, but Trump voters are more likely to report having been to the ER (*β* = 0.02, 95% CI, (0.00, 0.04), *P* = 0.012).

Once at the ER, conservatives and liberals do not exhibit statistically different levels of trust and adherence. However, Republicans are less likely to trust (*β* = −0.031, 95% CI, (−0.06, −0.01), *P* = 0.017) and follow the advice of ER doctors than democrats (*β* = −0.031, 95%CI, (−0.06, −0.01), *P* = 0.014), and Trump voters are less likely to trust (*β* = −0.047, 95% CI, (−0.07, −0.03), *P* < 0.001) and follow advice than Biden voters (*β* = −0.045, 95% CI, (−0.07, −0.02), *P* < 0.001).

Line 7 in Fig. 5 examines whether politics predicted people’s willingness to seek care in the case of an emergency. We adopted a vignette from ref. ^28^, asking respondents how likely they were to go to their primary care doctor if they felt pain in their chest that was so severe that it made them sick, but it wore off in a few minutes. Trump voters and Republicans reported that they would be less likely to go to their primary care doctor if feeling this pain; there is no significant relationship with ideology (vote choice, *β* = −0.028, 95% CI, (−0.04, −0.02), *P* < 0.001; partisanship, *β* = −0.032, 95% CI, (−0.05, −0.02), *P* < 0.001; ideology, *β* = −0.01, 95% CI, (−0.03, 0.01), *P* = 0.27).

Finally, line 8 of Fig. 5, which presents an analysis of only people who report having a chronic illness, shows that vote choice, partisanship and ideology all predict whether a person with a chronic illness is confident in the efficacy of their medications to treat that chronic illness (for example, medicine to treat hypertension) using the Beliefs about Medicines Questionnaire (BMQ; adopted from ref. ^29^). Right-leaning respondents were more likely to question the usefulness of the medications they use to manage their disease by all 3 measures (vote choice, *β* = −0.026, 95% CI, (−0.04, −0.01), *P* < 0.001; partisanship, *β* = −0.031, 95% CI, (−0.04, −0.02), *P* < 0.001; ideology, *β* = −0.044, 95% CI, (−0.06, −0.03), *P* < 0.001).

Supplementary Section 9 explores the relationship between partisanship, ideology and vote choice, which have become increasingly correlated in recent years. It is unclear why the relationship is weaker by ideology than vote choice or partisanship. One explanation is that about 39% of the sample identifies as neither liberal nor conservative (calling themselves moderate)—a considerably higher rate than the number of people who say they do not align with either party. When looking at trust and engagement by vote choice just among ideological moderates, moderate-Trump and moderate-Biden voters have significantly different health behaviours.

Taken together, these results suggest that right-leaning Americans—as measured by their vote choice, partisanship and to a lesser extent their ideology—are less willing to visit, trust and adhere to the advice of their PCPs. This is true even among people with chronic conditions that require regular contact with the medical system for management and appears across a variety of outcomes unrelated to care for COVID-19.

## Discussion

We have presented evidence that, by a number of measures at the individual level, conservatives in middle adulthood steadily became less healthy than their liberal peers over the course of the 2010s. They also began dying at higher rates. The data we draw on here offer valuable insight into the relationship between measured health outcomes and political preferences at the individual level in the contemporary era.

During the COVID-19 pandemic, this relationship persists even after accounting for their demographics and local contexts. Using a large survey, we offer evidence consistent with a potential explanation for this growing gap: even after the pandemic and for matters beyond COVID-19, right-leaning Americans are less likely to seek medical care and follow medical advice. While these results describe a growing divide based on political views, more work is needed to determine whether this relationship is a causal one.

The Add Health data we draw on here is rich in its suite of measured biomarkers, but it contains only a single political measure: ideology. There is no usable question about partisanship or vote choice; in the wave 3 survey, respondents were asked about partisanship, but 65% of respondents skipped the question, making the measure unreliable. As ideology, partisanship and vote choice have become increasingly linked (gold-standard data on the 2020 election shows that 90% of self-identified liberals also called themselves Democrats), there is reason to expect similar patterns in health for the latter two measures, but further data are needed to test this expectation.

As our original survey took place in 2024, and Add Health does not collect measures on trust in medicine, we are also unable to evaluate whether the onset of the political gap in trust corresponds with the divergence of health outcomes. Future work should investigate whether the timing of partisan gaps in engagement with the healthcare system coincide with the emergence of gaps in health.

Taken together, these findings present a troubling picture for the polarization of health outcomes. The decline in conservatives’ health may not have been a cause for concern among policymakers if it largely reflected the reshuffling of less healthy people between political coalitions. But demographic differences cannot explain the full gap between left and right. Instead, the conservative coalition, which entered the pandemic with health metrics that made them more vulnerable to serious complications, is now more likely to eschew medical advice. If this pattern continues as the pandemic recedes—which our results suggest it will, as right-leaning Americans’ scepticism of medicine extends to other conditions—it will only become more difficult for public health officials and doctors to successfully reach conservative Americans. The gap between the left and right’s health outcomes is poised to grow.

## Methods

### Add Health sample details

Add Health is a longitudinal survey of a cohort of Americans who were adolescents in the 1990s. Respondents were initially selected from high schools, which were chosen through a random sample stratified by region, urbanicity, ethnicity and size, and paired middle schools.

This yielded 132 schools across the USA. Respondents to the initial survey (wave 1), which took place during the 1994–1995 school year, were selected from attendees of these schools with oversamples of populations of interest. Respondents to subsequent waves were selected from those who participated in the wave 1 survey, with selected additions and removals based on power needs for particular populations. We focus here on wave 3 (2001), wave 4 (2008–2009) and wave 5 (2016–2018). As respondents are clustered in schools and probability of selection varied between schools and pupils, the data are weighted, with weights provided by the study conductors, stratified by region and clustered at the school level. For more information, see https://cdr.lib.unc.edu/concern/articles/k0698990v?locale=en.

This research and our presentation of results complies with Add Health’s regulations for use. Our use of the Add Health data was reviewed and determined to be exempt by institutional review boards at the University of Oregon (STUDY00001107) and Stanford University (protocol 14166). This secondary analysis did not require additional consent from or compensation of respondents, and we played no role in determining the sample size for the study. However, respondents provided written informed consent for participation in all aspects of Add Health according to ethical guidelines: https://addhealth.cpc.unc.edu/documentation/frequently-asked-questions/. These analyses were not pre-registered.

### Biomarker analyses

Figure 1 draws on biomarker measures collected by trained interviewers in the homes of respondents in wave 4 and 5 of the survey: body mass index (based on measures of height and weight), blood pressure, and lipids (that is, cholesterol), HbA1c (that is, blood glucose) and C-reactive protein (that is, inflammation) levels based on a blood test. For the comorbidity index, we exclude respondents missing data for any of these biomarker measures. We transform each biomarker measure into a binary indicator for whether the respondent meets the conventional threshold for a concerning reading (for example, HbA1c levels indicative of diabetes) and we construct a comorbidity index by averaging these indicators.

The plot in Fig. 1 show the average comorbidity index in each ideological group—that is, the average number of concerning biomarker indicators in each group. The first plot shows wave 4 comorbidities by wave 4 ideology, weighted by wave 4 weights, while the second plot shows wave 5 comorbidities by wave 5 ideology, weighted by wave 5 weights. To obtain the *P* values shown in parentheses, we regressed the comorbidity index on ideological category, with very liberal as the omitted category; *P* values represent the significance of the difference from this category. This test and all other statistical tests we report are two-sided. The 95% CIs are indicated with bars. These regressions are also weighted according to the relevant wave.

Figure 2 shows means of this same comorbidity index. This analysis is limited to respondents who were not missing any comorbidity values in wave 4 or wave 5 nor ideology measures in wave 4 or wave 5. Respondents are split into groups based on whether they were liberal (conservative) in both waves (far right and left plots), whether they switched from being conservative to liberal (or vice versa) between waves (centre right and centre left plots), or whether they were moderate in one wave and liberal or conservative in the other (centre plot). People who were moderates in both waves are excluded as they cannot explain changes in health between liberals and conservatives. Wave 4 biomarkers’ bars use wave 4 weights, while wave 5 biomarkers’ bars use wave 5 weights.

Figure 3 decomposes the mean difference in the gap in comorbidity index between all liberals and all conservatives in wave 4 (wave 4 measures and weights) from the same difference in wave 5 (wave 5 measures and weights). For the left plot, we calculated this quantity separately for three groups simplified from the previous figure: those who kept their ideology, those who flipped between ideologies, and those who were moderate in one wave but not the other. We then multiplied these differences by the proportion of the sample that group represents. We plot these quantities as the change in gaps explained by each group. As this is a descriptive exercise, not an inferential one, we do not report standard errors for these estimates.

The right-hand plot of Fig. 3 subjects the overall change in the health gap to the ref. ^20^ extended Kitagawa–Oaxaca–Blinder decomposition method for panel data. The goal of this method is to understand changes over time in the size of a gap in some outcome between two groups. In particular, given some set of variables that could predict differences in the outcome, the method estimates what share of the change in gap is explained by changes in the groups’ levels of those predictors.

For example, in wave 4, 18.7% of liberals had high incomes, compared with 13% of conservatives, a 5.7 percentage point gap. Among both liberals and conservatives in wave 4, people with high incomes had fewer comorbidities. By wave 5, the gap had grown: 40.2% of liberals and 33.8% of conservatives had high incomes, a 6.4 percentage point gap. Holding all else constant, including the predictiveness of income for health, this larger gap in incomes would mean liberals’ health advantage would grow between waves 4 and 5. The second bar in the right-hand plot of Fig. 3 shows the total change in the gap that can be explained by changes in levels of all the covariates measured.

The third through seventh bars in the right-hand panel of Fig. 3 show the changes in the health gap that result from changes in the gap between liberals’ and conservatives’ levels of the following variables: education, income (in three categories) and insurance status (grouped together as socio-economic variables); marital status and religiosity (grouped together as indicators of traditionalism); possession of a sedentary job, consumption of fast food, consumption of sugary drinks, physical activity and smoking (grouped together as health behaviours); county of residence’s quartile rank on health-promoting factors and county of residence’s life expectancy (grouped together as measures of context); and race, ethnicity and gender. As in any regression model, it can be difficult to independently interpret the contributions of variables that are correlated with one another, so these sets of correlated variables are grouped together for presentation (with race, ethnicity and gender grouped for simplicity due to their small contributions).

When a wave 4 measure of one of these was missing, we substituted a measure from wave 3 when available. We then excluded all respondents missing any of these variables. Models of wave 4 outcomes used wave 4 weights, and models of wave 5 outcomes used wave 5 weights. As this is a descriptive exercise, not an inferential one, we do not report standard errors for these estimates.

Added together, changes in gaps in these observable factors explain about half of the change in the health gap between liberals and conservatives over time. As Fig. 3 shows, some of this change is due to ideological ‘stayers’ changing their attributes—for example, both-wave liberals were more likely to get college degrees between wave 4 and wave 5, increasing their education advantage over both-wave conservatives. Another share is due to ideological changers, as people with college degrees were more likely to become liberal between waves, increasing the education advantage of the liberal coalition further.

It is important to note that this is a purely descriptive analysis. We cannot claim, for instance, that increases in liberals’ incomes caused them to become differentially healthier; it is equally plausible that some shared factor we do not measure here, such as family background, explains both changes in income and changes in health. Instead, the goal is to understand what share of the change in gap could have been predicted based on the kinds of demographic, behavioural and contextual factors we already know are related to health—and what share must be explained in some other way.

The unexplained half of the change in the health gap is an important subject for future research. The decomposition method used here provides some promising hints. To return to the example of income, we saw that some of the increase in the health gap can be explained by liberals’ growing edge in income. But it is also the case that income became more predictive of health between waves 4 and 5, especially among conservatives: in wave 4, a high-income conservative respondent had a comorbidity index 0.008 lower on average, while by wave 5, it was 0.119 lower (on a scale from 0 to 1). The reasons for this growing protective power of income among conservatives merit further investigation.

### Mortality analyses

As described in the main text, Fig. 4 shows the results of linear probability models regressing death in the 3-year interval on the *x* axis on ideology measured in wave 3, wave 4 or wave 5. Models use weights in the wave in which ideology is measured to ensure the sample is nationally representative of the cohort at the time of interview. Add Health gathers mortality information for all respondents, so only those missing ideology in the relevant wave are excluded.

Table 2 presents similar models focusing only on deaths between 2020 and 2022, regressing death in this period on ideology measured in wave 5, with additional controls measured in wave 5 added in models 4 and 5, and county fixed effects (based on county of residence in wave 5) in model 5.

In addition to ideological self-identification, which is described in the main text, we include controls for the respondent’s self-reported race/ethnicity with self-identified as white non-Hispanic as the omitted category (white, Black Hispanic and other); sex assigned at birth (coded 1 = male; 0 = female); dummy variable for whether the respondent had reported receiving a college degree or more by time of interview (BA+); a dummy variable for self-reported household income (over $100,000 per year, between $30,000 and $100,000, and less than $30,000) with the last category as the omitted category; a dummy variable for whether the respondent reported having health insurance; the reported year of birth (birth year); 2017 health factor quartile (scaled 1–4) for the respondent’s county (includes health behaviours, social and economic factors and physical environment) as compiled by the Robert Wood Johnson Foundation (County Health quartile); and a dummy variable for whether the respondent’s census tract was classified as rural (less than 94 people per square kilometre is classified as rural, which equals 250 people per square mile, a common classification for rurality, see https://www.ncruralcenter.org/how-we-define-rural/).

### Survey methodology

To measure the post-COVID-19 relationship between politics and health attitudes and behaviours, we fielded an original survey of 21,751 people 18 years and older living in the USA through PureSpectrum, an online panel management platform. The data were collected by the Civic Health and Institutions Project, a 50 States Survey (CHIP50) in the spring of 2024 and the study was determined exempt by the CHIP50 home institution’s institutional review board at the University of Rochester (STUDY00009146).

Respondents were recruited from all 50 states and Washington, DC, with an over-sample of African American, Asian and Hispanic respondents. We use a post-stratification weight provided by the data vendor to ensure the sample represents the population of people living in the USA^30^. See Supplementary Section 11 for details on the sample’s demographics.

This analysis of anonymized data complies with relevant regulations. Consent was obtained from respondents, who were compensated by third-party survey providers. These analyses were not pre-registered. No statistical methods were used to pre-determine sample sizes, but this sample is larger than the Add Health samples used for the other analyses, which allowed detection of political differences in health.

Using this large sample, we measure a variety of health-related behaviours in the general public and among a subsample of people who report having one of five chronic illnesses common in the USA: high cholesterol, high blood pressure, cardiovascular disease, type 2 diabetes and chronic obstructive pulmonary disease. In this sample, 38% of respondents report having been diagnosed with at least 1 of these chronic conditions.

We asked respondents a series of questions that assessed their willingness to engage with the medical system. We first asked whether they had visited a PCP in the past year and, if so, how much they trusted that physician and adhered to their recommendations. We asked the same about ER physicians (although only 22% of people report using the ER compared with 78% reporting they had seen a PCP in the previous year). We followed this with a hypothetical scenario, asking respondents whether they would visit a doctor if they experienced chest pain.

Finally, we asked additional questions of the respondents who indicated they had any of five chronic health conditions (high blood pressure, high cholesterol, type 2 diabetes, heart disease or chronic obstructive pulmonary disease) and had been prescribed medications to treat that condition. If respondents indicated they had multiple chronic illnesses, we randomly selected one on which to enquire further. These questions are adopted from the BMQ^29^, which was designed to assess whether respondents believe medication prescribed for their chronic illness is safe and effective. The BMQ measures beliefs about a medication’s effectiveness. Due to survey space, we adopted four questions from the BMQ that asked, ‘my life would be impossible without my [fill in disease] medication’; ‘without medication for my [fill in disease], I would be very ill’; ‘I sometimes worry about the long-term effects of my [fill in disease] medication’; and ‘medication for my [fill in disease] disrupts my life’. This is important because if people report that they believe their medicines are safe and effective, they are more likely to take them^29,30^. Adherence to medication lowers mortality rates and improves health among those with chronic illness^27,31,32^.

Wording, choices and summary statistics for each of the dependent variables is described in detail:

1. Visit PCP: ‘In the last 12 months, have you seen any of the following doctors … a primary care provider?’ (*M* = 0.78, s.d. = 0.41)
2. PCP adhere: ‘How closely would you say you have followed the following healthcare professionals’ advice or treatment recommendations in the last 12 months, such as taking medications as instructed, returning for follow-up appointments, and so on? A primary care provider: 5 = extremely closely, 4 = very closely, 3 = somewhat closely, 2 = not very closely, 1 = not at all closely’ (rescaled 0–1; *M* = 0.75, s.d. = 0.23)
3. PCP trust: ‘For each of the following healthcare professionals that you have seen in the last 12 months, please indicate to what extent you trust them. A primary care provider: 4 = a great deal, 3 = somewhat, 2 = not too much, 1 = not at all’ (rescaled 0–1; *M* = 0.84, s.d. = 0.21)
4. Visit ER: ‘In the last 12 months, have you seen any of the following doctors … emergency room doctor?’ (*M* = 0.22; s.d. = 0.42)
5. ER adhere: ‘How closely would you say you have followed the following healthcare professionals’ advice or treatment recommendations in the last 12 months, such as taking medications as instructed, returning for follow-up appointments, and so on? Emergency room doctor: 5 = extremely closely, 4 = very closely, 3 = somewhat closely, 2 = not very closely, 1 = not at all closely’ (rescaled 0–1; *M* = 0.71, s.d. = 0.26)
6. ER trust: ‘For each of the following healthcare professionals that you have seen in the last 12 months, please indicate to what extent you trust them. Emergency room doctor: 4 = a great deal, 3 = somewhat, 2 = not too much, 1 = not at all’ (rescaled 0–1; *M* = 0.74, s.d. = 0.26)
7. Visit: ‘Chest pain: What would you do in the following hypothetical situation? Imagine that you suddenly felt a pain in your chest while carrying something heavy. The pain is so bad it makes you feel sick. The pain wears off after a few minutes. How likely would you be to schedule an appointment with your primary care doctor about this? Please adjust the slider from 0 (not at all likely) to 10 (extremely likely).’ (rescaled 0–1; *M* = 0.66, s.d. = 0.3)
8. BMQ: An average of the following four questions: ‘my life would be impossible without my [fill in disease] medication’; ‘without medication for my [fill in disease], I would be very ill’; ‘I sometimes worry about the long-term effects of my [fill in disease] medication’; and ‘medication for my [fill in disease] disrupts my life’. Respondents could answer on a 5-point scale recoded from 0 = strongly agree to 1 = strongly disagree. For analysis, the scale is ordered so that higher values represent more belief in the medicine’s efficacy or safety. (*M* = 0.57; s.d. = 0.17).

Figure 5 shows the results of regressing the outcome on the *y* axis on the political measure in the facet label, rescaled to range from 0 to 1 (vote choice is binary for Biden or Trump; partisanship is a 7-point scale from ‘strong Democrat’ to ‘strong Republican’; ideology is a 7-point scale from extremely liberal to extremely conservative (the latter 2 are rescaled from 0 to 1). Models control for education (BA+; coded 1 if respondent reports having a college degree or more, 0 otherwise), race/ethnicity (dummy variables for white, Black, Hispanic and other—white respondents are the omitted category in regression), gender (male or female, as provided by the vendor), dummy variable for household income (over $100,000, between $25,000 and $100,000, and less than $25,000—the last category is omitted in the regression), dummy variable for health insurance status, and dummy variable for rurality (coded 1 if the respondent lives in a micropolitan or non-core area as defined by the 2013 NCHS Urban-Rural Classification). Full model results are available in Supplementary Section 8 along with bivariate regressions and results for those with a chronic illness.

### Reporting summary

Further information on research design is available in the Nature Portfolio Reporting Summary linked to this article.

## Supplementary information

Supplementary Information All supplementary information.

Reporting Summary

Peer Review file

## Data Availability

Add Health restricted-use data are available through contractual agreement, subject to data security and research justification requirements, through the process detailed at https://data.cpc.unc.edu/projects/2/view; to protect participants’ sensitive health data, these cannot be shared publicly. Original survey data are available at https://osf.io/q3gn5.

## Code availability

Code for all analysis is available at https://osf.io/q3gn5. Analyses were conducted using R v.4.4.0 and Stata 17.

## References

[1] Warraich, H. J., Kumar, P., Nasir, K., Maddox, K. E. J. & Wadhera, R. K. Political environment and mortality rates in the United States, 2001–19: population based cross sectional analysis. *BMJ* **377**, e069308 (2022). 10.1136/bmj-2021-069308 PMC9171631 35672032

[2] Case, A. & Deaton, A. The great divide: education, despair, and death. *Annu. Rev. Econ.* **14**, 1–21 (2022). 10.1146/annurev-economics-051520-015607 PMC9389919 35990244

[3] Monnat, S. M. & Brown, D. L. More than a rural revolt: landscapes of despair and the 2016 presidential election. *J. Rural Stud.* **55**, 227–236 (2017). 29269990 10.1016/j.jrurstud.2017.08.010 PMC5734668

[4] Bor, J. Diverging life expectancies and voting patterns in the 2016 US presidential election. *Am. J. Public Health* **107**, 1560–1562 (2017). 28817322 10.2105/AJPH.2017.303945 PMC5607673

[5] Torche, F. & Rauf, T. The political context and infant health in the United States. *Am. Sociol. Rev.* **86**, 377–405 (2021).

[6] Beland, L.-P. & Oloomi, S. Party affiliation and public spending: evidence from US governors. *Econ. Inq.* **55**, 982–995 (2017).

[7] Tung, G. J., Vernick, J. S., Stuart, E. A. & Webster, D. W. Political factors affecting the enactment of state-level clean indoor air laws. *Am. J. Public Health* **104**, e92–e97 (2014). 24825239 10.2105/AJPH.2013.301689 PMC4062011

[8] Montez, J. K. et al. US state policies, politics, and life expectancy. *Milbank Q.* **98**, 668–699 (2020). 32748998 10.1111/1468-0009.12469 PMC7482386

[9] Fox, A. M., Feng, W. & Yumkham, R. State political ideology, policies and health behaviors: the case of tobacco. *Soc. Sci. Med.* **181**, 139–147 (2017). 28395251 10.1016/j.socscimed.2017.03.056

[10] Pacheco, J. & Fletcher, J. Incorporating health into studies of political behavior: evidence for turnout and partisanship. *Political Res. Q.* **68**, 104–116 (2015). 10.1177/1065912914563548 PMC6042216 30008544

[11] Subramanian, S. & Perkins, J. M. Are Republicans healthier than Democrats? *Int. J. Epidemiol.* **39**, 930–931 (2010). 19264845 10.1093/ije/dyp152 PMC2912481

[12] Subramanian, S., Huijts, T. & Perkins, J. M. Association between political ideology and health in Europe. *Eur. J. Public Health* **19**, 455–457 (2009). 19535606 10.1093/eurpub/ckp077 PMC2764955

[13] Pabayo, R., Kawachi, I. & Muennig, P. Political party affiliation, political ideology and mortality. *J. Epidemiol. Community Health* **69**, 423–431 (2015). 25631861 10.1136/jech-2014-204803 PMC5893220

[14] Ebert, T., Berkessel, J. & Jonsson, T. Political person-culture match and longevity: the partisanship–mortality link depends on the cultural context. *Psychol. Sci.* **34**, 1192–1205 (2023). 37874332 10.1177/09567976231196145

[15] Kannan, V. D., Brown, T. M., Kunitz, S. J. & Chapman, B. P. Political parties and mortality: the role of social status and personal responsibility. *Soc. Sci. Med.* **223**, 1–7 (2019). 30684874 10.1016/j.socscimed.2019.01.029 PMC10352900

[16] Harris, K. M. The National Longitudinal Study of Adolescent to Adult Health (Add Health), Waves I & II, 1994–1996; Wave III, 2001–2002; Wave IV, 2007-2009; Wave V, 2016-2018 [machine-readable data file and documentation]. https://addhealth.cpc.unc.edu/data/restricted-use-data/ (Carolina Population Center, University of North Carolina at Chapel Hill, 2018).

[17] Fiorina, M. P. & Abrams, S. J. Political polarization in the American public. *Annu. Rev. Political Sci.* **11**, 563–588 (2008).

[18] Choi, Y. & Fox, A. M. Mistrust in public health institutions is a stronger predictor of vaccine hesitancy and uptake than trust in Trump. *Soc. Sci. Med.* **314**, 115440 (2022). 36332532 10.1016/j.socscimed.2022.115440 PMC9557136

[19] Motta, M. Republicans, not Democrats, are more likely to endorse anti-vaccine misinformation. *Am. Polit. Res.* **49**, 428–438 (2021).

[20] Kröger, H. & Hartmann, J. Extending the Kitagawa–Oaxaca–Blinder decomposition approach to panel data. *Stata J.* **21**, 360–410 (2021).

[21] Zajacova, A. & Lawrence, E. M. The relationship between education and health: reducing disparities through a contextual approach. *Annu. Rev. Public Health* **39**, 273–289 (2018). 29328865 10.1146/annurev-publhealth-031816-044628 PMC5880718

[22] Zingher, J. N. TRENDS: diploma divide: educational attainment and the realignment of the American electorate. *Polit. Res. Q.* **75**, 263–277 (2022).

[23] Wallace, J., Goldsmith-Pinkham, P. & Schwartz, J. L. *Excess Death Rates for Republicans and Democrats During the COVID-19 Pandemic*. Working Paper 30512 (National Bureau of Economic Research, 2022).

[24] Woolf, S. H. The growing influence of state governments on population health in the United States. *JAMA* **327**, 1331–1332 (2022). 10.1001/jama.2022.3785 PMC9745671 35275203

[25] Blendon, R. J. & Benson, J. M. Trust in medicine, the health system and public health. *Daedalus* **151**, 67–82 (2022).

[26] Brady, H. E. & Kent, T. B. Fifty years of declining confidence and increasing polarization in trust in American institutions. *Daedalus* **151**, 43–66 (2022).

[27] Burnier, M. Drug adherence in hypertension. *Pharmacol. Res.* **125**, 142–149 (2017). 28870498 10.1016/j.phrs.2017.08.015

[28] Adamson, J., Yoav, B.-S., Chaturvedi, N. & Donovan, J. Ethnicity, socio-economic position and gender–do they affect reported health–care seeking behaviour? *Soc. Sci. Med.* **57**, 895–904 (2003). 12850114 10.1016/s0277-9536(02)00458-6

[29] Horne, R., Weinman, J. & Hankins, M. The Beliefs about Medicines Questionnaire: the development and evaluation of a new method for assessing the cognitive representation of medication. *Psychol. Health* **14**, 1–24 (1999).

[30] Baum, M., Druckman, J., Lazer, D. & Ognyanova, K. P. I. The Civic Health and Institutions Project, a 50 States Survey (CHIP50). https://www.chip50.org/ (CHIP50, 2021).

[31] Hagger, M. S. et al. Effects of medication, treatment, and behavioral beliefs on intentions to take medication in patients with familial hypercholesterolemia. *Atherosclerosis* **277**, 493–501 (2018). 30270090 10.1016/j.atherosclerosis.2018.06.010

[32] Robinson, L. B. Beliefs about cholesterol lowering drugs and medication adherence among rural adults with hypercholeterolemia. *Online J. Rural Nurs. Health Care* **15**, 3–25 (2015).

