# Supplementary material for: The political polarization of health outcomes in the USA
Source: Nat Hum Behav. 2026 May 14;10(7):1234–45. doi: 10.1038/s41562-026-02474-9 (PMC13388103; doi:10.1038/s41562-026-02474-9)
Supplement: Supplementary file 1 — All supplementary information. [file 41562_2026_2474_MOESM1_ESM.pdf]

nature human behaviour

Supplementary information https://doi.org/10.1038/s41562-026-02474-9

The political polarization of health outcomes in the USA

In the format provided by the authors and unedited

Contents

1 Add Health Wave 4 & 5 Biomarker Data **2**
1.1 Individual Biomarkers 2
1.2 Biomarkers Regression Results 3

2 Detail: Biomarkers for Conservative Respondents **8**

3 Ideology and Death in Older Cohorts across their 30s **10**

4 Death Over Time: Alternative Models **11**

5 Death Over Time: Full Regression Results **21**

6 Internally Caused Death, Categorical Birth Year **23**

7 Predictors of Death, 2020-22 **25**

8 Multivariable Analysis: Trust and Engagement **32**

9 Correlates of Ideology **38**

10 Doctor Visits over Time **42**

11 Demographics **44**

12 Supplemental Information References **46**

1 Add Health Wave 4 & 5 Biomarker Data

1.1 Individual Biomarkers

Supplementary Figure 1 shows average levels of individual biomarkers by ideological group. Supplementary Table 1 shows figure 1's results in tabular form.

Supplementary Figure 1: Biomarkers by Ideological Self Identification: Wave 4 (2008–09) & Wave 5 (2016–18)

Wave	Outcome	Very Liberal	Liberal	Moderate	Conservative	Very Conservative
Wave 4	A1C: Diabetes	0.04 [0.02, 0.06] (0.01) N = 809	0.03 [0.02, 0.04] (0.01) N = 2725	0.04 [0.03, 0.05] (0.00) N = 5920	0.03 [0.02, 0.04] (0.01) N = 2643	0.04 [0.02, 0.07] (0.01) N = 592
Wave 4	BMI: Obese	0.38 [0.32, 0.43] (0.03) N = 860	0.34 [0.31, 0.36] (0.01) N = 2974	0.38 [0.36, 0.40] (0.01) N = 6349	0.35 [0.32, 0.38] (0.01) N = 2871	0.39 [0.33, 0.44] (0.03) N = 625
Wave 4	BP: Hypertension	0.19 [0.15, 0.23] (0.02) N = 847	0.19 [0.16, 0.21] (0.01) N = 2897	0.19 [0.18, 0.21] (0.01) N = 6252	0.20 [0.18, 0.23] (0.01) N = 2822	0.21 [0.16, 0.26] (0.03) N = 605
Wave 4	CRP: High	0.43 [0.37, 0.49] (0.03) N = 778	0.38 [0.35, 0.40] (0.01) N = 2661	0.41 [0.39, 0.43] (0.01) N = 5764	0.38 [0.35, 0.41] (0.01) N = 2584	0.36 [0.31, 0.41] (0.03) N = 572
Wave 4	Hyperlipidemia	0.05 [0.04, 0.07] (0.01) N = 880	0.09 [0.07, 0.10] (0.01) N = 3023	0.08 [0.07, 0.10] (0.01) N = 6446	0.08 [0.07, 0.10] (0.01) N = 2909	0.08 [0.05, 0.11] (0.02) N = 634
Wave 5	A1C: Diabetes	0.04 [-0.00, 0.08] (0.02) N = 327	0.03 [0.02, 0.05] (0.01) N = 949	0.06 [0.04, 0.07] (0.01) N = 1991	0.05 [0.02, 0.07] (0.01) N = 928	0.09 [0.03, 0.15] (0.03) N = 249
Wave 5	BMI: Obese	0.40 [0.33, 0.48] (0.04) N = 368	0.42 [0.37, 0.47] (0.02) N = 1062	0.49 [0.46, 0.52] (0.01) N = 2221	0.46 [0.41, 0.50] (0.02) N = 1050	0.52 [0.43, 0.60] (0.04) N = 278
Wave 5	BP: Hypertension	0.16 [0.11, 0.20] (0.02) N = 363	0.17 [0.14, 0.20] (0.02) N = 1048	0.22 [0.20, 0.24] (0.01) N = 2163	0.23 [0.18, 0.28] (0.02) N = 1022	0.25 [0.17, 0.32] (0.04) N = 272
Wave 5	CRP: High	0.33 [0.25, 0.41] (0.04) N = 316	0.33 [0.29, 0.37] (0.02) N = 909	0.38 [0.35, 0.41] (0.01) N = 1944	0.30 [0.26, 0.34] (0.02) N = 930	0.40 [0.32, 0.48] (0.04) N = 247
Wave 5	Hyperlipidemia	0.16 [0.11, 0.22] (0.03) N = 375	0.15 [0.12, 0.18] (0.02) N = 1083	0.18 [0.15, 0.20] (0.01) N = 2266	0.15 [0.12, 0.18] (0.02) N = 1065	0.21 [0.14, 0.28] (0.03) N = 284

Note. Health status by ideological self-identification in wave 4 (2008-09) and wave 5 (2016-18) of the Add health survey (i.e., ideology and health outcomes are measured seperately in both wave 4 and wave 5). Each cell contatins the average, 95% confidence interval in brackets, standard error in parentheses and N represents observations.

Supplementary Table 1: Comorbidity Index, Wave 4 & 5

	Ideology	Wave	Mean	SE	LB	UB	N
1	Very Lib	4	21.83	1.22	19.40	24.25	745
2	Lib	4	20.41	0.67	19.08	21.73	2538
3	Mod	4	22.42	0.46	21.51	23.34	5531
4	Con	4	21.29	0.66	19.99	22.59	2505
5	Very Con	4	22.17	1.30	19.61	24.74	546
6	Very Lib	5	22.50	1.76	19.03	25.97	289
7	Lib	5	21.95	1.09	19.79	24.11	853
8	Mod	5	26.27	0.71	24.86	27.68	1802
9	Con	5	23.32	1.36	20.62	26.02	872
10	Very Con	5	30.53	2.51	25.56	35.51	224

The table displays results shown in Figure 1, but in tabular form (expressed as a percent out of 100)

1.2 Biomarkers Regression Results

This section includes full statistical reporting for the patterns in biomarkers discussed in the main text. Supplementary Table 2 shows the results of regressing the comorbidity index on ideology in waves 4 and 5, for respondents with and without missing biomarker data. Supplementary Table 3 shows the difference in wave 4-to-5 differences in health across health groups. Supplementary Table 4 presents an equivalence test showing the differences in comorbidities between ideological groups in wave 4 were null. Supplementary Table 5 presents regression results comparable to figure 2: columns 1-4 regress wave 5 ideology on wave 4 health (and ideology), while columns 5-8 regress wave 5 health on wave 4 ideology (and health).

Supplementary Table 2: Biomarkers by Ideology, Regression

	Wave 4		Wave 5	
	(1) All Rs	(2) No Missing	(3) All Rs	(4) No Missing
Liberal	-0.014	0.014	-0.006	-0.011
SE	(0.011)	(0.019)	(0.021)	(0.022)
Pvalue	0.212	0.453	0.789	0.602
95% CI	[-0.04,0.01]	[-0.02,0.05]	[-0.05,0.04]	[-0.05,0.03]
Moderate	0.006	0.033	0.038	0.046
SE	(0.011)	(0.017)	(0.018)	(0.020)
Pvalue	0.602	0.055	0.043	0.023
95% CI	[-0.02,0.03]	[-0.00,0.07]	[0.00,0.07]	[0.01,0.09]
Conservative	-0.005	0.013	0.008	0.014
SE	(0.013)	(0.019)	(0.022)	(0.023)
Pvalue	0.685	0.489	0.713	0.550
95% CI	[-0.03,0.02]	[-0.02,0.05]	[-0.04,0.05]	[-0.03,0.06]
Very Conservative	0.003	0.004	0.080	0.078
SE	(0.017)	(0.026)	(0.032)	(0.036)
Pvalue	0.843	0.872	0.014	0.031
95% CI	[-0.03,0.04]	[-0.05,0.06]	[0.02,0.14]	[0.01,0.15]
Constant	0.218	0.197	0.225	0.228
SE	(0.012)	(0.018)	(0.018)	(0.018)
Pvalue	0.000	0.000	0.000	0.000
95% CI	[0.19,0.24]	[0.16,0.23]	[0.19,0.26]	[0.19,0.26]
N	11,865	3,240	4,040	3,349
FE				

Regression of comorbidity index on self-reported ideology. Very liberal respondents are the omittted category. Columns 1 and 3 are the data from Figure 1 in the main text (only respondents who are not missing a measurement are included in these results). Columns 2 and 4 include only respondents who have all five measurements available in both waves and have reported their ideology in both waves. The observations slightly differ between columns 2 and 4 due to a handful of wave 4 respondents missing survey weights in wave 4. This is due to sampling design of Add Health (see `https://addhealth.cpc.unc.edu/documentation/frequently-asked-questions/`).

Supplementary Table 3: Difference-in-Difference, Wave 5 - Wave 4

	(1)
Very Liberal	0.007
SE	(0.020)
Pvalue	0.740
95% CI	[-0.03,0.05]
Liberal	0.009
SE	(0.024)
Pvalue	0.720
95% CI	[-0.04,0.06]
Moderate	0.032
SE	(0.021)
Pvalue	0.127
95% CI	[-0.01,0.07]
Conservative	0.014
SE	(0.026)
Pvalue	0.599
95% CI	[-0.04,0.06]
Very Conservative	0.077
SE	(0.035)
Pvalue	0.028
95% CI	[0.01,0.15]
N	15,905

Table represents the differences between the given ideological sub-group and very liberal respondents between wave 4 and wave 5. For example, the bottom cell means that the gap between very conservative and Very liberal respondents in wave 5 was .077 points larger than the difference between very liberal and very conservative respondents in wave 4. The coefficient on very liberal is simply the difference in comorbidities among very liberal respondents between waves 4 and 5.

Supplementary Table 4: Wave 4 Equivalence Test

	(1)
Liberal	-0.014
SE	(0.011)
Pvalue	0.212
90% CI	[-0.03,0.00]
Moderate	0.006
SE	(0.011)
Pvalue	0.602
90% CI	[-0.01,0.02]
Conservative	-0.005
SE	(0.013)
Pvalue	0.685
90% CI	[-0.03,0.02]
Very Conservative	0.003
SE	(0.017)
Pvalue	0.843
90% CI	[-0.03,0.03]
Constant	0.218
SE	(0.012)
Pvalue	0.000
90% CI	[0.20,0.24]
N	11,865

Table presents 90% confidence bounds which facilitate two one-sided tests of equivalence. Let's presume the null here is that the effect is larger than .04 and less than -.04. The null hypothesis can be rejected at .05 level if the upper and lower bounds of the 90% confidence interval fall within the range of (-.04,.04).

Supplementary Table 5: Health & Ideology Between W4 and W5

	DV=W5 Ideology				DV=W5 Comorbidities			
	(1) No Miss	(2) No Miss	(3) Miss 1	(4) Miss 1	(5) No Miss	(6) No Miss	(7) Miss 1	(8) Miss 1
W4 Comorbidities	0.044	0.029	0.052	0.034	0.596	0.571	0.634	0.610
SE	(0.022)	(0.025)	(0.021)	(0.023)	(0.023)	(0.023)	(0.021)	(0.022)
Pvalue	0.049	0.244	0.013	0.136	0.000	0.000	0.000	0.000
95% CI	[0.00,0.09]	[-0.02,0.08]	[0.01,0.09]	[-0.01,0.08]	[0.55,0.64]	[0.52,0.62]	[0.59,0.68]	[0.57,0.65]
W4 Ideology	0.594	0.584	0.591	0.581	0.003	-0.008	-0.005	-0.013
SE	(0.026)	(0.024)	(0.026)	(0.024)	(0.023)	(0.024)	(0.020)	(0.021)
Pvalue	0.000	0.000	0.000	0.000	0.902	0.754	0.814	0.542
95% CI	[0.54,0.65]	[0.54,0.63]	[0.54,0.64]	[0.53,0.63]	[-0.04,0.05]	[-0.05,0.04]	[-0.04,0.04]	[-0.05,0.03]
Birth Year		-0.003		-0.002		-0.001		-0.002
SE		(0.003)		(0.003)		(0.003)		(0.003)
Pvalue		0.318		0.502		0.774		0.384
95% CI		[-0.01,0.00]		[-0.01,0.00]		[-0.01,0.01]		[-0.01,0.00]
Inc≥100k		0.013		0.013		-0.027		-0.032
SE		(0.018)		(0.016)		(0.017)		(0.015)
Pvalue		0.467		0.445		0.113		0.032
95% CI		[-0.02,0.05]		[-0.02,0.04]		[-0.06,0.01]		[-0.06,-0.00]
30k< Inc <100k		0.015		0.008		-0.008		-0.012
SE		(0.014)		(0.014)		(0.013)		(0.012)
Pvalue		0.306		0.573		0.572		0.340
95% CI		[-0.01,0.04]		[-0.02,0.03]		[-0.03,0.02]		[-0.04,0.01]
Hlth Ins		0.019		0.021		-0.016		-0.011
SE		(0.011)		(0.012)		(0.010)		(0.008)
Pvalue		0.106		0.083		0.100		0.158
95% CI		[-0.00,0.04]		[-0.00,0.05]		[-0.03,0.00]		[-0.03,0.00]
Male		0.015		0.010		0.004		0.005
SE		(0.010)		(0.010)		(0.011)		(0.010)
Pvalue		0.145		0.323		0.710		0.592
95% CI		[-0.01,0.04]		[-0.01,0.03]		[-0.02,0.03]		[-0.01,0.03]
BA+		-0.028		-0.034		-0.051		-0.048
SE		(0.010)		(0.010)		(0.011)		(0.010)
Pvalue		0.006		0.001		0.000		0.000
95% CI		[-0.05,-0.01]		[-0.05,-0.01]		[-0.07,-0.03]		[-0.07,-0.03]
Black		0.007		0.007		0.041		0.043
SE		(0.016)		(0.013)		(0.017)		(0.015)
Pvalue		0.665		0.622		0.020		0.005
95% CI		[-0.03,0.04]		[-0.02,0.03]		[0.01,0.07]		[0.01,0.07]
Hispanic		-0.006		-0.011		-0.014		-0.013
SE		(0.019)		(0.017)		(0.018)		(0.016)
Pvalue		0.737		0.498		0.434		0.417
95% CI		[-0.04,0.03]		[-0.04,0.02]		[-0.05,0.02]		[-0.05,0.02]
Other Race		-0.018		-0.017		0.026		0.021
SE		(0.017)		(0.017)		(0.022)		(0.020)
Pvalue		0.287		0.314		0.242		0.281
95% CI		[-0.05,0.02]		[-0.05,0.02]		[-0.02,0.07]		[-0.02,0.06]
Rural		0.045		0.043		0.008		0.008
SE		(0.010)		(0.009)		(0.010)		(0.009)
Pvalue		0.000		0.000		0.405		0.376
95% CI		[0.03,0.06]		[0.02,0.06]		[-0.01,0.03]		[-0.01,0.02]
Constant	0.206	5.634	0.203	3.711	0.122	1.928	0.118	4.942
SE	(0.015)	(5.445)	(0.015)	(5.228)	(0.013)	(6.121)	(0.011)	(5.477)
Pvalue	0.000	0.303	0.000	0.479	0.000	0.753	0.000	0.369
95% CI	[0.18,0.24]	[-5.14,16.41]	[0.17,0.23]	[-6.63,14.06]	[0.10,0.15]	[-10.18,14.04]	[0.10,0.14]	[-5.89,15.78]
N	3,349	3,201	3,973	3,791	3,349	3,201	3,973	3,791

Columns 1-4: DV = Wave 5 ideology. Columsn 4-8: DV = Comorbidity index. Data include results for respondents who are missing no comoribidity measurements (titled “No miss”) or have 4 of 5 possible comorbidities measured (titled “Miss 1”).

2 Detail: Biomarkers for Conservative Respondents

Figure 1 in the main text shows that Add Health respondents who identified as very conservative in wave 5 were less healthy than their peers, and less healthy than this group had been in wave 4. Though the very conservative respondents appear distinctive in that analysis, the remaining analyses of biomarkers group together very conservative respondents with conservative respondents for the sake of power.

To understand whether very conservative respondents displayed distinctive patterns of change from conservative respondents, Supplementary Figure 2 shows the distribution of biomarkers for respondents who identified as very conservative (top panel) or conservative (bottom panel) in wave 5, separately by their ideology in wave 4. The top left panel contains too few observations to be plotted without raising concerns about individual identification (as mandated by Add Health), so it is left blank.

In broad strokes, these figures suggest that combining the conservative and very conservative respondents in Supplementary Figure 2 does not substantively affect our conclusions. As the sample sizes shown in each segment indicate, though, many of these cells are very small; conclusions should not be drawn about individual groups.

First, the "stayed conservative" panel in Supplementary Figure 2 is made up of the right-most two segments in the top and bottom panels of figure 2. The more detailed figures suggest that, as in the combined plot, people who remained (very) conservative in both waves were slightly less healthy in wave 5 than wave 4.

Second, the "became conservative" panel in figure 2 is made up of the left-most two segments in the top and bottom panels of Supplementary Figure 2. As in the combined plot, people who switched from being either liberal group to either conservative group became less healthy between waves 4 and 5, though this change is quite small among people who switched from being merely liberal to merely conservative (rather rather than "very").

Supplementary Figure 2: Biomarkers for Conservative Respondents

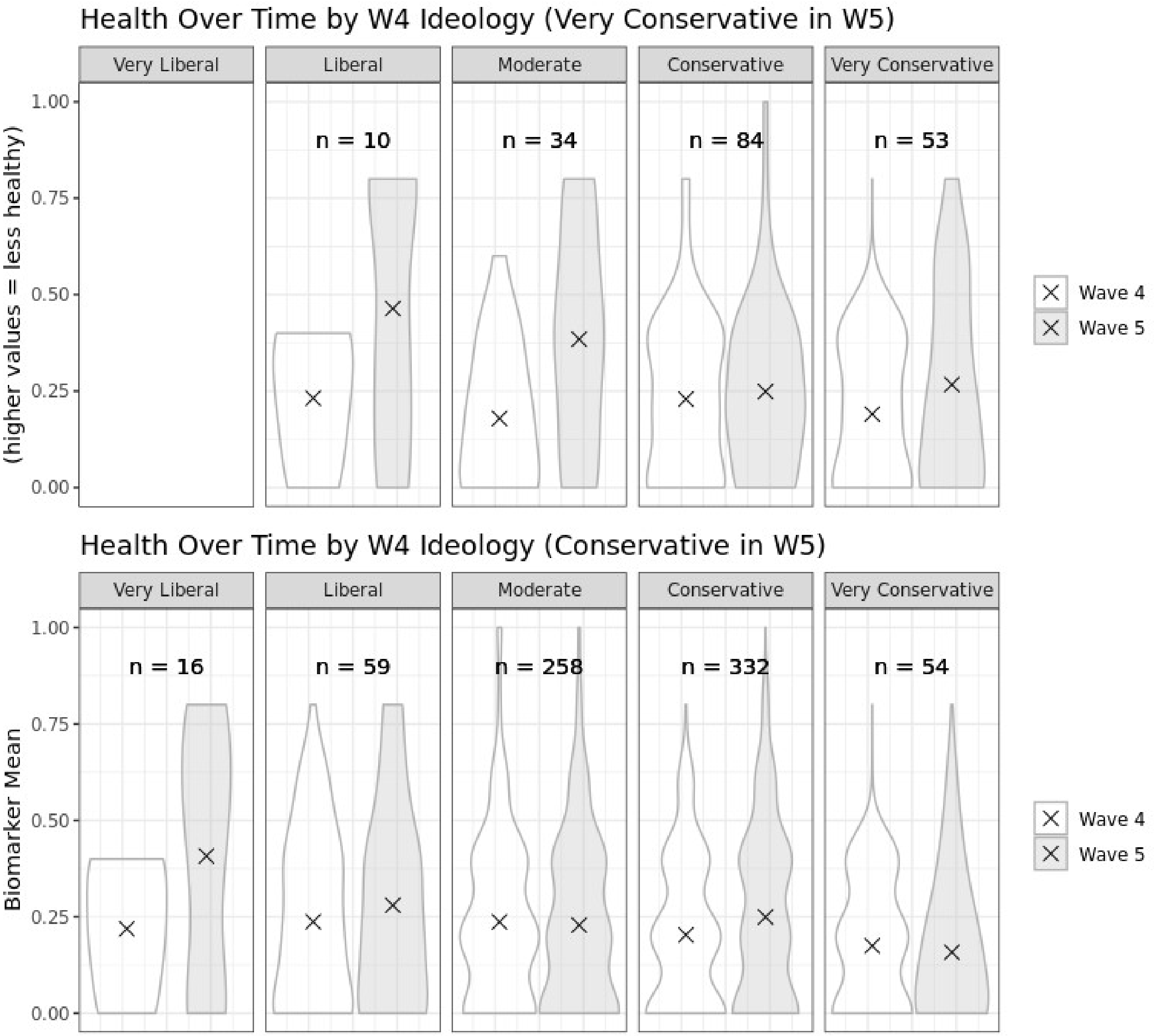

Note. Health in waves 4 and 5 among people who were very conservative or conservative in wave 5, divided by their ideology in wave 4. Health is measured by a comorbidity index, a combined measure of the 5 health indicators for respondents with each measure, so higher levels indicate worse health. Means for each group-wave are marked with an X; density is underlaid, with 25th, 50th, and 75th percentiles marked with gray lines. Top left panel is omitted due to privacy concerns.

3 Ideology and Death in Older Cohorts across their 30s

This section examines the relationship between ideology and death across cohorts as they age through their 20s, 30s and 40s (paralleling the analysis through the time-frame available through the Add Health cohort). Data are from the General Social Survey (Davern et al., 2025) which linked respondents who took the GSS between 1978-2010 to the National Death Index from 1978-2014.

There are difficulties to comparing this cross-sectional data-set with the Add health longitudinal data as ideology, for example, is measured at different years whereas it is measured at the same year in Add health. Likewise, people may die before they enter the survey. Relatedly, to parallel the analysis of the Add health cohort, examining older cohorts means that people who show up in the sample are conditional on survival (e.g., someone born in 1950, would have to have lived until 38 years of age before being surveyed in 1978, the first year which NDI data are linked to the sample).

Supplementary Figure 3: Ideology and Internally Caused Deaths

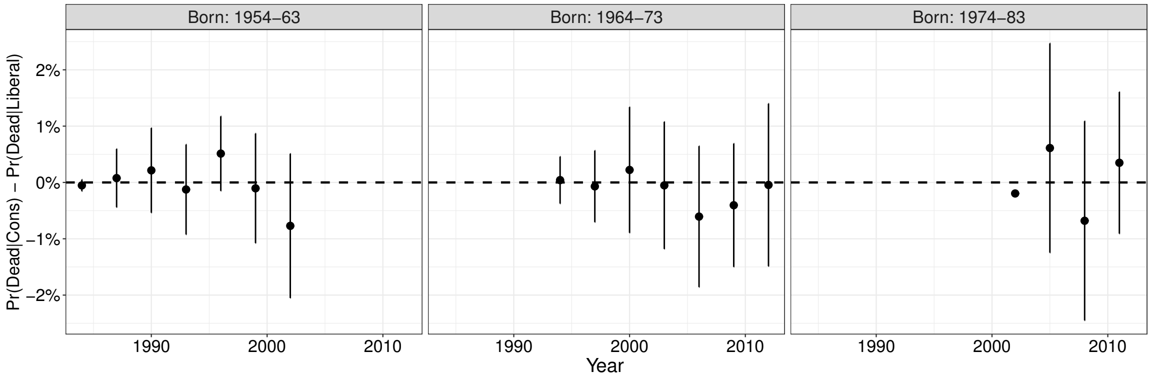

Note. As in the main paper, the analysis is presented in 3-year bins. Ideology is re-scaled from 0-1. The dependent variable, death in the three year bin, is coded 1 if the respondent died from an internal cause in the window, and 0 if they are alive.

4 Death Over Time: Alternative Models

This section presents three alternative specifications of the results in figure 4 in the main text: regressing cumulative deaths that occurred up to the given year (rather than three-year bins) on ideology, (Supplementary Figure 4), measuring deaths in each individual year rather than three-year bins, (Supplementary Figure 5, and using a logit (rather than linear probability) model (Supplementary Figure 6).

Supplementary Figure 4: Mortality Rate by Ideological Self-Identification: Cumulative

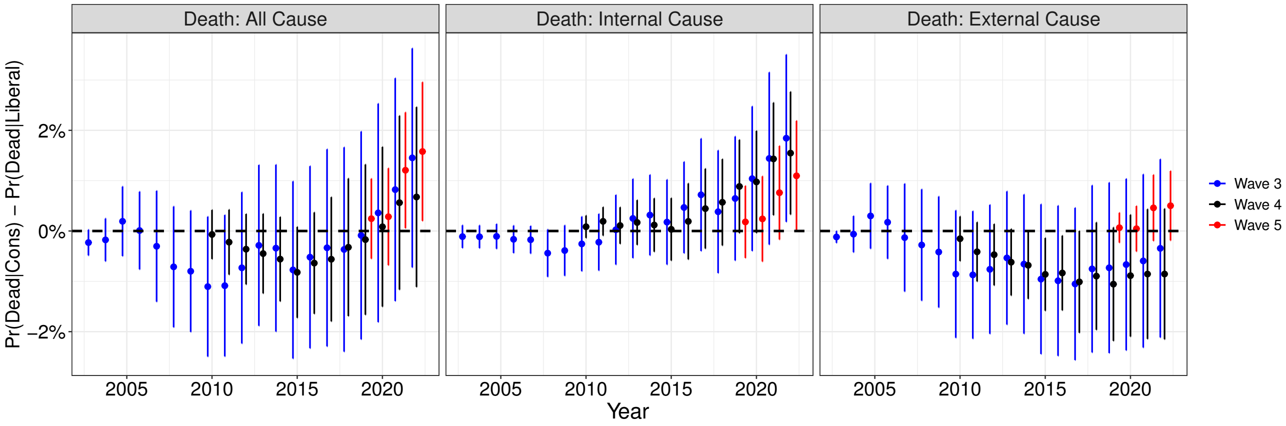

Note. Each point is the cumulative death rate by ideological self-identification by a given year labeled on the x-axis. Binary indicator of death (0-1) is regressed five-point ideological scale (using OLS). Higher values represent higher mortality rates for conservatives. Lines represent 95% confidence intervals (two-sided t-test); standard errors are clustered at the school level (sampling unit). For example, the right-most points (at 2022), shows the relationship between ideological as measured in Waves 3-5 for all deaths up to 2022. Ideological identification ranges from very liberal (coded 0) to very conservative (coded 1) as measured in wave 3, 4 and 5.

All Caused Deaths: Cumulative

	Year	Wave	Coef	SE	Pvalue	LB	UB	N
1	2003	3	-0.23	0.13	0.08	-0.48	0.03	12962
2	2004	3	-0.18	0.21	0.41	-0.60	0.25	12962
3	2005	3	0.19	0.35	0.58	-0.50	0.88	12962
4	2006	3	0.01	0.39	0.98	-0.76	0.78	12962
5	2007	3	-0.30	0.56	0.59	-1.40	0.80	12962
6	2008	3	-0.71	0.61	0.24	-1.91	0.49	12962
7	2009	3	-0.80	0.61	0.19	-2.00	0.40	12962
8	2010	3	-1.11	0.70	0.12	-2.49	0.28	12962
9	2011	3	-1.09	0.71	0.13	-2.49	0.32	12962
10	2012	3	-0.73	0.76	0.34	-2.23	0.77	12962
11	2013	3	-0.29	0.81	0.72	-1.88	1.31	12962
12	2014	3	-0.34	0.84	0.69	-2.00	1.32	12962
13	2015	3	-0.77	0.89	0.39	-2.53	0.99	12962
14	2016	3	-0.52	0.91	0.57	-2.33	1.29	12962
15	2017	3	-0.33	0.99	0.74	-2.29	1.62	12962
16	2018	3	-0.37	1.03	0.72	-2.40	1.66	12962
17	2019	3	-0.09	1.04	0.93	-2.15	1.98	12962
18	2020	3	0.36	1.10	0.74	-1.81	2.53	12962
19	2021	3	0.82	1.12	0.46	-1.39	3.04	12962
20	2022	3	1.45	1.10	0.19	-0.72	3.63	12962
21	2010	4	-0.07	0.25	0.78	-0.56	0.42	13892
22	2011	4	-0.22	0.33	0.50	-0.87	0.42	13892
23	2012	4	-0.36	0.35	0.31	-1.06	0.34	13892
24	2013	4	-0.45	0.40	0.26	-1.24	0.34	13892
25	2014	4	-0.56	0.42	0.19	-1.40	0.28	13892
26	2015	4	-0.82	0.46	0.07	-1.72	0.08	13892
27	2016	4	-0.64	0.51	0.21	-1.64	0.37	13892
28	2017	4	-0.56	0.62	0.37	-1.79	0.67	13892
29	2018	4	-0.32	0.69	0.64	-1.69	1.04	13892
30	2019	4	-0.17	0.75	0.82	-1.66	1.32	13892
31	2020	4	0.08	0.80	0.92	-1.50	1.67	13892
32	2021	4	0.56	0.87	0.52	-1.16	2.29	13892
33	2022	4	0.68	0.90	0.46	-1.11	2.46	13892
34	2019	5	0.24	0.40	0.54	-0.55	1.04	11250
35	2020	5	0.28	0.49	0.56	-0.68	1.25	11250
36	2021	5	1.21	0.58	0.04	0.06	2.36	11250
37	2022	5	1.58	0.70	0.03	0.20	2.96	11250

Internally Caused Deaths: Cumulative

	Year	Wave	Coef	SE	Pvalue	LB	UB	N
1	2003	3	-0.11	0.11	0.33	-0.34	0.11	12947
2	2004	3	-0.11	0.11	0.32	-0.34	0.11	12935
3	2005	3	-0.11	0.12	0.39	-0.35	0.14	12927
4	2006	3	-0.17	0.14	0.23	-0.44	0.11	12918
5	2007	3	-0.17	0.14	0.21	-0.45	0.10	12911
6	2008	3	-0.44	0.24	0.07	-0.91	0.03	12905
7	2009	3	-0.39	0.25	0.13	-0.89	0.11	12900
8	2010	3	-0.26	0.27	0.35	-0.80	0.29	12892
9	2011	3	-0.22	0.28	0.44	-0.78	0.34	12886
10	2012	3	0.02	0.35	0.94	-0.67	0.72	12876
11	2013	3	0.25	0.40	0.53	-0.54	1.03	12868
12	2014	3	0.32	0.40	0.43	-0.48	1.12	12863
13	2015	3	0.18	0.43	0.68	-0.67	1.02	12855
14	2016	3	0.47	0.46	0.31	-0.44	1.37	12846
15	2017	3	0.72	0.56	0.20	-0.40	1.84	12831
16	2018	3	0.38	0.62	0.53	-0.84	1.60	12821
17	2019	3	0.65	0.62	0.30	-0.59	1.88	12810
18	2020	3	1.04	0.73	0.15	-0.40	2.48	12800
19	2021	3	1.44	0.86	0.10	-0.27	3.15	12789
20	2022	3	1.84	0.84	0.03	0.18	3.50	12776
21	2010	4	0.08	0.11	0.45	-0.14	0.31	13874
22	2011	4	0.19	0.14	0.18	-0.09	0.47	13866
23	2012	4	0.11	0.18	0.55	-0.25	0.47	13858
24	2013	4	0.17	0.22	0.45	-0.27	0.61	13846
25	2014	4	0.12	0.27	0.66	-0.41	0.65	13842
26	2015	4	0.03	0.31	0.92	-0.59	0.65	13830
27	2016	4	0.19	0.38	0.62	-0.56	0.94	13820
28	2017	4	0.44	0.40	0.27	-0.35	1.24	13805
29	2018	4	0.57	0.43	0.19	-0.29	1.43	13793
30	2019	4	0.89	0.47	0.06	-0.04	1.81	13781
31	2020	4	0.98	0.51	0.06	-0.04	1.99	13769
32	2021	4	1.43	0.56	0.01	0.32	2.55	13755
33	2022	4	1.55	0.62	0.01	0.33	2.77	13741
34	2019	5	0.18	0.36	0.62	-0.54	0.90	11229
35	2020	5	0.24	0.43	0.57	-0.61	1.09	11223
36	2021	5	0.76	0.47	0.11	-0.17	1.69	11214
37	2022	5	1.10	0.55	0.05	0.00	2.19	11208

Externally Caused Deaths: Cumulative

	Year	Wave	Coef	SE	Pvalue	LB	UB	N
1	2003	3	-0.12	0.06	0.05	-0.24	-0.00	12958
2	2004	3	-0.06	0.18	0.73	-0.42	0.30	12955
3	2005	3	0.30	0.33	0.36	-0.35	0.95	12950
4	2006	3	0.17	0.37	0.64	-0.55	0.90	12949
5	2007	3	-0.13	0.54	0.81	-1.20	0.94	12948
6	2008	3	-0.28	0.56	0.62	-1.38	0.83	12941
7	2009	3	-0.42	0.56	0.46	-1.52	0.68	12931
8	2010	3	-0.86	0.64	0.18	-2.12	0.41	12928
9	2011	3	-0.87	0.64	0.18	-2.14	0.39	12922
10	2012	3	-0.76	0.65	0.24	-2.04	0.52	12913
11	2013	3	-0.54	0.67	0.42	-1.86	0.79	12909
12	2014	3	-0.66	0.70	0.35	-2.04	0.72	12897
13	2015	3	-0.96	0.75	0.21	-2.44	0.53	12893
14	2016	3	-0.99	0.75	0.19	-2.48	0.50	12879
15	2017	3	-1.05	0.76	0.17	-2.56	0.46	12860
16	2018	3	-0.75	0.84	0.37	-2.41	0.91	12847
17	2019	3	-0.73	0.86	0.39	-2.42	0.96	12832
18	2020	3	-0.67	0.86	0.44	-2.37	1.04	12816
19	2021	3	-0.59	0.87	0.50	-2.32	1.13	12788
20	2022	3	-0.34	0.89	0.70	-2.11	1.43	12767
21	2010	4	-0.15	0.22	0.49	-0.60	0.29	13876
22	2011	4	-0.41	0.30	0.17	-1.00	0.17	13869
23	2012	4	-0.47	0.31	0.13	-1.08	0.14	13862
24	2013	4	-0.62	0.33	0.07	-1.28	0.04	13857
25	2014	4	-0.68	0.34	0.05	-1.35	-0.02	13843
26	2015	4	-0.86	0.36	0.02	-1.58	-0.14	13837
27	2016	4	-0.83	0.37	0.03	-1.58	-0.09	13826
28	2017	4	-1.01	0.51	0.05	-2.02	-0.00	13807
29	2018	4	-0.90	0.54	0.10	-1.96	0.17	13791
30	2019	4	-1.06	0.57	0.07	-2.18	0.07	13776
31	2020	4	-0.89	0.61	0.15	-2.10	0.32	13755
32	2021	4	-0.86	0.65	0.19	-2.14	0.43	13730
33	2022	4	-0.85	0.66	0.19	-2.15	0.44	13708
34	2019	5	0.07	0.15	0.66	-0.23	0.36	11225
35	2020	5	0.05	0.23	0.84	-0.41	0.50	11208
36	2021	5	0.46	0.33	0.17	-0.20	1.12	11186
37	2022	5	0.50	0.35	0.15	-0.19	1.19	11168

Supplementary Figure 5: Death Over Time: Deaths by Year

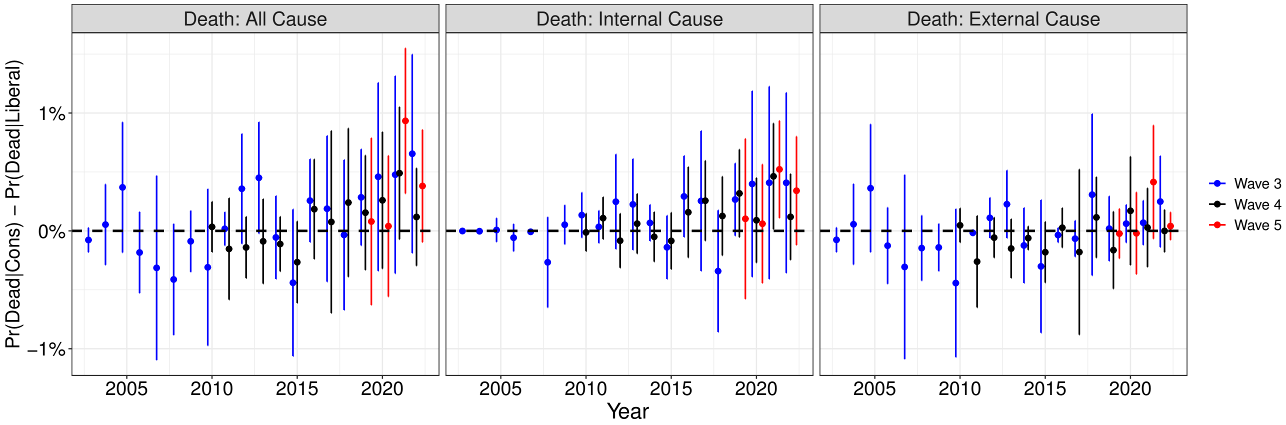

Note. Results replicate over-time death, but just by year. Coefficient is the linear probability of death (broken down by cause of death) when moving from most liberal to most conservative (ideology measured separately in wave 3, 4 and 5) in a given year. This figure differs from Figure 4 of the main text, which analyzes deaths in three-year chunks.

All Caused Deaths: Yearly

	Year	Wave	Coef	SE	Pvalue	LB	UB	N
1	2003	3	-0.08	0.05	0.14	-0.18	0.03	12950
2	2004	3	0.05	0.17	0.76	-0.29	0.39	12943
3	2005	3	0.37	0.28	0.19	-0.18	0.92	12928
4	2006	3	-0.18	0.17	0.29	-0.53	0.16	12915
5	2007	3	-0.31	0.39	0.43	-1.10	0.47	12905
6	2008	3	-0.41	0.24	0.09	-0.88	0.06	12897
7	2009	3	-0.09	0.13	0.50	-0.35	0.17	12884
8	2010	3	-0.31	0.34	0.36	-0.97	0.35	12869
9	2011	3	0.02	0.07	0.81	-0.12	0.16	12858
10	2012	3	0.36	0.24	0.13	-0.11	0.82	12846
11	2013	3	0.45	0.24	0.06	-0.02	0.92	12827
12	2014	3	-0.06	0.18	0.76	-0.41	0.30	12815
13	2015	3	-0.44	0.31	0.16	-1.06	0.18	12798
14	2016	3	0.26	0.18	0.15	-0.10	0.61	12786
15	2017	3	0.19	0.31	0.55	-0.43	0.81	12763
16	2018	3	-0.03	0.32	0.92	-0.67	0.60	12729
17	2019	3	0.28	0.21	0.17	-0.12	0.69	12706
18	2020	3	0.46	0.40	0.26	-0.34	1.26	12680
19	2021	3	0.48	0.42	0.26	-0.36	1.31	12654
20	2022	3	0.65	0.43	0.13	-0.19	1.50	12615
21	2010	4	0.03	0.11	0.75	-0.18	0.25	13869
22	2011	4	-0.15	0.22	0.48	-0.58	0.28	13858
23	2012	4	-0.14	0.13	0.29	-0.40	0.12	13843
24	2013	4	-0.09	0.18	0.63	-0.45	0.27	13828
25	2014	4	-0.11	0.12	0.34	-0.34	0.12	13811
26	2015	4	-0.27	0.17	0.13	-0.61	0.08	13793
27	2016	4	0.18	0.21	0.39	-0.24	0.61	13775
28	2017	4	0.08	0.39	0.85	-0.70	0.85	13754
29	2018	4	0.24	0.32	0.45	-0.39	0.87	13720
30	2019	4	0.15	0.25	0.53	-0.33	0.64	13692
31	2020	4	0.26	0.29	0.38	-0.32	0.84	13665
32	2021	4	0.49	0.28	0.09	-0.07	1.05	13632
33	2022	4	0.12	0.21	0.57	-0.30	0.53	13593
34	2019	5	0.08	0.36	0.82	-0.63	0.79	11222
35	2020	5	0.04	0.30	0.89	-0.56	0.64	11204
36	2021	5	0.93	0.31	0.00	0.32	1.55	11181
37	2022	5	0.38	0.24	0.12	-0.10	0.86	11150

Internally Caused Deaths: Yearly

	Year	Wave	Coef	SE	Pvalue	LB	UB	N
1	2003	3	-0.00	0.00	0.29	-0.00	0.00	12945
2	2004	3	-0.00	0.00	0.30	-0.01	0.00	12931
3	2005	3	0.01	0.05	0.88	-0.09	0.11	12920
4	2006	3	-0.06	0.06	0.32	-0.17	0.06	12906
5	2007	3	-0.01	0.01	0.38	-0.03	0.01	12898
6	2008	3	-0.27	0.19	0.17	-0.65	0.12	12891
7	2009	3	0.05	0.08	0.54	-0.11	0.22	12879
8	2010	3	0.13	0.10	0.17	-0.06	0.33	12861
9	2011	3	0.03	0.07	0.63	-0.10	0.17	12852
10	2012	3	0.25	0.20	0.23	-0.15	0.65	12836
11	2013	3	0.22	0.20	0.25	-0.16	0.61	12819
12	2014	3	0.07	0.08	0.38	-0.09	0.22	12810
13	2015	3	-0.14	0.14	0.31	-0.41	0.13	12790
14	2016	3	0.29	0.17	0.10	-0.05	0.64	12777
15	2017	3	0.25	0.30	0.40	-0.34	0.85	12748
16	2018	3	-0.34	0.26	0.19	-0.86	0.17	12719
17	2019	3	0.27	0.16	0.09	-0.04	0.57	12695
18	2020	3	0.40	0.40	0.32	-0.39	1.19	12670
19	2021	3	0.41	0.41	0.32	-0.41	1.22	12643
20	2022	3	0.41	0.39	0.29	-0.36	1.17	12602
21	2010	4	-0.01	0.08	0.87	-0.17	0.15	13861
22	2011	4	0.11	0.09	0.23	-0.07	0.29	13850
23	2012	4	-0.08	0.12	0.47	-0.31	0.15	13835
24	2013	4	0.06	0.13	0.63	-0.19	0.31	13816
25	2014	4	-0.05	0.11	0.64	-0.26	0.16	13807
26	2015	4	-0.09	0.12	0.48	-0.32	0.15	13781
27	2016	4	0.16	0.19	0.42	-0.23	0.54	13765
28	2017	4	0.26	0.17	0.14	-0.08	0.60	13739
29	2018	4	0.13	0.17	0.46	-0.21	0.46	13708
30	2019	4	0.32	0.19	0.09	-0.05	0.69	13680
31	2020	4	0.09	0.18	0.62	-0.27	0.45	13653
32	2021	4	0.46	0.23	0.04	0.01	0.91	13618
33	2022	4	0.12	0.18	0.52	-0.25	0.48	13579
34	2019	5	0.10	0.34	0.77	-0.58	0.78	11214
35	2020	5	0.06	0.25	0.81	-0.44	0.56	11198
36	2021	5	0.52	0.21	0.01	0.11	0.93	11172
37	2022	5	0.34	0.23	0.15	-0.12	0.80	11144

Externally Caused Deaths: Yearly

	Year	Wave	Coef	SE	Pvalue	LB	UB	N
1	2003	3	-0.08	0.05	0.15	-0.18	0.03	12948
2	2004	3	0.06	0.17	0.75	-0.29	0.40	12940
3	2005	3	0.36	0.27	0.19	-0.18	0.90	12923
4	2006	3	-0.13	0.16	0.44	-0.45	0.20	12914
5	2007	3	-0.31	0.39	0.44	-1.09	0.47	12904
6	2008	3	-0.15	0.14	0.30	-0.42	0.13	12890
7	2009	3	-0.14	0.10	0.17	-0.34	0.06	12874
8	2010	3	-0.44	0.32	0.17	-1.07	0.19	12866
9	2011	3	-0.02	0.01	0.17	-0.04	0.01	12852
10	2012	3	0.11	0.09	0.20	-0.06	0.28	12837
11	2013	3	0.23	0.15	0.12	-0.06	0.51	12823
12	2014	3	-0.12	0.16	0.44	-0.44	0.20	12803
13	2015	3	-0.30	0.28	0.29	-0.86	0.26	12794
14	2016	3	-0.04	0.03	0.26	-0.10	0.03	12772
15	2017	3	-0.07	0.08	0.39	-0.22	0.09	12744
16	2018	3	0.31	0.35	0.38	-0.38	0.99	12716
17	2019	3	0.02	0.14	0.89	-0.26	0.29	12691
18	2020	3	0.06	0.08	0.45	-0.10	0.22	12664
19	2021	3	0.07	0.09	0.47	-0.12	0.26	12626
20	2022	3	0.25	0.20	0.21	-0.14	0.64	12594
21	2010	4	0.05	0.07	0.52	-0.10	0.19	13866
22	2011	4	-0.26	0.20	0.19	-0.65	0.13	13851
23	2012	4	-0.06	0.09	0.51	-0.23	0.11	13836
24	2013	4	-0.15	0.13	0.24	-0.40	0.10	13823
25	2014	4	-0.06	0.05	0.23	-0.16	0.04	13797
26	2015	4	-0.18	0.13	0.17	-0.44	0.08	13787
27	2016	4	0.03	0.08	0.75	-0.14	0.19	13764
28	2017	4	-0.18	0.35	0.61	-0.88	0.52	13735
29	2018	4	0.11	0.17	0.51	-0.23	0.46	13704
30	2019	4	-0.16	0.17	0.32	-0.49	0.16	13677
31	2020	4	0.17	0.23	0.47	-0.29	0.63	13644
32	2021	4	0.03	0.17	0.87	-0.31	0.36	13607
33	2022	4	-0.00	0.09	0.99	-0.18	0.18	13571
34	2019	5	-0.02	0.11	0.83	-0.23	0.19	11212
35	2020	5	-0.02	0.18	0.91	-0.37	0.33	11187
36	2021	5	0.41	0.24	0.09	-0.07	0.90	11159
37	2022	5	0.04	0.06	0.49	-0.08	0.16	11132

Supplementary Figure 6: Death Over Time: Logit Model

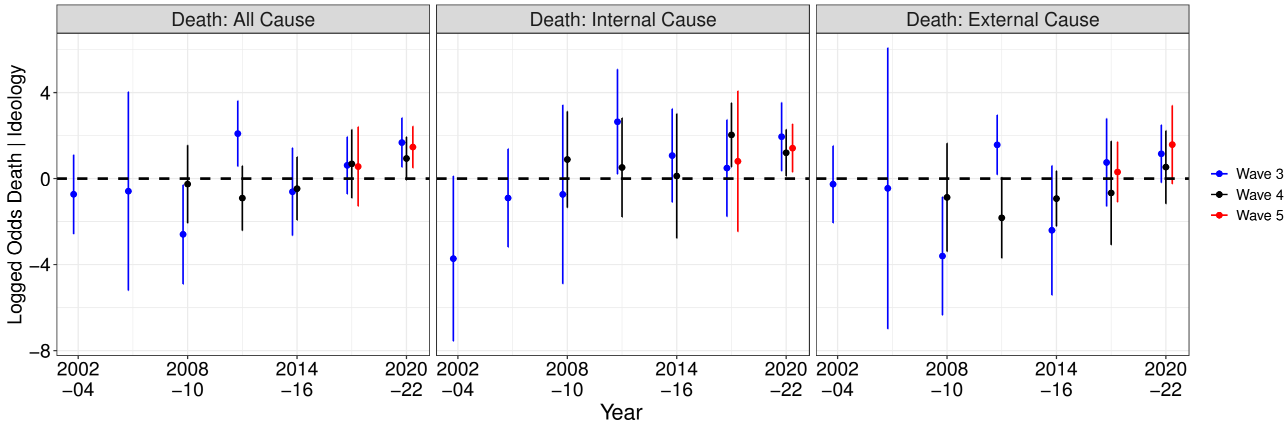

Note. Results replicate over-time death in text but using logit model. Coefficient is the logged odds of death (broken down by cause of death) when moving from most liberal to most conservative (ideology measured separately in wave 3, 4 and 5). Deaths are binned into three-year intervals. The table below provides full statistical reporting to accompany this figure.

All Caused Deaths: Logit

	Year	Wave	Coef	SE	Pvalue	LB	UB	N
1	2002	3	-0.73	0.93	0.43	-2.56	1.11	12959
2	2005	3	-0.59	2.33	0.80	-5.20	4.03	12928
3	2008	3	-2.59	1.17	0.03	-4.90	-0.28	12897
4	2011	3	2.09	0.77	0.01	0.57	3.62	12858
5	2014	3	-0.61	1.03	0.56	-2.65	1.43	12815
6	2017	3	0.62	0.67	0.36	-0.72	1.95	12763
7	2020	3	1.68	0.58	0.00	0.53	2.83	12680
8	2008	4	-0.26	0.91	0.78	-2.06	1.55	13892
9	2011	4	-0.91	0.76	0.24	-2.41	0.60	13858
10	2014	4	-0.47	0.74	0.53	-1.94	1.01	13811
11	2017	4	0.69	0.81	0.40	-0.91	2.28	13754
12	2020	4	0.94	0.51	0.07	-0.06	1.94	13665
13	2017	5	0.56	0.94	0.55	-1.29	2.41	11250
14	2020	5	1.47	0.49	0.00	0.50	2.43	11204

Internally Caused Deaths: Logit

	Year	Wave	Coef	SE	Pvalue	LB	UB	N
1	2002	3	-3.72	1.94	0.06	-7.55	0.11	12934
2	2005	3	-0.90	1.16	0.44	-3.19	1.39	12904
3	2008	3	-0.73	2.10	0.73	-4.89	3.42	12878
4	2011	3	2.64	1.23	0.03	0.20	5.08	12834
5	2014	3	1.07	1.10	0.33	-1.10	3.25	12793
6	2017	3	0.49	1.14	0.67	-1.76	2.74	12727
7	2020	3	1.95	0.80	0.02	0.36	3.54	12646
8	2008	4	0.89	1.13	0.43	-1.35	3.13	13874
9	2011	4	0.52	1.16	0.66	-1.78	2.82	13830
10	2014	4	0.12	1.47	0.93	-2.78	3.02	13785
11	2017	4	2.03	0.75	0.01	0.55	3.52	13715
12	2020	4	1.20	0.55	0.03	0.12	2.29	13625
13	2017	5	0.81	1.65	0.63	-2.46	4.08	11229
14	2020	5	1.42	0.57	0.01	0.30	2.54	11183

Externally Caused Deaths: Logit

	Year	Wave	Coef	SE	Pvalue	LB	UB	N
1	2002	3	-0.26	0.91	0.77	-2.05	1.53	12953
2	2005	3	-0.45	3.30	0.89	-6.99	6.08	12921
3	2008	3	-3.60	1.39	0.01	-6.35	-0.86	12877
4	2011	3	1.57	0.70	0.03	0.19	2.96	12839
5	2014	3	-2.40	1.52	0.12	-5.42	0.61	12785
6	2017	3	0.75	1.03	0.47	-1.29	2.80	12716
7	2020	3	1.16	0.68	0.09	-0.18	2.49	12615
8	2008	4	-0.87	1.27	0.49	-3.39	1.64	13876
9	2011	4	-1.82	0.95	0.06	-3.70	0.06	13839
10	2014	4	-0.93	0.65	0.16	-2.22	0.36	13780
11	2017	4	-0.67	1.22	0.58	-3.07	1.74	13704
12	2020	4	0.53	0.86	0.53	-1.16	2.23	13597
13	2017	5	0.31	0.71	0.67	-1.10	1.71	11225
14	2020	5	1.58	0.92	0.09	-0.24	3.40	11147

5 Death Over Time: Full Regression Results

The tables below present the results from Figure 4 in the text, but with full statistical reporting.

Supplementary Table 6: All Caused Deaths

	Year	Wave	Coef	SE	Pvalue	LB	UB	N
1	2002-04	3	-0.17	0.21	0.43	-0.59	0.25	12959
2	2005-07	3	-0.13	0.52	0.81	-1.15	0.89	12928
3	2008-10	3	-0.81	0.42	0.06	-1.65	0.03	12897
4	2011-13	3	0.82	0.33	0.01	0.17	1.48	12858
5	2014-16	3	-0.24	0.41	0.56	-1.04	0.56	12815
6	2017-19	3	0.44	0.48	0.37	-0.52	1.39	12763
7	2020-22	3	1.58	0.63	0.01	0.32	2.83	12680
8	2008-10	4	-0.07	0.25	0.78	-0.56	0.42	13892
9	2011-13	4	-0.38	0.32	0.23	-1.01	0.25	13858
10	2014-16	4	-0.19	0.31	0.54	-0.81	0.42	13811
11	2017-19	4	0.47	0.55	0.39	-0.61	1.55	13754
12	2020-22	4	0.86	0.47	0.07	-0.07	1.80	13665
13	2017-19	5	0.24	0.40	0.54	-0.55	1.04	11250
14	2020-22	5	1.35	0.54	0.01	0.28	2.41	11204

Supplementary Table 7: Internally Caused Deaths

	Year	Wave	Coef	SE	Pvalue	LB	UB	N
1	2002-04	3	-0.12	0.11	0.30	-0.34	0.11	12934
2	2005-07	3	-0.06	0.08	0.45	-0.21	0.09	12904
3	2008-10	3	-0.08	0.24	0.73	-0.55	0.39	12878
4	2011-13	3	0.51	0.28	0.08	-0.06	1.07	12834
5	2014-16	3	0.22	0.23	0.35	-0.24	0.69	12793
6	2017-19	3	0.18	0.42	0.67	-0.65	1.01	12727
7	2020-22	3	1.21	0.59	0.04	0.04	2.38	12646
8	2008-10	4	0.08	0.11	0.45	-0.14	0.31	13874
9	2011-13	4	0.09	0.20	0.67	-0.30	0.47	13830
10	2014-16	4	0.02	0.27	0.93	-0.51	0.55	13785
11	2017-19	4	0.70	0.29	0.02	0.12	1.28	13715
12	2020-22	4	0.67	0.33	0.04	0.02	1.32	13625
13	2017-19	5	0.18	0.36	0.62	-0.54	0.90	11229
14	2020-22	5	0.92	0.41	0.03	0.11	1.73	11183

Supplementary Table 8: Externally Caused Deaths

	Year	Wave	Coef	SE	Pvalue	LB	UB	N
1	2002-04	3	-0.05	0.18	0.77	-0.41	0.31	12953
2	2005-07	3	-0.07	0.51	0.89	-1.08	0.94	12921
3	2008-10	3	-0.73	0.35	0.04	-1.43	-0.03	12877
4	2011-13	3	0.32	0.17	0.06	-0.01	0.65	12839
5	2014-16	3	-0.46	0.33	0.16	-1.11	0.19	12785
6	2017-19	3	0.26	0.38	0.50	-0.50	1.02	12716
7	2020-22	3	0.38	0.23	0.10	-0.08	0.84	12615
8	2008-10	4	-0.15	0.22	0.49	-0.60	0.29	13876
9	2011-13	4	-0.47	0.25	0.06	-0.96	0.03	13839
10	2014-16	4	-0.22	0.16	0.18	-0.53	0.10	13780
11	2017-19	4	-0.23	0.42	0.59	-1.06	0.61	13704
12	2020-22	4	0.20	0.32	0.53	-0.43	0.83	13597
13	2017-19	5	0.07	0.15	0.66	-0.23	0.36	11225
14	2020-22	5	0.44	0.31	0.16	-0.17	1.04	11147

6 Internally Caused Death, Categorical Birth Year

This section replicates the results of table 2 in the main text using categorical indicators for birth year groups rather than a continuous variable for age.

Supplementary Table 9: Internally Caused Death, Categorical Birth Year

	(1) Internal	(2) Internal: Non-Covid	(3) Covid	(4) Internal	(5) Internal
Liberal	0.402	0.377	0.025	0.570	0.493
SE	(0.214)	(0.214)	(0.025)	(0.238)	(0.271)
Pvalue	0.063	0.080	0.318	0.018	0.071
95% CI	[-0.02,0.83]	[-0.05,0.80]	[-0.02,0.07]	[0.10,1.04]	[-0.04,1.03]
Moderate	0.747	0.680	0.068	0.642	0.500
SE	(0.191)	(0.182)	(0.039)	(0.181)	(0.234)
Pvalue	0.000	0.000	0.084	0.001	0.034
95% CI	[0.37,1.12]	[0.32,1.04]	[-0.01,0.15]	[0.28,1.00]	[0.04,0.96]
Conservative	0.745	0.468	0.279	0.636	0.642
SE	(0.278)	(0.200)	(0.155)	(0.293)	(0.368)
Pvalue	0.008	0.021	0.073	0.032	0.084
95% CI	[0.19,1.30]	[0.07,0.86]	[-0.03,0.59]	[0.06,1.21]	[-0.09,1.37]
Very Conservative	1.144	0.807	0.343	1.066	1.441
SE	(0.489)	(0.423)	(0.258)	(0.528)	(0.683)
Pvalue	0.021	0.059	0.186	0.045	0.037
95% CI	[0.18,2.11]	[-0.03,1.64]	[-0.17,0.85]	[0.02,2.11]	[0.09,2.79]
Black				0.548	0.879
SE				(0.556)	(0.910)
Pvalue				0.326	0.336
95% CI				[-0.55,1.65]	[-0.92,2.68]
Hispanic				-0.563	-0.280
SE				(0.252)	(0.392)
Pvalue				0.027	0.477
95% CI				[-1.06,-0.06]	[-1.06,0.50]
Other Race				-0.501	-0.699
SE				(0.208)	(0.328)
Pvalue				0.018	0.035
95% CI				[-0.91,-0.09]	[-1.35,-0.05]
Male				-0.236	-0.270
SE				(0.260)	(0.316)
Pvalue				0.365	0.394
95% CI				[-0.75,0.28]	[-0.89,0.35]
BA+				-0.400	-0.243
SE				(0.205)	(0.300)
Pvalue				0.053	0.419
95% CI				[-0.81,0.01]	[-0.84,0.35]
Inc≥100k				-1.435	-1.186
SE				(0.366)	(0.520)
Pvalue				0.000	0.024
95% CI				[-2.16,-0.71]	[-2.21,-0.16]
30k< Inc <100k				-1.088	-0.907
SE				(0.388)	(0.506)
Pvalue				0.006	0.075
95% CI				[-1.86,-0.32]	[-1.91,0.09]
Hlth Ins				0.407	0.196
SE				(0.416)	(0.499)
Pvalue				0.330	0.695
95% CI				[-0.42,1.23]	[-0.79,1.18]
b. 1978-1979				-0.562	-0.682
SE				(0.355)	(0.420)
Pvalue				0.115	0.107
95% CI				[-1.26,0.14]	[-1.51,0.15]
b. 1980-1983				-0.703	-0.796
SE				(0.382)	(0.455)
Pvalue				0.068	0.083
95% CI				[-1.46,0.05]	[-1.70,0.10]
Rural				-0.407	-0.334
SE				(0.341)	(0.714)
Pvalue				0.234	0.640
95% CI				[-1.08,0.27]	[-1.75,1.08]
County Hlth Quartile				0.155	
SE				(0.133)	
Pvalue				0.246	
95% CI				[-0.11,0.42]	
Constant	0.002	0.002	0.000	1.181	
SE	(0.002)	(0.002)	(0.000)	(0.596)	
Pvalue	0.323	0.323	1.000	0.050	
95% CI	[-0.00,0.01]	[-0.00,0.01]	[-0.00,0.00]	[0.00,2.36]	
N	11,183	11,171	11,138	10,278	10,278
FE	No	No	No	No	County

Replicates Table 1 of the manuscript, but includes categorical variables for year of birth, rather than continuous year. Reference group is those born between 1974-1977.

7 Predictors of Death, 2020-22

This section presents analyses of mortality in 2020-2022 using various measures of ideology, samples, and weights. Because ideology, demographic, and health variables are missing for some portion of the sample in each wave, choices about which respondents and measures to analyze can produce slightly different results. Supplementary Table 10 includes only respondents for which ideology and all available covariates were available in wave 5. Supplementary Table 11 imputes demographics and ideology based on wave 4 responses where wave 5 was not available and is weighted to the wave 4 benchmarks; Supplementary Table 12 restricts all models to respondents for which all covariates were available. Supplementary Table 13 weights to wave 5 rather than wave 4 benchmarks. Supplementary Table 14 relies on wave 4 ideology only, while Supplementary Table 15 does the same but includes only respondents for whom all covariates were available.

Supplementary Table 10: Wave 5 ideology, subsample with all available covariates

	(1) Internal	(2) Internal: Non-Covid	(3) Covid	(4) Internal	(5) Internal
Liberal	0.444	0.417	0.027	0.571	0.492
SE	(0.235)	(0.235)	(0.027)	(0.236)	(0.268)
Pvalue	0.061	0.078	0.318	0.017	0.069
95% CI	[-0.02,0.91]	[-0.05,0.88]	[-0.03,0.08]	[0.10,1.04]	[-0.04,1.02]
Moderate	0.757	0.684	0.075	0.648	0.507
SE	(0.204)	(0.194)	(0.043)	(0.182)	(0.234)
Pvalue	0.000	0.001	0.085	0.001	0.032
95% CI	[0.35,1.16]	[0.30,1.07]	[-0.01,0.16]	[0.29,1.01]	[0.04,0.97]
Conservative	0.611	0.309	0.304	0.635	0.647
SE	(0.250)	(0.185)	(0.168)	(0.296)	(0.372)
Pvalue	0.016	0.097	0.073	0.034	0.084
95% CI	[0.12,1.11]	[-0.06,0.68]	[-0.03,0.64]	[0.05,1.22]	[-0.09,1.38]
Very Conservative	1.168	0.794	0.380	1.061	1.440
SE	(0.536)	(0.462)	(0.285)	(0.529)	(0.685)
Pvalue	0.031	0.088	0.185	0.047	0.037
95% CI	[0.11,2.23]	[-0.12,1.71]	[-0.18,0.94]	[0.01,2.11]	[0.08,2.80]
Black				0.545	0.873
SE				(0.555)	(0.905)
Pvalue				0.327	0.337
95% CI				[-0.55,1.64]	[-0.92,2.66]
Hispanic				-0.559	-0.276
SE				(0.253)	(0.393)
Pvalue				0.029	0.484
95% CI				[-1.06,-0.06]	[-1.05,0.50]
Other Race				-0.497	-0.693
SE				(0.210)	(0.330)
Pvalue				0.019	0.038
95% CI				[-0.91,-0.08]	[-1.35,-0.04]
Male				-0.232	-0.261
SE				(0.260)	(0.316)
Pvalue				0.374	0.410
95% CI				[-0.75,0.28]	[-0.89,0.36]
BA+				-0.395	-0.243
SE				(0.206)	(0.301)
Pvalue				0.058	0.422
95% CI				[-0.80,0.01]	[-0.84,0.35]
Inc≥100k				-1.442	-1.198
SE				(0.367)	(0.521)
Pvalue				0.000	0.023
95% CI				[-2.17,-0.71]	[-2.23,-0.17]
30k< Inc <100k				-1.094	-0.913
SE				(0.390)	(0.507)
Pvalue				0.006	0.074
95% CI				[-1.86,-0.32]	[-1.92,0.09]
Hlth Ins				0.414	0.204
SE				(0.415)	(0.498)
Pvalue				0.321	0.683
95% CI				[-0.41,1.23]	[-0.78,1.19]
Birth Year				-0.139	-0.143
SE				(0.091)	(0.108)
Pvalue				0.127	0.188
95% CI				[-0.32,0.04]	[-0.36,0.07]
Rural				-0.410	-0.330
SE				(0.342)	(0.715)
Pvalue				0.233	0.645
95% CI				[-1.09,0.27]	[-1.75,1.08]
County Hlth Quartile				0.155	
SE				(0.134)	
Pvalue				0.249	
95% CI				[-0.11,0.42]	
Constant	0.000	0.000	0.000	276.593	
SE	(0.000)	(0.000)	(0.000)	(179.616)	
Pvalue	1.000	1.000	1.000	0.126	
95% CI	[-0.00,0.00]	[-0.00,0.00]	[-0.00,0.00]	[-78.81,631.99]	
N	10,278	10,266	10,242	10,278	10,278
FE	No	No	No	No	County

Deaths measured by wave 5 reported ideology. This model include only respondents for which available covariates are available.

Supplementary Table 11: Wave 4/5 ideology, all

	(1) Internal	(2) Internal: Non-Covid	(3) Covid	(4) Internal	(5) Internal
Liberal	0.056	0.019	0.037	0.095	0.139
SE	(0.133)	(0.127)	(0.037)	(0.139)	(0.153)
Pvalue	0.675	0.884	0.317	0.495	0.362
95% CI	[-0.21,0.32]	[-0.23,0.27]	[-0.04,0.11]	[-0.18,0.37]	[-0.16,0.44]
Moderate	0.580	0.536	0.045	0.464	0.388
SE	(0.194)	(0.190)	(0.026)	(0.191)	(0.226)
Pvalue	0.003	0.005	0.088	0.017	0.089
95% CI	[0.20,0.96]	[0.16,0.91]	[-0.01,0.10]	[0.09,0.84]	[-0.06,0.84]
Conservative	0.541	0.371	0.172	0.489	0.499
SE	(0.228)	(0.206)	(0.100)	(0.253)	(0.295)
Pvalue	0.019	0.074	0.089	0.055	0.093
95% CI	[0.09,0.99]	[-0.04,0.78]	[-0.03,0.37]	[-0.01,0.99]	[-0.08,1.08]
Very Conservative	0.777	0.642	0.138	0.622	0.778
SE	(0.411)	(0.393)	(0.137)	(0.413)	(0.495)
Pvalue	0.061	0.105	0.317	0.134	0.119
95% CI	[-0.04,1.59]	[-0.14,1.42]	[-0.13,0.41]	[-0.19,1.44]	[-0.20,1.76]
Black				0.015	0.057
SE				(0.365)	(0.567)
Pvalue				0.966	0.919
95% CI				[-0.71,0.74]	[-1.06,1.18]
Hispanic				-0.519	-0.310
SE				(0.217)	(0.333)
Pvalue				0.018	0.355
95% CI				[-0.95,-0.09]	[-0.97,0.35]
Other Race				-0.288	-0.497
SE				(0.205)	(0.283)
Pvalue				0.163	0.082
95% CI				[-0.69,0.12]	[-1.06,0.06]
Male				-0.220	-0.250
SE				(0.201)	(0.228)
Pvalue				0.274	0.275
95% CI				[-0.62,0.18]	[-0.70,0.20]
BA+				-0.327	-0.216
SE				(0.154)	(0.213)
Pvalue				0.036	0.311
95% CI				[-0.63,-0.02]	[-0.64,0.20]
Inc$\geq$100k				-1.080	-0.852
SE				(0.341)	(0.450)
Pvalue				0.002	0.061
95% CI				[-1.75,-0.41]	[-1.74,0.04]
30k$<$ Inc $<$100k				-0.885	-0.722
SE				(0.347)	(0.417)
Pvalue				0.012	0.086
95% CI				[-1.57,-0.20]	[-1.55,0.10]
Hlth Ins				0.027	0.018
SE				(0.059)	(0.056)
Pvalue				0.646	0.750
95% CI				[-0.09,0.14]	[-0.09,0.13]
Birth Year				-0.116	-0.116
SE				(0.061)	(0.074)
Pvalue				0.062	0.118
95% CI				[-0.24,0.01]	[-0.26,0.03]
Rural				-0.051	-0.059
SE				(0.283)	(0.661)
Pvalue				0.857	0.929
95% CI				[-0.61,0.51]	[-1.37,1.25]
County Hlth Quartile				0.167	0.121
SE				(0.107)	(2.057)
Pvalue				0.123	0.953
95% CI				[-0.05,0.38]	[-3.95,4.19]
Constant	0.130	0.130	-0.000	229.747	
SE	(0.114)	(0.114)	(.)	(121.409)	
Pvalue	0.255	0.255	.	0.061	
95% CI	[-0.10,0.35]	[-0.10,0.35]	[.,.]	[-10.48,469.97]	
N	14,053	14,040	13,994	13,703	13,703
FE	No	No	No	No	County

Deaths measured by wave 5 reported ideology and covariates, if available. For those which wave 5 data are not available, we include wave 4 measures, if available. Responses are weighted to wave 4 cross-section.

Supplementary Table 12: Wave 4/5 ideology, subsample

	(1) Internal	(2) Internal: Non-Covid	(3) Covid	(4) Internal	(5) Internal
Liberal	0.057	0.018	0.038	0.095	0.139
SE	(0.137)	(0.131)	(0.038)	(0.139)	(0.153)
Pvalue	0.680	0.890	0.317	0.495	0.362
95% CI	[-0.21,0.33]	[-0.24,0.28]	[-0.04,0.11]	[-0.18,0.37]	[-0.16,0.44]
Moderate	0.594	0.548	0.046	0.464	0.388
SE	(0.199)	(0.195)	(0.027)	(0.191)	(0.226)
Pvalue	0.003	0.006	0.088	0.017	0.089
95% CI	[0.20,0.99]	[0.16,0.93]	[-0.01,0.10]	[0.09,0.84]	[-0.06,0.84]
Conservative	0.553	0.379	0.176	0.489	0.499
SE	(0.234)	(0.212)	(0.103)	(0.253)	(0.295)
Pvalue	0.020	0.076	0.089	0.055	0.093
95% CI	[0.09,1.02]	[-0.04,0.80]	[-0.03,0.38]	[-0.01,0.99]	[-0.08,1.08]
Very Conservative	0.792	0.654	0.140	0.622	0.778
SE	(0.420)	(0.402)	(0.140)	(0.413)	(0.495)
Pvalue	0.061	0.106	0.317	0.134	0.119
95% CI	[-0.04,1.62]	[-0.14,1.45]	[-0.14,0.42]	[-0.19,1.44]	[-0.20,1.76]
Black				0.015	0.057
SE				(0.365)	(0.567)
Pvalue				0.966	0.919
95% CI				[-0.71,0.74]	[-1.06,1.18]
Hispanic				-0.519	-0.310
SE				(0.217)	(0.333)
Pvalue				0.018	0.355
95% CI				[-0.95,-0.09]	[-0.97,0.35]
Other Race				-0.288	-0.497
SE				(0.205)	(0.283)
Pvalue				0.163	0.082
95% CI				[-0.69,0.12]	[-1.06,0.06]
Male				-0.220	-0.250
SE				(0.201)	(0.228)
Pvalue				0.274	0.275
95% CI				[-0.62,0.18]	[-0.70,0.20]
BA+				-0.327	-0.216
SE				(0.154)	(0.213)
Pvalue				0.036	0.311
95% CI				[-0.63,-0.02]	[-0.64,0.20]
Inc≥100k				-1.080	-0.852
SE				(0.341)	(0.450)
Pvalue				0.002	0.061
95% CI				[-1.75,-0.41]	[-1.74,0.04]
30k< Inc <100k				-0.885	-0.722
SE				(0.347)	(0.417)
Pvalue				0.012	0.086
95% CI				[-1.57,-0.20]	[-1.55,0.10]
Hlth Ins				0.027	0.018
SE				(0.059)	(0.056)
Pvalue				0.646	0.750
95% CI				[-0.09,0.14]	[-0.09,0.13]
Birth Year				-0.116	-0.116
SE				(0.061)	(0.074)
Pvalue				0.062	0.118
95% CI				[-0.24,0.01]	[-0.26,0.03]
Rural				-0.051	-0.059
SE				(0.283)	(0.661)
Pvalue				0.857	0.929
95% CI				[-0.61,0.51]	[-1.37,1.25]
County Hlth Quartile				0.167	0.121
SE				(0.107)	(2.057)
Pvalue				0.123	0.953
95% CI				[-0.05,0.38]	[-3.95,4.19]
Constant	0.134	0.134	0.000	229.747	
SE	(0.118)	(0.118)	(0.000)	(121.409)	
Pvalue	0.256	0.256	1.000	0.061	
95% CI	[-0.10,0.37]	[-0.10,0.37]	[-0.00,0.00]	[-10.48,469.97]	
N	13,703	13,690	13,644	13,703	13,703
FE	No	No	No	No	County

Deaths measured by wave 5 reported ideology and covariates, if available. For those which wave 5 data are not available, we include wave 4 measures, if available. Only respondents for which all covariates are available are included.

Supplementary Table 13: Wave 4/5 ideology, weighted to wave 5 cross-section

	(1) Internal	(2) Internal: Non-Covid	(3) Covid	(4) Internal	(5) Internal
Liberal	0.388	0.365	0.024	0.512	0.402
SE	(0.208)	(0.207)	(0.024)	(0.217)	(0.271)
Pvalue	0.064	0.081	0.318	0.020	0.140
95% CI	[-0.02,0.80]	[-0.05,0.78]	[-0.02,0.07]	[0.08,0.94]	[-0.13,0.94]
Moderate	0.748	0.684	0.065	0.662	0.542
SE	(0.182)	(0.174)	(0.037)	(0.167)	(0.189)
Pvalue	0.000	0.000	0.085	0.000	0.005
95% CI	[0.39,1.11]	[0.34,1.03]	[-0.01,0.14]	[0.33,0.99]	[0.17,0.92]
Conservative	0.721	0.453	0.270	0.656	0.582
SE	(0.269)	(0.194)	(0.150)	(0.275)	(0.298)
Pvalue	0.008	0.021	0.073	0.019	0.053
95% CI	[0.19,1.25]	[0.07,0.84]	[-0.03,0.57]	[0.11,1.20]	[-0.01,1.17]
Very Conservative	1.103	0.777	0.331	1.008	1.290
SE	(0.470)	(0.406)	(0.249)	(0.474)	(0.627)
Pvalue	0.021	0.058	0.186	0.035	0.042
95% CI	[0.17,2.03]	[-0.03,1.58]	[-0.16,0.82]	[0.07,1.95]	[0.05,2.53]
Black				0.458	0.692
SE				(0.503)	(0.832)
Pvalue				0.364	0.408
95% CI				[-0.54,1.45]	[-0.96,2.34]
Hispanic				-0.570	-0.301
SE				(0.234)	(0.417)
Pvalue				0.016	0.472
95% CI				[-1.03,-0.11]	[-1.13,0.52]
Other Race				-0.530	-0.746
SE				(0.196)	(0.315)
Pvalue				0.008	0.019
95% CI				[-0.92,-0.14]	[-1.37,-0.12]
Male				-0.250	-0.276
SE				(0.246)	(0.285)
Pvalue				0.310	0.334
95% CI				[-0.74,0.24]	[-0.84,0.29]
BA+				-0.305	-0.143
SE				(0.196)	(0.289)
Pvalue				0.122	0.622
95% CI				[-0.69,0.08]	[-0.71,0.43]
Inc≥100k				-1.461	-1.296
SE				(0.330)	(0.450)
Pvalue				0.000	0.005
95% CI				[-2.11,-0.81]	[-2.19,-0.41]
30k< Inc <100k				-1.113	-0.990
SE				(0.348)	(0.440)
Pvalue				0.002	0.026
95% CI				[-1.80,-0.43]	[-1.86,-0.12]
Hlth Ins				0.323	0.274
SE				(0.381)	(0.512)
Pvalue				0.398	0.593
95% CI				[-0.43,1.08]	[-0.74,1.29]
Birth Year				-0.146	-0.152
SE				(0.082)	(0.098)
Pvalue				0.077	0.123
95% CI				[-0.31,0.02]	[-0.34,0.04]
Rural				-0.240	-0.201
SE				(0.324)	(0.718)
Pvalue				0.460	0.780
95% CI				[-0.88,0.40]	[-1.62,1.22]
County Hlth Quartile				0.101	0.598
SE				(0.122)	(8.558)
Pvalue				0.409	0.944
95% CI				[-0.14,0.34]	[-16.33,17.53]
Constant	0.002	0.002	0.000	289.887	299.496
SE	(0.002)	(0.002)	(.)	(162.197)	(198.121)
Pvalue	0.323	0.323	.	0.076	0.133
95% CI	[-0.00,0.01]	[-0.00,0.01]	[.,.]	[-31.05,610.82]	[-92.52,691.51]
N	11,739	11,727	11,692	11,536	11,536
FE	No	No	No	No	County

Deaths measured by wave 5 reported ideology and covariates, if available. For those which wave 5 data are not available, we include wave 4 measures, if available. Responses are weighted to wave 5 cross-section.

Supplementary Table 14: Wave 4 ideology, all

	(1) Internal	(2) Internal: Non-Covid	(3) Covid	(4) Internal	(5) Internal
Liberal	-0.095	-0.095	-0.000	-0.075	-0.234
SE	(0.216)	(0.216)	(.)	(0.206)	(0.233)
Pvalue	0.662	0.662	.	0.717	0.318
95% CI	[-0.52,0.33]	[-0.52,0.33]	[.,.]	[-0.48,0.33]	[-0.70,0.23]
Moderate	0.348	0.246	0.103	0.334	0.256
SE	(0.223)	(0.216)	(0.052)	(0.229)	(0.270)
Pvalue	0.121	0.257	0.048	0.147	0.345
95% CI	[-0.09,0.79]	[-0.18,0.67]	[0.00,0.21]	[-0.12,0.79]	[-0.28,0.79]
Conservative	0.210	0.181	0.030	0.258	0.215
SE	(0.288)	(0.287)	(0.028)	(0.313)	(0.356)
Pvalue	0.467	0.529	0.293	0.411	0.547
95% CI	[-0.36,0.78]	[-0.39,0.75]	[-0.03,0.09]	[-0.36,0.88]	[-0.49,0.92]
Very Conservative	0.792	0.291	0.507	0.774	0.710
SE	(0.521)	(0.421)	(0.318)	(0.540)	(0.626)
Pvalue	0.131	0.491	0.113	0.154	0.259
95% CI	[-0.24,1.82]	[-0.54,1.12]	[-0.12,1.14]	[-0.29,1.84]	[-0.53,1.95]
Black				0.223	0.106
SE				(0.350)	(0.542)
Pvalue				0.525	0.845
95% CI				[-0.47,0.92]	[-0.97,1.18]
Hispanic				-0.427	-0.359
SE				(0.227)	(0.340)
Pvalue				0.062	0.293
95% CI				[-0.88,0.02]	[-1.03,0.31]
Other Race				-0.301	-0.283
SE				(0.197)	(0.266)
Pvalue				0.130	0.289
95% CI				[-0.69,0.09]	[-0.81,0.24]
Male				-0.202	-0.182
SE				(0.209)	(0.241)
Pvalue				0.336	0.451
95% CI				[-0.61,0.21]	[-0.66,0.29]
BA+				-0.446	-0.426
SE				(0.179)	(0.246)
Pvalue				0.014	0.085
95% CI				[-0.80,-0.09]	[-0.91,0.06]
Inc≥100k				-0.363	-0.123
SE				(0.359)	(0.428)
Pvalue				0.313	0.775
95% CI				[-1.07,0.35]	[-0.97,0.72]
30k< Inc <100k				-0.379	-0.256
SE				(0.328)	(0.385)
Pvalue				0.250	0.508
95% CI				[-1.03,0.27]	[-1.02,0.51]
Hlth Ins				-0.073	-0.040
SE				(0.080)	(0.071)
Pvalue				0.359	0.574
95% CI				[-0.23,0.08]	[-0.18,0.10]
Birth Year				-0.138	-0.144
SE				(0.065)	(0.077)
Pvalue				0.037	0.063
95% CI				[-0.27,-0.01]	[-0.30,0.01]
Rural				-0.023	
SE				(0.239)	
Pvalue				0.925	
95% CI				[-0.49,0.45]	
County Hlth Quartile				0.127	
SE				(0.101)	
Pvalue				0.211	
95% CI				[-0.07,0.33]	
Constant	0.340	0.340	0.000	273.636	
SE	(0.190)	(0.190)	(.)	(129.641)	
Pvalue	0.075	0.075	.	0.037	
95% CI	[-0.03,0.72]	[-0.03,0.72]	[.,.]	[17.12,530.15]	
N	13,625	13,612	13,570	12,793	12,792
FE	No	No	No	No	County

Deaths measured by wave 4 reported ideology. All available respondents in each model.

Supplementary Table 15: Wave 4 ideology, subsample with all available covariates

	(1) Internal	(2) Internal: Non-Covid	(3) Covid	(4) Internal	(5) Internal
Liberal	-0.090	-0.090	-0.000	-0.075	-0.234
SE	(0.201)	(0.201)	(.)	(0.206)	(0.233)
Pvalue	0.656	0.656	.	0.717	0.318
95% CI	[-0.49,0.31]	[-0.49,0.31]	[.,.]	[-0.48,0.33]	[-0.70,0.23]
Moderate	0.406	0.297	0.110	0.334	0.256
SE	(0.228)	(0.219)	(0.055)	(0.229)	(0.270)
Pvalue	0.077	0.178	0.048	0.147	0.345
95% CI	[-0.04,0.86]	[-0.14,0.73]	[0.00,0.22]	[-0.12,0.79]	[-0.28,0.79]
Conservative	0.308	0.276	0.032	0.258	0.215
SE	(0.294)	(0.293)	(0.030)	(0.313)	(0.356)
Pvalue	0.297	0.347	0.293	0.411	0.547
95% CI	[-0.27,0.89]	[-0.30,0.86]	[-0.03,0.09]	[-0.36,0.88]	[-0.49,0.92]
Very Conservative	0.897	0.415	0.488	0.774	0.710
SE	(0.555)	(0.448)	(0.343)	(0.540)	(0.626)
Pvalue	0.109	0.355	0.157	0.154	0.259
95% CI	[-0.20,1.99]	[-0.47,1.30]	[-0.19,1.17]	[-0.29,1.84]	[-0.53,1.95]
Black				0.223	0.106
SE				(0.350)	(0.542)
Pvalue				0.525	0.845
95% CI				[-0.47,0.92]	[-0.97,1.18]
Hispanic				-0.428	-0.359
SE				(0.227)	(0.340)
Pvalue				0.061	0.293
95% CI				[-0.88,0.02]	[-1.03,0.31]
Other Race				-0.301	-0.283
SE				(0.197)	(0.266)
Pvalue				0.130	0.289
95% CI				[-0.69,0.09]	[-0.81,0.24]
Male				-0.202	-0.182
SE				(0.209)	(0.241)
Pvalue				0.335	0.451
95% CI				[-0.61,0.21]	[-0.66,0.29]
BA+				-0.447	-0.426
SE				(0.179)	(0.246)
Pvalue				0.014	0.085
95% CI				[-0.80,-0.09]	[-0.91,0.06]
Inc$\geq$100k				-0.363	-0.123
SE				(0.359)	(0.428)
Pvalue				0.313	0.775
95% CI				[-1.07,0.35]	[-0.97,0.72]
30k$<$ Inc $<$100k				-0.379	-0.256
SE				(0.328)	(0.385)
Pvalue				0.250	0.508
95% CI				[-1.03,0.27]	[-1.02,0.51]
Hlth Ins				-0.073	-0.040
SE				(0.080)	(0.071)
Pvalue				0.359	0.574
95% CI				[-0.23,0.08]	[-0.18,0.10]
Birth Year				-0.138	-0.144
SE				(0.065)	(0.077)
Pvalue				0.037	0.063
95% CI				[-0.27,-0.01]	[-0.30,0.01]
Rural				-0.023	0.000
SE				(0.239)	(.)
Pvalue				0.925	.
95% CI				[-0.49,0.45]	[0.00,0.00]
County Hlth Quartile				0.127	0.000
SE				(0.101)	(.)
Pvalue				0.211	.
95% CI				[-0.07,0.33]	[0.00,0.00]
Constant	0.283	0.283	0.000	273.640	286.477
SE	(0.184)	(0.184)	(0.000)	(129.640)	(152.244)
Pvalue	0.127	0.127	1.000	0.037	0.062
95% CI	[-0.08,0.65]	[-0.08,0.65]	[-0.00,0.00]	[17.12,530.15]	[-14.76,587.72]
N	12,792	12,780	12,741	12,792	12,792
FE	No	No	No	No	County

Deaths measured by wave 4 reported ideology. This model include only respondents for which available covariates are available.

8 Multivariable Analysis: Trust and Engagement

This section presents the full results of multivariate models of trust in and engagement with the medical system, as predicted by vote choice (16 and 17), partisanship (18 and 19), and ideology (20 and 21). Each model is run on the full available sample, as well as the subset who reported diagnosis with a chronic medical condition (indicated in column titles with "DX").

Supplementary Table 16: Multi-Variable Analysis: Vote Choice

	Primary Care Provider						Emergency Room Doctor					
	(1) Trust (All)	(2) Trust (All)	(3) Trust (DX)	(4) Advice (All)	(5) Advice (All)	(6) Advice (DX)	(7) Trust (All)	(8) Trust (All)	(9) Trust (DX)	(10) Advice (All)	(11) Advice (All)	(12) Advice (DX)
Vote Trump	-0.034	-0.031	-0.027	-0.032	-0.029	-0.026	-0.062	-0.047	-0.052	-0.066	-0.045	-0.036
SE	(0.00)	(0.00)	(0.01)	(0.00)	(0.00)	(0.01)	(0.01)	(0.01)	(0.01)	(0.01)	(0.01)	(0.02)
Pvalue	0.000	0.000	0.000	0.000	0.000	0.000	0.000	0.000	0.000	0.000	0.000	0.020
95% CI	[-0.04,-0.03]	[-0.04,-0.02]	[-0.04,-0.01]	[-0.04,-0.02]	[-0.04,-0.02]	[-0.04,-0.01]	[-0.08,-0.04]	[-0.07,-0.03]	[-0.08,-0.02]	[-0.09,-0.05]	[-0.07,-0.02]	[-0.07,-0.01]
BA+		0.008	0.018		0.007	0.008		0.009	0.005		0.025	0.005
SE		(0.00)	(0.01)		(0.00)	(0.01)		(0.01)	(0.01)		(0.01)	(0.01)
Pvalue		0.066	0.003		0.121	0.198		0.402	0.703		0.013	0.758
95% CI		[-0.00,0.02]	[0.01,0.03]		[-0.00,0.02]	[-0.00,0.02]		[-0.01,0.03]	[-0.02,0.03]		[0.01,0.04]	[-0.02,0.03]
Black		-0.004	-0.006		-0.010	-0.021		-0.005	-0.017		0.018	-0.008
SE		(0.01)	(0.01)		(0.01)	(0.01)		(0.02)	(0.02)		(0.02)	(0.03)
Pvalue		0.478	0.499		0.167	0.049		0.766	0.447		0.229	0.764
95% CI		[-0.02,0.01]	[-0.02,0.01]		[-0.02,0.00]	[-0.04,-0.00]		[-0.03,0.03]	[-0.06,0.03]		[-0.01,0.05]	[-0.06,0.04]
Hispanic		-0.004	-0.008		-0.011	-0.002		-0.009	-0.047		-0.019	-0.034
SE		(0.01)	(0.01)		(0.01)	(0.01)		(0.02)	(0.03)		(0.02)	(0.03)
Pvalue		0.655	0.601		0.221	0.890		0.642	0.108		0.294	0.237
95% CI		[-0.02,0.01]	[-0.04,0.02]		[-0.03,0.01]	[-0.03,0.03]		[-0.05,0.03]	[-0.11,0.01]		[-0.06,0.02]	[-0.09,0.02]
Other Race		-0.022	-0.039		-0.034	-0.052		-0.028	-0.071		-0.065	-0.106
SE		(0.01)	(0.01)		(0.01)	(0.01)		(0.02)	(0.03)		(0.02)	(0.04)
Pvalue		0.007	0.008		0.000	0.000		0.210	0.037		0.007	0.007
95% CI		[-0.04,-0.01]	[-0.07,-0.01]		[-0.05,-0.02]	[-0.08,-0.02]		[-0.07,0.02]	[-0.14,-0.00]		[-0.11,-0.02]	[-0.18,-0.03]
Age		0.001	0.002		0.001	0.002		0.002	0.002		0.002	0.002
SE		(0.00)	(0.00)		(0.00)	(0.00)		(0.00)	(0.00)		(0.00)	(0.00)
Pvalue		0.000	0.000		0.000	0.000		0.000	0.000		0.000	0.000
95% CI		[0.00,0.00]	[0.00,0.00]		[0.00,0.00]	[0.00,0.00]		[0.00,0.00]	[0.00,0.00]		[0.00,0.00]	[0.00,0.00]
Male		0.012	0.016		0.000	0.006		0.027	0.042		0.001	0.016
SE		(0.00)	(0.01)		(0.00)	(0.01)		(0.01)	(0.01)		(0.01)	(0.01)
Pvalue		0.007	0.012		0.949	0.395		0.008	0.003		0.887	0.257
95% CI		[0.00,0.02]	[0.00,0.03]		[-0.01,0.01]	[-0.01,0.02]		[0.01,0.05]	[0.01,0.07]		[-0.02,0.02]	[-0.01,0.04]
Hlth Ins		0.059	0.059		0.063	0.063		0.028	0.078		0.061	0.065
SE		(0.01)	(0.02)		(0.01)	(0.02)		(0.02)	(0.03)		(0.02)	(0.03)
Pvalue		0.000	0.002		0.000	0.000		0.095	0.008		0.000	0.012
95% CI		[0.04,0.08]	[0.02,0.10]		[0.04,0.08]	[0.03,0.10]		[-0.00,0.06]	[0.02,0.14]		[0.03,0.10]	[0.01,0.12]
Inc $\geq$ 100K		0.026	0.024		0.032	0.042		0.034	0.025		0.076	0.077
SE		(0.01)	(0.01)		(0.01)	(0.01)		(0.02)	(0.02)		(0.02)	(0.03)
Pvalue		0.000	0.015		0.000	0.000		0.052	0.319		0.000	0.002
95% CI		[0.01,0.04]	[0.00,0.04]		[0.02,0.05]	[0.02,0.06]		[-0.00,0.07]	[-0.02,0.07]		[0.04,0.11]	[0.03,0.13]
25K< Inc <100K		0.009	0.005		0.010	0.010		0.031	0.027		0.032	0.029
SE		(0.01)	(0.01)		(0.01)	(0.01)		(0.01)	(0.02)		(0.01)	(0.02)
Pvalue		0.139	0.591		0.126	0.257		0.022	0.162		0.013	0.129
95% CI		[-0.00,0.02]	[-0.01,0.02]		[-0.00,0.02]	[-0.01,0.03]		[0.00,0.06]	[-0.01,0.07]		[0.01,0.06]	[-0.01,0.07]
Rural		-0.001	-0.011		0.003	-0.003		-0.044	-0.022		-0.014	-0.004
SE		(0.01)	(0.01)		(0.01)	(0.01)		(0.01)	(0.02)		(0.01)	(0.02)
Pvalue		0.907	0.221		0.676	0.695		0.003	0.276		0.306	0.836
95% CI		[-0.01,0.01]	[-0.03,0.01]		[-0.01,0.02]	[-0.02,0.01]		[-0.07,-0.02]	[-0.06,0.02]		[-0.04,0.01]	[-0.04,0.03]
Constant	0.870	0.726	0.717	0.782	0.637	0.631	0.790	0.629	0.590	0.767	0.568	0.590
SE	(0.00)	(0.01)	(0.02)	(0.00)	(0.01)	(0.02)	(0.01)	(0.03)	(0.04)	(0.01)	(0.03)	(0.04)
Pvalue	0.000	0.000	0.000	0.000	0.000	0.000	0.000	0.000	0.000	0.000	0.000	0.000
95% CI	[0.86,0.88]	[0.70,0.75]	[0.67,0.76]	[0.78,0.79]	[0.61,0.66]	[0.59,0.68]	[0.78,0.80]	[0.58,0.68]	[0.51,0.67]	[0.75,0.78]	[0.52,0.62]	[0.52,0.66]
N	13464	13448	6219	13504	13488	6242	3500	3496	1689	3497	3493	1688

Note. Variable "Vote Trump" coded 1 if respondent indicated they would support Trump in 2024 election; 0 if Biden. Please note that each variable is reported with the regression coefficient, standard error (in parentheses), p-value and 95% confidence interval (in brackets).

Supplementary Table 17: Multi-Variable Analysis: Vote Choice

	(1) Chest Visit (All)	(2) Chest Visit (All)	(3) Chest Visit (DX)	(4) PCP Visit (All)	(5) PCP Visit (All)	(6) PCP Visit (DX)	(7) ER Visit (All)	(8) ER Visit (All)	(9) ER Visit (DX)	(10) BMQ (DX)	(11) BMQ (DX)
Vote Trump	-0.048	-0.028	-0.022	-0.060	-0.037	-0.020	0.034	0.020	0.028	-0.026	-0.026
SE	(0.01)	(0.01)	(0.01)	(0.01)	(0.01)	(0.01)	(0.01)	(0.01)	(0.01)	(0.01)	(0.01)
Pvalue	0.000	0.000	0.009	0.000	0.000	0.042	0.000	0.012	0.033	0.000	0.000
95% CI	[-0.06,-0.04]	[-0.04,-0.02]	[-0.04,-0.01]	[-0.07,-0.05]	[-0.05,-0.02]	[-0.04,-0.00]	[0.02,0.05]	[0.00,0.04]	[0.00,0.05]	[-0.04,-0.01]	[-0.04,-0.01]
BA+		0.024	0.021		0.022	0.005		-0.051	-0.048		-0.004
SE		(0.01)	(0.01)		(0.01)	(0.01)		(0.01)	(0.01)		(0.01)
Pvalue		0.000	0.012		0.003	0.562		0.000	0.000		0.439
95% CI		[0.01,0.03]	[0.00,0.04]		[0.01,0.04]	[-0.01,0.02]		[-0.07,-0.04]	[-0.07,-0.02]		[-0.02,0.01]
Black		0.060	0.042		0.026	0.017		0.007	-0.020		-0.014
SE		(0.01)	(0.01)		(0.01)	(0.01)		(0.01)	(0.02)		(0.01)
Pvalue		0.000	0.000		0.016	0.254		0.513	0.283		0.129
95% CI		[0.04,0.08]	[0.02,0.07]		[0.00,0.05]	[-0.01,0.05]		[-0.01,0.03]	[-0.06,0.02]		[-0.03,0.00]
Hispanic		0.025	0.008		-0.029	-0.048		0.008	0.048		-0.013
SE		(0.01)	(0.02)		(0.01)	(0.02)		(0.01)	(0.03)		(0.01)
Pvalue		0.014	0.656		0.045	0.037		0.595	0.076		0.288
95% CI		[0.01,0.04]	[-0.03,0.04]		[-0.06,-0.00]	[-0.09,-0.00]		[-0.02,0.04]	[-0.00,0.10]		[-0.04,0.01]
Other Race		0.003	-0.016		-0.001	-0.020		-0.080	-0.073		-0.047
SE		(0.01)	(0.02)		(0.01)	(0.02)		(0.01)	(0.03)		(0.01)
Pvalue		0.733	0.346		0.952	0.362		0.000	0.006		0.000
95% CI		[-0.02,0.02]	[-0.05,0.02]		[-0.03,0.03]	[-0.06,0.02]		[-0.11,-0.06]	[-0.12,-0.02]		[-0.07,-0.02]
Age		0.003	0.002		0.004	0.004		-0.001	-0.002		0.002
SE		(0.00)	(0.00)		(0.00)	(0.00)		(0.00)	(0.00)		(0.00)
Pvalue		0.000	0.000		0.000	0.000		0.000	0.000		0.000
95% CI		[0.00,0.00]	[0.00,0.00]		[0.00,0.00]	[0.00,0.00]		[-0.00,-0.00]	[-0.00,-0.00]		[0.00,0.00]
Male		0.028	0.038		-0.025	-0.019		-0.023	-0.025		-0.009
SE		(0.01)	(0.01)		(0.01)	(0.01)		(0.01)	(0.01)		(0.01)
Pvalue		0.000	0.000		0.001	0.041		0.003	0.050		0.123
95% CI		[0.02,0.04]	[0.02,0.05]		[-0.04,-0.01]	[-0.04,-0.00]		[-0.04,-0.01]	[-0.05,0.00]		[-0.02,0.00]
Hlth Ins		0.077	0.063		0.238	0.179		-0.010	-0.019		0.038
SE		(0.01)	(0.02)		(0.02)	(0.03)		(0.01)	(0.03)		(0.01)
Pvalue		0.000	0.000		0.000	0.000		0.472	0.484		0.001
95% CI		[0.06,0.10]	[0.03,0.10]		[0.21,0.27]	[0.13,0.23]		[-0.04,0.02]	[-0.07,0.03]		[0.02,0.06]
Inc $\geq$ 100K		0.020	0.015		0.041	0.055		-0.050	-0.056		-0.018
SE		(0.01)	(0.01)		(0.01)	(0.02)		(0.01)	(0.02)		(0.01)
Pvalue		0.026	0.252		0.001	0.001		0.000	0.009		0.061
95% CI		[0.00,0.04]	[-0.01,0.04]		[0.02,0.07]	[0.02,0.09]		[-0.08,-0.02]	[-0.10,-0.01]		[-0.04,0.00]
25K$<$ Inc $<$100K		0.005	-0.001		0.030	0.040		-0.025	-0.024		0.003
SE		(0.01)	(0.01)		(0.01)	(0.01)		(0.01)	(0.02)		(0.01)
Pvalue		0.530	0.957		0.003	0.002		0.019	0.148		0.679
95% CI		[-0.01,0.02]	[-0.02,0.02]		[0.01,0.05]	[0.01,0.07]		[-0.05,-0.00]	[-0.06,0.01]		[-0.01,0.02]
Rural		0.003	0.020		0.011	0.008		-0.004	-0.018		-0.005
SE		(0.01)	(0.01)		(0.01)	(0.01)		(0.01)	(0.02)		(0.01)
Pvalue		0.732	0.070		0.288	0.501		0.684	0.287		0.511
95% CI		[-0.01,0.02]	[-0.00,0.04]		[-0.01,0.03]	[-0.02,0.03]		[-0.03,0.02]	[-0.05,0.02]		[-0.02,0.01]
Constant	0.700	0.453	0.518	0.830	0.395	0.494	0.200	0.331	0.413	0.595	0.483
SE	(0.00)	(0.01)	(0.02)	(0.00)	(0.02)	(0.03)	(0.01)	(0.02)	(0.04)	(0.00)	(0.02)
Pvalue	0.000	0.000	0.000	0.000	0.000	0.000	0.000	0.000	0.000	0.000	0.000
95% CI	[0.69,0.71]	[0.43,0.48]	[0.47,0.56]	[0.82,0.84]	[0.35,0.44]	[0.43,0.56]	[0.19,0.21]	[0.29,0.37]	[0.34,0.49]	[0.59,0.60]	[0.45,0.52]
N	16347	16326	6935	16541	16519	6981	16541	16519	6981	4839	4835

Note. Variable "Vote Trump" coded 1 if respondent indicated they would support Trump in 2024 election; 0 if Biden. Please note that each variable is reported with the regression coefficient, standard error (in parentheses), p-value and 95% confidence interval (in brackets).

Supplementary Table 18: Multi-Variable Analysis: Partisanship

	Primary Care Provider						Emergency Room Doctor					
	(1) Trust (All)	(2) Trust (All)	(3) Trust (DX)	(4) Advice (All)	(5) Advice (All)	(6) Advice (DX)	(7) Trust (All)	(8) Trust (All)	(9) Trust (DX)	(10) Advice (All)	(11) Advice (All)	(12) Advice (DX)
Party ID	-0.028	-0.033	-0.025	-0.024	-0.030	-0.022	-0.031	-0.031	-0.032	-0.038	-0.031	-0.016
SE	(0.01)	(0.01)	(0.01)	(0.01)	(0.01)	(0.01)	(0.01)	(0.01)	(0.02)	(0.01)	(0.01)	(0.02)
Pvalue	0.000	0.000	0.002	0.000	0.000	0.007	0.014	0.017	0.074	0.001	0.014	0.387
95% CI	[-0.04,-0.02]	[-0.04,-0.02]	[-0.04,-0.01]	[-0.03,-0.01]	[-0.04,-0.02]	[-0.04,-0.01]	[-0.05,-0.01]	[-0.06,-0.01]	[-0.07,0.00]	[-0.06,-0.02]	[-0.06,-0.01]	[-0.05,0.02]
BA+		0.012	0.020		0.014	0.012		0.015	0.015		0.035	0.016
SE		(0.00)	(0.01)		(0.00)	(0.01)		(0.01)	(0.01)		(0.01)	(0.01)
Pvalue		0.003	0.001		0.001	0.040		0.108	0.231		0.000	0.227
95% CI		[0.00,0.02]	[0.01,0.03]		[0.01,0.02]	[0.00,0.02]		[-0.00,0.03]	[-0.01,0.04]		[0.02,0.05]	[-0.01,0.04]
Black		-0.011	-0.010		-0.014	-0.022		-0.002	-0.008		0.011	-0.003
SE		(0.01)	(0.01)		(0.01)	(0.01)		(0.01)	(0.02)		(0.01)	(0.02)
Pvalue		0.075	0.265		0.033	0.028		0.907	0.684		0.407	0.888
95% CI		[-0.02,0.00]	[-0.03,0.01]		[-0.03,-0.00]	[-0.04,-0.00]		[-0.03,0.02]	[-0.05,0.03]		[-0.01,0.04]	[-0.05,0.04]
Hispanic		-0.014	-0.018		-0.019	-0.006		-0.012	-0.021		-0.020	-0.018
SE		(0.01)	(0.01)		(0.01)	(0.01)		(0.02)	(0.02)		(0.02)	(0.02)
Pvalue		0.064	0.172		0.017	0.656		0.460	0.383		0.182	0.442
95% CI		[-0.03,0.00]	[-0.04,0.01]		[-0.03,-0.00]	[-0.03,0.02]		[-0.04,0.02]	[-0.07,0.03]		[-0.05,0.01]	[-0.06,0.03]
Other Race		-0.022	-0.035		-0.039	-0.056		-0.014	-0.044		-0.036	-0.065
SE		(0.01)	(0.01)		(0.01)	(0.01)		(0.02)	(0.03)		(0.02)	(0.03)
Pvalue		0.003	0.009		0.000	0.000		0.442	0.119		0.072	0.039
95% CI		[-0.04,-0.01]	[-0.06,-0.01]		[-0.05,-0.02]	[-0.08,-0.03]		[-0.05,0.02]	[-0.10,0.01]		[-0.07,0.00]	[-0.13,-0.00]
Age		0.002	0.002		0.002	0.002		0.002	0.002		0.002	0.002
SE		(0.00)	(0.00)		(0.00)	(0.00)		(0.00)	(0.00)		(0.00)	(0.00)
Pvalue		0.000	0.000		0.000	0.000		0.000	0.000		0.000	0.000
95% CI		[0.00,0.00]	[0.00,0.00]		[0.00,0.00]	[0.00,0.00]		[0.00,0.00]	[0.00,0.00]		[0.00,0.00]	[0.00,0.00]
Male		0.010	0.012		-0.004	-0.001		0.026	0.029		-0.011	0.002
SE		(0.00)	(0.01)		(0.00)	(0.01)		(0.01)	(0.01)		(0.01)	(0.01)
Pvalue		0.015	0.045		0.339	0.918		0.003	0.023		0.205	0.852
95% CI		[0.00,0.02]	[0.00,0.02]		[-0.01,0.00]	[-0.01,0.01]		[0.01,0.04]	[0.00,0.05]		[-0.03,0.01]	[-0.02,0.03]
Hlth Ins		0.064	0.069		0.071	0.066		0.025	0.069		0.056	0.059
SE		(0.01)	(0.02)		(0.01)	(0.02)		(0.01)	(0.02)		(0.02)	(0.02)
Pvalue		0.000	0.000		0.000	0.000		0.083	0.005		0.000	0.011
95% CI		[0.05,0.08]	[0.04,0.10]		[0.05,0.09]	[0.04,0.10]		[-0.00,0.05]	[0.02,0.12]		[0.03,0.09]	[0.01,0.10]
Inc $\geq$ 100K		0.031	0.029		0.035	0.047		0.031	0.016		0.071	0.080
SE		(0.01)	(0.01)		(0.01)	(0.01)		(0.02)	(0.02)		(0.02)	(0.02)
Pvalue		0.000	0.001		0.000	0.000		0.044	0.482		0.000	0.000
95% CI		[0.02,0.04]	[0.01,0.05]		[0.02,0.05]	[0.03,0.07]		[0.00,0.06]	[-0.03,0.06]		[0.04,0.10]	[0.04,0.12]
25K$<$ Inc $<$100K		0.016	0.012		0.017	0.017		0.026	0.026		0.040	0.047
SE		(0.01)	(0.01)		(0.01)	(0.01)		(0.01)	(0.02)		(0.01)	(0.02)
Pvalue		0.002	0.118		0.003	0.036		0.017	0.098		0.000	0.003
95% CI		[0.01,0.03]	[-0.00,0.03]		[0.01,0.03]	[0.00,0.03]		[0.00,0.05]	[-0.00,0.06]		[0.02,0.06]	[0.02,0.08]
Rural		-0.007	-0.013		-0.004	-0.003		-0.034	-0.028		-0.011	-0.002
SE		(0.01)	(0.01)		(0.01)	(0.01)		(0.01)	(0.02)		(0.01)	(0.02)
Pvalue		0.215	0.098		0.545	0.762		0.005	0.107		0.323	0.912
95% CI		[-0.02,0.00]	[-0.03,0.00]		[-0.02,0.01]	[-0.02,0.01]		[-0.06,-0.01]	[-0.06,0.01]		[-0.03,0.01]	[-0.04,0.03]
Constant	0.855	0.701	0.691	0.766	0.609	0.605	0.759	0.597	0.561	0.734	0.541	0.533
SE	(0.00)	(0.01)	(0.02)	(0.00)	(0.01)	(0.02)	(0.01)	(0.02)	(0.04)	(0.01)	(0.02)	(0.03)
Pvalue	0.000	0.000	0.000	0.000	0.000	0.000	0.000	0.000	0.000	0.000	0.000	0.000
95% CI	[0.85,0.86]	[0.68,0.72]	[0.65,0.73]	[0.76,0.77]	[0.59,0.63]	[0.56,0.64]	[0.74,0.77]	[0.55,0.64]	[0.49,0.63]	[0.72,0.75]	[0.50,0.58]	[0.47,0.60]
N	17242	17223	7696	17290	17271	7724	4773	4768	2197	4774	4769	2199

Note. Variable "Party ID" is 7 point partisanship scale coded from 0-1. Please note that each variable is reported with the regression coefficient, standard error (in parentheses), p-value and 95% confidence interval (in brackets).

Supplementary Table 19: Multi-Variable Analysis: Partisanship

	(1) Chest Visit (All)	(2) Chest Visit (All)	(3) Chest Visit (DX)	(4) PCP Visit (All)	(5) PCP Visit (All)	(6) PCP Visit (DX)	(7) ER Visit (All)	(8) ER Visit (All)	(9) ER Visit (DX)	(10) BMQ (DX)	(11) BMQ (DX)
Party ID	-0.047	-0.032	-0.024	-0.031	-0.023	-0.004	0.014	0.008	0.028	-0.024	-0.031
SE	(0.01)	(0.01)	(0.01)	(0.01)	(0.01)	(0.01)	(0.01)	(0.01)	(0.02)	(0.01)	(0.01)
Pvalue	0.000	0.000	0.017	0.001	0.010	0.703	0.122	0.410	0.082	0.001	0.000
95% CI	[-0.06,-0.03]	[-0.05,-0.02]	[-0.04,-0.00]	[-0.05,-0.01]	[-0.04,-0.01]	[-0.03,0.02]	[-0.00,0.03]	[-0.01,0.03]	[-0.00,0.06]	[-0.04,-0.01]	[-0.04,-0.02]
BA+		0.030	0.027		0.028	0.006		-0.051	-0.041		-0.005
SE		(0.00)	(0.01)		(0.01)	(0.01)		(0.01)	(0.01)		(0.01)
Pvalue		0.000	0.000		0.000	0.526		0.000	0.000		0.372
95% CI		[0.02,0.04]	[0.01,0.04]		[0.02,0.04]	[-0.01,0.02]		[-0.06,-0.04]	[-0.06,-0.02]		[-0.01,0.01]
Black		0.061	0.047		0.011	0.007		0.007	-0.016		-0.029
SE		(0.01)	(0.01)		(0.01)	(0.01)		(0.01)	(0.02)		(0.01)
Pvalue		0.000	0.000		0.252	0.623		0.499	0.338		0.001
95% CI		[0.05,0.07]	[0.02,0.07]		[-0.01,0.03]	[-0.02,0.03]		[-0.01,0.03]	[-0.05,0.02]		[-0.05,-0.01]
Hispanic		0.021	0.014		-0.033	-0.045		-0.002	0.057		-0.025
SE		(0.01)	(0.02)		(0.01)	(0.02)		(0.01)	(0.02)		(0.01)
Pvalue		0.017	0.339		0.008	0.023		0.842	0.017		0.024
95% CI		[0.00,0.04]	[-0.02,0.04]		[-0.06,-0.01]	[-0.08,-0.01]		[-0.03,0.02]	[0.01,0.10]		[-0.05,-0.00]
Other Race		0.007	0.002		-0.008	-0.027		-0.083	-0.074		-0.047
SE		(0.01)	(0.01)		(0.01)	(0.02)		(0.01)	(0.02)		(0.01)
Pvalue		0.434	0.893		0.493	0.170		0.000	0.001		0.000
95% CI		[-0.01,0.02]	[-0.03,0.03]		[-0.03,0.02]	[-0.06,0.01]		[-0.10,-0.06]	[-0.12,-0.03]		[-0.07,-0.02]
Age		0.003	0.002		0.004	0.004		-0.001	-0.002		0.002
SE		(0.00)	(0.00)		(0.00)	(0.00)		(0.00)	(0.00)		(0.00)
Pvalue		0.000	0.000		0.000	0.000		0.000	0.000		0.000
95% CI		[0.00,0.00]	[0.00,0.00]		[0.00,0.00]	[0.00,0.00]		[-0.00,-0.00]	[-0.00,-0.00]		[0.00,0.00]
Male		0.025	0.033		-0.026	-0.014		-0.027	-0.027		-0.013
SE		(0.00)	(0.01)		(0.01)	(0.01)		(0.01)	(0.01)		(0.01)
Pvalue		0.000	0.000		0.000	0.112		0.000	0.018		0.018
95% CI		[0.02,0.03]	[0.02,0.05]		[-0.04,-0.01]	[-0.03,0.00]		[-0.04,-0.01]	[-0.05,-0.00]		[-0.02,-0.00]
Hlth Ins		0.087	0.084		0.251	0.207		0.017	0.017		0.047
SE		(0.01)	(0.02)		(0.01)	(0.02)		(0.01)	(0.02)		(0.01)
Pvalue		0.000	0.000		0.000	0.000		0.134	0.459		0.000
95% CI		[0.07,0.10]	[0.05,0.11]		[0.23,0.28]	[0.16,0.25]		[-0.01,0.04]	[-0.03,0.06]		[0.03,0.07]
Inc $\geq$ 100K		0.026	0.017		0.045	0.053		-0.060	-0.055		-0.016
SE		(0.01)	(0.01)		(0.01)	(0.02)		(0.01)	(0.02)		(0.01)
Pvalue		0.002	0.166		0.000	0.000		0.000	0.004		0.059
95% CI		[0.01,0.04]	[-0.01,0.04]		[0.02,0.07]	[0.02,0.08]		[-0.08,-0.04]	[-0.09,-0.02]		[-0.03,0.00]
25K$<$ Inc $<$100K		0.009	-0.002		0.031	0.033		-0.029	-0.023		0.000
SE		(0.01)	(0.01)		(0.01)	(0.01)		(0.01)	(0.01)		(0.01)
Pvalue		0.164	0.818		0.000	0.003		0.001	0.110		0.957
95% CI		[-0.00,0.02]	[-0.02,0.02]		[0.01,0.05]	[0.01,0.06]		[-0.05,-0.01]	[-0.05,0.01]		[-0.01,0.01]
Rural		0.002	0.022		0.011	0.010		-0.009	-0.022		-0.012
SE		(0.01)	(0.01)		(0.01)	(0.01)		(0.01)	(0.02)		(0.01)
Pvalue		0.740	0.029		0.231	0.343		0.319	0.156		0.107
95% CI		[-0.01,0.02]	[0.00,0.04]		[-0.01,0.03]	[-0.01,0.03]		[-0.03,0.01]	[-0.05,0.01]		[-0.03,0.00]
Constant	0.678	0.411	0.462	0.794	0.359	0.455	0.217	0.334	0.401	0.589	0.477
SE	(0.00)	(0.01)	(0.02)	(0.01)	(0.02)	(0.03)	(0.01)	(0.02)	(0.03)	(0.00)	(0.01)
Pvalue	0.000	0.000	0.000	0.000	0.000	0.000	0.000	0.000	0.000	0.000	0.000
95% CI	[0.67,0.69]	[0.39,0.43]	[0.42,0.50]	[0.78,0.80]	[0.32,0.39]	[0.40,0.51]	[0.21,0.23]	[0.30,0.37]	[0.34,0.46]	[0.58,0.60]	[0.45,0.51]
N	21352	21327	8668	21694	21666	8746	21694	21666	8746	5950	5945

Note. Variable “Party ID” is 7 point partisanship scale coded from 0-1. Please note that each variable is reported with the regression coefficient, standard error (in parentheses), p-value and 95% confidence interval (in brackets).

Supplementary Table 20: Multi-Variable Analysis: Ideology

	Primary Care Provider						Emergency Room Doctor					
	(1)	(2)	(3)	(4)	(5)	(6)	(7)	(8)	(9)	(10)	(11)	(12)
	Trust (All)	Trust (All)	Trust (DX)	Advice (All)	Advice (All)	Advice (DX)	Trust (All)	Trust (All)	Trust (DX)	Advice (All)	Advice (All)	Advice (DX)
Ideology	-0.020	-0.035	-0.037	-0.010	-0.025	-0.021	-0.001	-0.014	-0.031	-0.005	-0.011	-0.009
SE	(0.01)	(0.01)	(0.01)	(0.01)	(0.01)	(0.01)	(0.02)	(0.02)	(0.03)	(0.02)	(0.02)	(0.03)
Pvalue	0.007	0.000	0.001	0.191	0.001	0.060	0.968	0.420	0.233	0.772	0.538	0.710
95% CI	[-0.03,-0.01]	[-0.05,-0.02]	[-0.06,-0.02]	[-0.02,0.00]	[-0.04,-0.01]	[-0.04,0.00]	[-0.04,0.03]	[-0.05,0.02]	[-0.08,0.02]	[-0.04,0.03]	[-0.04,0.02]	[-0.06,0.04]
BA+		0.013	0.020		0.016	0.013		0.016	0.016		0.037	0.018
SE		(0.00)	(0.01)		(0.00)	(0.01)		(0.01)	(0.01)		(0.01)	(0.01)
Pvalue		0.001	0.001		0.000	0.029		0.079	0.201		0.000	0.187
95% CI		[0.00,0.02]	[0.01,0.03]		[0.01,0.02]	[0.00,0.02]		[-0.00,0.03]	[-0.01,0.04]		[0.02,0.05]	[-0.01,0.04]
Black		-0.005	-0.005		-0.008	-0.017		0.005	-0.004		0.017	-0.000
SE		(0.01)	(0.01)		(0.01)	(0.01)		(0.01)	(0.02)		(0.01)	(0.02)
Pvalue		0.411	0.520		0.224	0.076		0.665	0.823		0.195	0.990
95% CI		[-0.02,0.01]	[-0.02,0.01]		[-0.02,0.00]	[-0.04,0.00]		[-0.02,0.03]	[-0.04,0.03]		[-0.01,0.04]	[-0.04,0.04]
Hispanic		-0.012	-0.017		-0.017	-0.004		-0.010	-0.023		-0.022	-0.021
SE		(0.01)	(0.01)		(0.01)	(0.01)		(0.02)	(0.02)		(0.02)	(0.02)
Pvalue		0.103	0.201		0.036	0.736		0.519	0.322		0.157	0.381
95% CI		[-0.03,0.00]	[-0.04,0.01]		[-0.03,-0.00]	[-0.03,0.02]		[-0.04,0.02]	[-0.07,0.02]		[-0.05,0.01]	[-0.07,0.03]
Other Race		-0.021	-0.034		-0.037	-0.054		-0.014	-0.045		-0.036	-0.066
SE		(0.01)	(0.01)		(0.01)	(0.01)		(0.02)	(0.03)		(0.02)	(0.03)
Pvalue		0.005	0.010		0.000	0.000		0.441	0.112		0.073	0.036
95% CI		[-0.03,-0.01]	[-0.06,-0.01]		[-0.05,-0.02]	[-0.08,-0.03]		[-0.05,0.02]	[-0.10,0.01]		[-0.08,0.00]	[-0.13,-0.00]
Age		0.002	0.002		0.002	0.002		0.002	0.003		0.002	0.002
SE		(0.00)	(0.00)		(0.00)	(0.00)		(0.00)	(0.00)		(0.00)	(0.00)
Pvalue		0.000	0.000		0.000	0.000		0.000	0.000		0.000	0.000
95% CI		[0.00,0.00]	[0.00,0.00]		[0.00,0.00]	[0.00,0.00]		[0.00,0.00]	[0.00,0.00]		[0.00,0.00]	[0.00,0.00]
Male		0.010	0.012		-0.004	-0.001		0.026	0.028		-0.013	0.002
SE		(0.00)	(0.01)		(0.00)	(0.01)		(0.01)	(0.01)		(0.01)	(0.01)
Pvalue		0.017	0.043		0.310	0.912		0.004	0.026		0.133	0.896
95% CI		[0.00,0.02]	[0.00,0.02]		[-0.01,0.00]	[-0.01,0.01]		[0.01,0.04]	[0.00,0.05]		[-0.03,0.00]	[-0.02,0.03]
Hlth Ins		0.064	0.068		0.070	0.065		0.024	0.069		0.058	0.057
SE		(0.01)	(0.02)		(0.01)	(0.02)		(0.01)	(0.02)		(0.02)	(0.02)
Pvalue		0.000	0.000		0.000	0.000		0.098	0.006		0.000	0.014
95% CI		[0.05,0.08]	[0.03,0.10]		[0.05,0.09]	[0.03,0.09]		[-0.00,0.05]	[0.02,0.12]		[0.03,0.09]	[0.01,0.10]
Inc $\geq$ 100K		0.030	0.029		0.034	0.046		0.030	0.013		0.072	0.079
SE		(0.01)	(0.01)		(0.01)	(0.01)		(0.02)	(0.02)		(0.02)	(0.02)
Pvalue		0.000	0.001		0.000	0.000		0.050	0.563		0.000	0.000
95% CI		[0.02,0.04]	[0.01,0.05]		[0.02,0.05]	[0.03,0.07]		[-0.00,0.06]	[-0.03,0.06]		[0.04,0.10]	[0.03,0.12]
25K< Inc <100K		0.015	0.012		0.015	0.016		0.023	0.023		0.038	0.045
SE		(0.01)	(0.01)		(0.01)	(0.01)		(0.01)	(0.02)		(0.01)	(0.02)
Pvalue		0.004	0.115		0.006	0.037		0.030	0.158		0.000	0.005
95% CI		[0.00,0.03]	[-0.00,0.03]		[0.00,0.03]	[0.00,0.03]		[0.00,0.04]	[-0.01,0.05]		[0.02,0.06]	[0.01,0.08]
Rural		-0.008	-0.013		-0.005	-0.002		-0.036	-0.033		-0.015	-0.008
SE		(0.01)	(0.01)		(0.01)	(0.01)		(0.01)	(0.02)		(0.01)	(0.02)
Pvalue		0.172	0.106		0.454	0.775		0.004	0.058		0.192	0.652
95% CI		[-0.02,0.00]	[-0.03,0.00]		[-0.02,0.01]	[-0.02,0.01]		[-0.06,-0.01]	[-0.07,0.00]		[-0.04,0.01]	[-0.04,0.03]
Constant	0.853	0.701	0.695	0.759	0.606	0.604	0.745	0.591	0.565	0.717	0.529	0.530
SE	(0.00)	(0.01)	(0.02)	(0.00)	(0.01)	(0.02)	(0.01)	(0.02)	(0.04)	(0.01)	(0.02)	(0.03)
Pvalue	0.000	0.000	0.000	0.000	0.000	0.000	0.000	0.000	0.000	0.000	0.000	0.000
95% CI	[0.84,0.86]	[0.68,0.72]	[0.65,0.74]	[0.75,0.77]	[0.58,0.63]	[0.56,0.64]	[0.73,0.77]	[0.55,0.63]	[0.50,0.63]	[0.70,0.74]	[0.49,0.57]	[0.46,0.60]
N	17268	17249	7707	17316	17297	7735	4785	4780	2204	4786	4781	2206

Note. Variable “Ideology” is 7 point liberal-conservative self-identification scale coded from 0-1. Please note that each variable is reported with the regression coefficient, standard error (in parentheses), p-value and 95% confidence interval (in brackets).

Supplementary Table 21: Multi-Variable Analysis: Ideology

	(1) Chest Visit (All)	(2) Chest Visit (All)	(3) Chest Visit (DX)	(4) PCP Visit (All)	(5) PCP Visit (All)	(6) PCP Visit (DX)	(7) ER Visit (All)	(8) ER Visit (All)	(9) ER Visit (DX)	(10) BMQ (DX)	(11) BMQ (DX)
Ideology	0.003	-0.010	-0.009	0.024	-0.002	-0.005	-0.016	-0.017	0.002	-0.029	-0.044
SE	(0.01)	(0.01)	(0.01)	(0.01)	(0.01)	(0.02)	(0.01)	(0.01)	(0.02)	(0.01)	(0.01)
Pvalue	0.736	0.267	0.520	0.060	0.882	0.767	0.214	0.173	0.934	0.003	0.000
95% CI	[-0.01,0.02]	[-0.03,0.01]	[-0.04,0.02]	[-0.00,0.05]	[-0.03,0.02]	[-0.04,0.03]	[-0.04,0.01]	[-0.04,0.01]	[-0.04,0.04]	[-0.05,-0.01]	[-0.06,-0.03]
BA+		0.031	0.029		0.030	0.006		-0.053	-0.044		-0.004
SE		(0.00)	(0.01)		(0.01)	(0.01)		(0.01)	(0.01)		(0.01)
Pvalue		0.000	0.000		0.000	0.470		0.000	0.000		0.437
95% CI		[0.02,0.04]	[0.01,0.04]		[0.02,0.04]	[-0.01,0.02]		[-0.07,-0.04]	[-0.07,-0.02]		[-0.01,0.01]
Black		0.069	0.053		0.018	0.009		0.003	-0.025		-0.023
SE		(0.01)	(0.01)		(0.01)	(0.01)		(0.01)	(0.02)		(0.01)
Pvalue		0.000	0.000		0.061	0.506		0.745	0.136		0.007
95% CI		[0.06,0.08]	[0.03,0.07]		[-0.00,0.04]	[-0.02,0.04]		[-0.02,0.02]	[-0.06,0.01]		[-0.04,-0.01]
Hispanic		0.025	0.017		-0.032	-0.046		-0.004	0.054		-0.024
SE		(0.01)	(0.02)		(0.01)	(0.02)		(0.01)	(0.02)		(0.01)
Pvalue		0.005	0.267		0.011	0.020		0.744	0.022		0.030
95% CI		[0.01,0.04]	[-0.01,0.05]		[-0.06,-0.01]	[-0.08,-0.01]		[-0.03,0.02]	[0.01,0.10]		[-0.05,-0.00]
Other Race		0.010	0.003		-0.007	-0.026		-0.084	-0.076		-0.046
SE		(0.01)	(0.01)		(0.01)	(0.02)		(0.01)	(0.02)		(0.01)
Pvalue		0.267	0.841		0.572	0.178		0.000	0.001		0.000
95% CI		[-0.01,0.03]	[-0.03,0.03]		[-0.03,0.02]	[-0.06,0.01]		[-0.11,-0.06]	[-0.12,-0.03]		[-0.07,-0.02]
Age		0.003	0.002		0.004	0.004		-0.001	-0.002		0.002
SE		(0.00)	(0.00)		(0.00)	(0.00)		(0.00)	(0.00)		(0.00)
Pvalue		0.000	0.000		0.000	0.000		0.000	0.000		0.000
95% CI		[0.00,0.00]	[0.00,0.00]		[0.00,0.00]	[0.00,0.00]		[-0.00,-0.00]	[-0.00,-0.00]		[0.00,0.00]
Male		0.024	0.032		-0.027	-0.015		-0.025	-0.025		-0.013
SE		(0.00)	(0.01)		(0.01)	(0.01)		(0.01)	(0.01)		(0.01)
Pvalue		0.000	0.000		0.000	0.085		0.000	0.028		0.015
95% CI		[0.01,0.03]	[0.02,0.05]		[-0.04,-0.01]	[-0.03,0.00]		[-0.04,-0.01]	[-0.05,-0.00]		[-0.02,-0.00]
Hlth Ins		0.088	0.085		0.251	0.208		0.014	0.014		0.049
SE		(0.01)	(0.02)		(0.01)	(0.02)		(0.01)	(0.02)		(0.01)
Pvalue		0.000	0.000		0.000	0.000		0.217	0.522		0.000
95% CI		[0.07,0.10]	[0.06,0.11]		[0.23,0.28]	[0.16,0.25]		[-0.01,0.04]	[-0.03,0.06]		[0.03,0.07]
Inc $\geq$ 100K		0.025	0.018		0.046	0.055		-0.059	-0.053		-0.017
SE		(0.01)	(0.01)		(0.01)	(0.02)		(0.01)	(0.02)		(0.01)
Pvalue		0.002	0.156		0.000	0.000		0.000	0.005		0.052
95% CI		[0.01,0.04]	[-0.01,0.04]		[0.02,0.07]	[0.02,0.08]		[-0.08,-0.04]	[-0.09,-0.02]		[-0.03,0.00]
25K$<$ Inc $<$100K		0.008	-0.003		0.030	0.035		-0.028	-0.021		0.001
SE		(0.01)	(0.01)		(0.01)	(0.01)		(0.01)	(0.01)		(0.01)
Pvalue		0.225	0.771		0.000	0.002		0.002	0.149		0.934
95% CI		[-0.00,0.02]	[-0.02,0.02]		[0.01,0.05]	[0.01,0.06]		[-0.05,-0.01]	[-0.05,0.01]		[-0.01,0.01]
Rural		0.000	0.019		0.009	0.009		-0.009	-0.021		-0.012
SE		(0.01)	(0.01)		(0.01)	(0.01)		(0.01)	(0.02)		(0.01)
Pvalue		0.990	0.057		0.312	0.409		0.326	0.166		0.118
95% CI		[-0.01,0.01]	[-0.00,0.04]		[-0.01,0.03]	[-0.01,0.03]		[-0.03,0.01]	[-0.05,0.01]		[-0.03,0.00]
Constant	0.654	0.398	0.452	0.767	0.349	0.452	0.232	0.347	0.414	0.593	0.480
SE	(0.01)	(0.01)	(0.02)	(0.01)	(0.02)	(0.03)	(0.01)	(0.02)	(0.03)	(0.01)	(0.01)
Pvalue	0.000	0.000	0.000	0.000	0.000	0.000	0.000	0.000	0.000	0.000	0.000
95% CI	[0.64,0.66]	[0.37,0.42]	[0.41,0.49]	[0.75,0.78]	[0.31,0.38]	[0.39,0.51]	[0.22,0.25]	[0.31,0.38]	[0.35,0.48]	[0.58,0.60]	[0.45,0.51]
N	21385	21360	8684	21722	21694	8760	21722	21694	8760	5958	5953

Note. Variable "Ideology" is 7 point liberal-conservative self-identification scale coded from 0-1. Please note that each variable is reported with the regression coefficient, standard error (in parentheses), p-value and 95% confidence interval (in brackets).

9 Correlates of Ideology

This section presents several tables unpacking the relationship between ideology, vote choice, and partisanship in the CHIP50 survey. Supplementary Tables 22 and 23 show the frequencies of ideological and partisan categories among respondents. Supplementary Tables 24 and 25 respectively show vote choice and partisanship among members of each ideological group. Supplementary Tables 26 and 27 show the results of regressing healthcare engagement outcomes on vote choice—only among moderates.

Supplementary Table 22: Ideology Frequency

Extremely Liberal	6.89
Liberal	12.48
Slightly Liberal	9.05
Moderate	38.96
Slightly Conservative	9.94
Conservative	15.82
Extremely Conservative	6.85
Total	100.00

Note. Percent of respondents by ideological subgroup.

Supplementary Table 23: Partisanship Frequency

Strong Democrat	20.15
Democrat	14.30
Lean Democrat	8.87
Independent	19.90
Lean Republican	7.56
Republican	12.19
Strong Republican	17.02
Total	100.00

Note. Percent of respondents by partisan subgroup.

Supplementary Table 24: Cross-tabs of Ideology & Vote Choice

	Extremely Liberal	Liberal	Slightly Liberal	Moderate	Slightly Conservative	Conservative	Extremely Conservative	Total
Biden	75.58	73.17	61.01	32.96	17.11	11.73	8.06	36.82
Trump	8.49	8.49	15.95	30.53	57.55	72.85	80.88	37.77
Other/DK/Not voting	15.93	18.34	23.04	36.51	25.34	15.42	11.06	25.41
Total	100.00	100.00	100.00	100.00	100.00	100.00	100.00	100.00

Note. Percent of respondents by ideology & vote choice.

Supplementary Table 25: Cross-tabs of Partisanship & Ideology

	Extremely Liberal	Liberal	Slightly Liberal	Moderate	Slightly Conservative	Conservative	Extremely Conservative	Total
Strong Democrat	65.65	54.30	21.09	12.43	6.15	6.94	6.08	20.16
Democrat	5.36	18.42	36.45	16.55	9.90	5.07	1.60	14.31
Lean Democrat	11.92	10.70	20.17	10.35	4.30	2.25	1.11	8.88
Independent	12.25	8.90	11.09	34.16	13.21	9.72	10.62	19.85
Lean Republican	0.58	1.37	2.00	8.36	18.98	9.96	6.46	7.56
Republican	0.35	1.86	5.27	11.40	35.64	20.34	3.86	12.20
Strong Republican	3.89	4.45	3.92	6.75	11.82	45.72	70.28	17.03
Total	100.00	100.00	100.00	100.00	100.00	100.00	100.00	100.00

Note. Percent of respondents by ideology and partisanship.

Supplementary Table 26: Vote Choice & Engagement Among Moderates

	Primary Care Provider						Emergency Room Doctor					
	(1) Trust (All)	(2) Trust (All)	(3) Trust (DX)	(4) Advice (All)	(5) Advice (All)	(6) Advice (DX)	(7) Trust (All)	(8) Trust (All)	(9) Trust (DX)	(10) Advice (All)	(11) Advice (All)	(12) Advice (DX)
Vote Trump	-0.041	-0.027	-0.015	-0.036	-0.025	-0.011	-0.081	-0.049	-0.034	-0.059	-0.014	0.008
SE	(0.01)	(0.01)	(0.01)	(0.01)	(0.01)	(0.01)	(0.02)	(0.02)	(0.03)	(0.02)	(0.02)	(0.03)
Pvalue	0.000	0.001	0.187	0.000	0.003	0.356	0.000	0.006	0.184	0.001	0.471	0.752
95% CI	[-0.06,-0.03]	[-0.04,-0.01]	[-0.04,0.01]	[-0.05,-0.02]	[-0.04,-0.01]	[-0.04,0.01]	[-0.11,-0.05]	[-0.08,-0.01]	[-0.09,0.02]	[-0.09,-0.03]	[-0.05,0.02]	[-0.04,0.06]
BA+		0.001	0.006		0.006	0.017		0.037	0.020		0.043	0.031
SE		(0.01)	(0.01)		(0.01)	(0.01)		(0.02)	(0.03)		(0.02)	(0.03)
Pvalue		0.856	0.550		0.440	0.144		0.039	0.441		0.021	0.253
95% CI		[-0.01,0.02]	[-0.01,0.03]		[-0.01,0.02]	[-0.01,0.04]		[0.00,0.07]	[-0.03,0.07]		[0.01,0.08]	[-0.02,0.08]
Black		-0.016	-0.022		-0.021	-0.030		-0.001	-0.007		0.034	-0.010
SE		(0.01)	(0.02)		(0.01)	(0.02)		(0.02)	(0.03)		(0.03)	(0.04)
Pvalue		0.136	0.181		0.073	0.102		0.955	0.838		0.206	0.819
95% CI		[-0.04,0.00]	[-0.05,0.01]		[-0.04,0.00]	[-0.07,0.01]		[-0.05,0.04]	[-0.07,0.06]		[-0.02,0.09]	[-0.09,0.07]
Hispanic		-0.004	0.004		-0.022	-0.017		-0.026	-0.022		-0.008	0.004
SE		(0.01)	(0.02)		(0.02)	(0.02)		(0.03)	(0.05)		(0.03)	(0.04)
Pvalue		0.806	0.873		0.157	0.480		0.420	0.653		0.792	0.925
95% CI		[-0.03,0.03]	[-0.04,0.05]		[-0.05,0.01]	[-0.06,0.03]		[-0.09,0.04]	[-0.12,0.07]		[-0.06,0.05]	[-0.07,0.08]
Other Race		-0.033	-0.037		-0.052	-0.073		-0.028	-0.005		-0.097	-0.208
SE		(0.01)	(0.02)		(0.01)	(0.03)		(0.04)	(0.05)		(0.05)	(0.07)
Pvalue		0.027	0.131		0.000	0.004		0.499	0.929		0.050	0.005
95% CI		[-0.06,-0.00]	[-0.09,0.01]		[-0.08,-0.02]	[-0.12,-0.02]		[-0.11,0.05]	[-0.10,0.10]		[-0.19,0.00]	[-0.35,-0.06]
Age		0.001	0.002		0.001	0.002		0.002	0.002		0.002	0.002
SE		(0.00)	(0.00)		(0.00)	(0.00)		(0.00)	(0.00)		(0.00)	(0.00)
Pvalue		0.000	0.000		0.000	0.000		0.003	0.044		0.000	0.023
95% CI		[0.00,0.00]	[0.00,0.00]		[0.00,0.00]	[0.00,0.00]		[0.00,0.00]	[0.00,0.00]		[0.00,0.00]	[0.00,0.00]
Male		0.020	0.014		0.001	0.011		0.016	0.024		0.008	0.035
SE		(0.01)	(0.01)		(0.01)	(0.01)		(0.02)	(0.02)		(0.02)	(0.02)
Pvalue		0.009	0.180		0.852	0.326		0.361	0.333		0.631	0.147
95% CI		[0.00,0.04]	[-0.01,0.04]		[-0.01,0.02]	[-0.01,0.03]		[-0.02,0.05]	[-0.02,0.07]		[-0.03,0.04]	[-0.01,0.08]
Hlth Ins		0.046	0.094		0.064	0.083		0.036	0.150		0.042	0.031
SE		(0.02)	(0.03)		(0.02)	(0.03)		(0.03)	(0.05)		(0.03)	(0.04)
Pvalue		0.010	0.007		0.000	0.006		0.210	0.003		0.160	0.420
95% CI		[0.01,0.08]	[0.03,0.16]		[0.03,0.10]	[0.02,0.14]		[-0.02,0.09]	[0.05,0.25]		[-0.02,0.10]	[-0.04,0.11]
Inc $\geq$ 100K		0.023	0.026		0.021	0.012		0.040	0.049		0.076	0.047
SE		(0.01)	(0.02)		(0.01)	(0.02)		(0.03)	(0.04)		(0.03)	(0.04)
Pvalue		0.059	0.129		0.113	0.532		0.154	0.209		0.010	0.249
95% CI		[-0.00,0.05]	[-0.01,0.06]		[-0.00,0.05]	[-0.03,0.05]		[-0.01,0.09]	[-0.03,0.13]		[0.02,0.13]	[-0.03,0.13]
25K$<$ Inc $<$100K		0.004	0.010		0.010	0.000		0.024	0.044		0.026	0.008
SE		(0.01)	(0.01)		(0.01)	(0.01)		(0.02)	(0.03)		(0.02)	(0.03)
Pvalue		0.670	0.470		0.338	0.992		0.249	0.113		0.214	0.767
95% CI		[-0.02,0.02]	[-0.02,0.04]		[-0.01,0.03]	[-0.03,0.03]		[-0.02,0.06]	[-0.01,0.10]		[-0.01,0.07]	[-0.05,0.06]
Rural		0.005	-0.004		0.001	-0.011		-0.022	0.017		-0.014	-0.018
SE		(0.01)	(0.02)		(0.01)	(0.02)		(0.02)	(0.03)		(0.02)	(0.04)
Pvalue		0.688	0.766		0.926	0.471		0.318	0.595		0.570	0.630
95% CI		[-0.02,0.03]	[-0.03,0.03]		[-0.02,0.02]	[-0.04,0.02]		[-0.07,0.02]	[-0.05,0.08]		[-0.06,0.03]	[-0.09,0.06]
Constant	0.867	0.734	0.678	0.779	0.647	0.614	0.795	0.646	0.524	0.757	0.560	0.586
SE	(0.00)	(0.02)	(0.05)	(0.01)	(0.02)	(0.04)	(0.01)	(0.04)	(0.07)	(0.01)	(0.05)	(0.07)
Pvalue	0.000	0.000	0.000	0.000	0.000	0.000	0.000	0.000	0.000	0.000	0.000	0.000
95% CI	[0.86,0.88]	[0.69,0.78]	[0.59,0.77]	[0.77,0.79]	[0.60,0.69]	[0.53,0.70]	[0.77,0.82]	[0.56,0.73]	[0.39,0.66]	[0.73,0.78]	[0.46,0.66]	[0.46,0.72]
N	4305	4300	1960	4315	4310	1967	1219	1217	576	1219	1217	577

Note. Results presented just for those with identify as moderates (i.e., neither liberal or conservative).

Supplementary Table 27: Vote Choice & Engagement Among Moderates

	(1)<br>Chest Visit (All)	(2)<br>Chest Visit (All)	(3)<br>Chest Visit (DX)	(4)<br>PCP Visit (All)	(5)<br>PCP Visit (All)	(6)<br>PCP Visit (DX)	(7)<br>ER Visit (All)	(8)<br>ER Visit (All)	(9)<br>ER Visit (DX)	(10)<br>BMQ (DX)	(11)<br>BMQ (DX)
Vote Trump	-0.070	-0.028	-0.024	-0.101	-0.045	-0.014	0.059	0.027	0.035	-0.034	-0.027
SE	(0.01)	(0.01)	(0.02)	(0.01)	(0.01)	(0.02)	(0.01)	(0.01)	(0.02)	(0.01)	(0.01)
Pvalue	0.000	0.005	0.113	0.000	0.001	0.454	0.000	0.066	0.155	0.001	0.019
95% CI	[-0.09,-0.05]	[-0.05,-0.01]	[-0.05,0.01]	[-0.13,-0.08]	[-0.07,-0.02]	[-0.05,0.02]	[0.03,0.09]	[-0.00,0.06]	[-0.01,0.08]	[-0.05,-0.01]	[-0.05,-0.00]
BA+		0.034	0.020		0.022	0.037		-0.066	-0.073		-0.013
SE		(0.01)	(0.02)		(0.01)	(0.02)		(0.01)	(0.02)		(0.01)
Pvalue		0.001	0.193		0.101	0.036		0.000	0.001		0.212
95% CI		[0.01,0.05]	[-0.01,0.05]		[-0.00,0.05]	[0.00,0.07]		[-0.09,-0.04]	[-0.12,-0.03]		[-0.03,0.01]
Black		0.050	0.025		0.023	0.001		0.006	0.012		-0.009
SE		(0.01)	(0.02)		(0.02)	(0.03)		(0.02)	(0.03)		(0.02)
Pvalue		0.000	0.209		0.219	0.965		0.744	0.720		0.557
95% CI		[0.02,0.07]	[-0.01,0.06]		[-0.01,0.06]	[-0.05,0.05]		[-0.03,0.04]	[-0.05,0.08]		[-0.04,0.02]
Hispanic		0.019	0.001		-0.025	-0.043		-0.020	0.013		-0.025
SE		(0.02)	(0.03)		(0.02)	(0.04)		(0.03)	(0.05)		(0.02)
Pvalue		0.270	0.967		0.317	0.276		0.415	0.783		0.294
95% CI		[-0.01,0.05]	[-0.05,0.06]		[-0.07,0.02]	[-0.12,0.03]		[-0.07,0.03]	[-0.08,0.10]		[-0.07,0.02]
Other Race		0.009	0.007		0.037	0.030		-0.122	-0.144		-0.040
SE		(0.02)	(0.03)		(0.02)	(0.03)		(0.02)	(0.04)		(0.02)
Pvalue		0.599	0.800		0.079	0.332		0.000	0.000		0.080
95% CI		[-0.03,0.04]	[-0.05,0.06]		[-0.00,0.08]	[-0.03,0.09]		[-0.16,-0.08]	[-0.22,-0.07]		[-0.08,0.00]
Age		0.002	0.002		0.004	0.004		-0.002	-0.003		0.001
SE		(0.00)	(0.00)		(0.00)	(0.00)		(0.00)	(0.00)		(0.00)
Pvalue		0.000	0.000		0.000	0.000		0.000	0.000		0.000
95% CI		[0.00,0.00]	[0.00,0.00]		[0.00,0.00]	[0.00,0.01]		[-0.00,-0.00]	[-0.00,-0.00]		[0.00,0.00]
Male		0.020	0.024		-0.003	0.002		-0.036	-0.035		0.002
SE		(0.01)	(0.01)		(0.01)	(0.02)		(0.01)	(0.02)		(0.01)
Pvalue		0.031	0.087		0.790	0.890		0.008	0.114		0.824
95% CI		[0.00,0.04]	[-0.00,0.05]		[-0.03,0.02]	[-0.03,0.03]		[-0.06,-0.01]	[-0.08,0.01]		[-0.02,0.02]
Hlth Ins		0.070	-0.004		0.232	0.178		-0.002	0.042		0.023
SE		(0.02)	(0.02)		(0.03)	(0.04)		(0.02)	(0.04)		(0.02)
Pvalue		0.000	0.867		0.000	0.000		0.928	0.328		0.176
95% CI		[0.04,0.10]	[-0.05,0.04]		[0.18,0.28]	[0.09,0.26]		[-0.05,0.04]	[-0.04,0.13]		[-0.01,0.06]
Inc $\geq$ 100K		0.004	-0.018		0.035	0.036		-0.013	-0.006		-0.011
SE		(0.02)	(0.02)		(0.02)	(0.03)		(0.02)	(0.04)		(0.02)
Pvalue		0.790	0.453		0.120	0.238		0.565	0.871		0.506
95% CI		[-0.03,0.03]	[-0.06,0.03]		[-0.01,0.08]	[-0.02,0.10]		[-0.06,0.03]	[-0.08,0.07]		[-0.04,0.02]
25K$<$ Inc $<$100K		0.004	-0.004		0.033	0.036		-0.017	-0.026		0.016
SE		(0.01)	(0.02)		(0.02)	(0.02)		(0.02)	(0.03)		(0.01)
Pvalue		0.745	0.789		0.040	0.091		0.309	0.346		0.240
95% CI		[-0.02,0.03]	[-0.04,0.03]		[0.00,0.06]	[-0.01,0.08]		[-0.05,0.02]	[-0.08,0.03]		[-0.01,0.04]
Rural		-0.015	0.002		-0.005	-0.012		-0.009	-0.009		-0.000
SE		(0.01)	(0.02)		(0.02)	(0.02)		(0.02)	(0.03)		(0.02)
Pvalue		0.264	0.921		0.782	0.621		0.635	0.759		0.995
95% CI		[-0.04,0.01]	[-0.04,0.04]		[-0.04,0.03]	[-0.06,0.03]		[-0.05,0.03]	[-0.07,0.05]		[-0.03,0.03]
Constant	0.703	0.483	0.594	0.843	0.387	0.454	0.205	0.374	0.451	0.593	0.490
SE	(0.01)	(0.03)	(0.04)	(0.01)	(0.04)	(0.06)	(0.01)	(0.04)	(0.07)	(0.01)	(0.03)
Pvalue	0.000	0.000	0.000	0.000	0.000	0.000	0.000	0.000	0.000	0.000	0.000
95% CI	[0.69,0.72]	[0.43,0.53]	[0.52,0.67]	[0.83,0.86]	[0.31,0.46]	[0.34,0.57]	[0.19,0.22]	[0.30,0.44]	[0.32,0.58]	[0.58,0.61]	[0.43,0.55]
N	5266	5259	2200	5345	5337	2215	5345	5337	2215	1489	1487

Note. Results presented just for those with identify as moderates (i.e., neither liberal or conservative).

10 Doctor Visits over Time

The main manuscript shows a gap in healthcare utilization in the post-COVID era, particularly by partisanship and vote choice. Has the left always been more likely to utilize care? And is this related to trust?

In an effort to address this question, we looked further for data on health care utilization over time by political affiliation. We identified three surveys spanning the 2010s that asked slight variations on whether the respondent had "Gone to a health care provider for a general physical exam."[1] These surveys echo a finding we present (in Figure 5) regarding whether the respondent had seen their primary care provider in the last 12 months.

Figure 7 shows that in in 2012 and 2016, Democrats and Republicans reported seeing their physician for an annual check-up at similar rates (2011: $\beta = -.001$ two-tailed t-test; $p = .978$; CI: -.07,.07; N=1,365. 2016: $\beta = .012$ two-tailed t-test; $p = .806$; CI: -.07,.11; N=1,410). In 2023, Republicans are 8.03 pp. less likely to report having had an annual physical screening ($\beta = -.0803$ two-tailed t-test; $p = .032$; CI: -.15,-.07; N=1,065).

Supplementary Figure 7: Had a physical/health screening in last 12 months

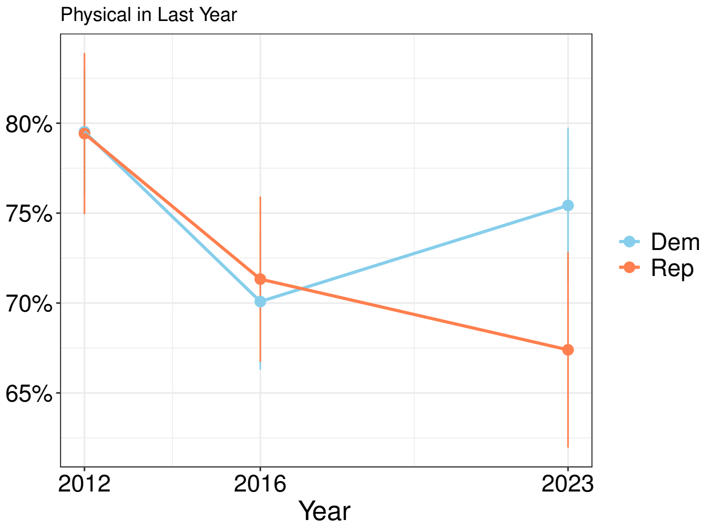

Note. Lines represent 95% confidence intervals around the average reporting having a physical in the prior year. Accompanying text presents full statistical reporting for the difference between the means.

The 2023 Axios survey also asks respondents, "How much trust do you have, if any, in information about health topics from the following? ... Your personal doctor." Figure 28 shows the cross-tab the relationship between people's trust in information from their personal doctor with whether they reported having seen a doctor for an annual physical screening. A clear gradient emerges: 80% of respondents who report having "a great deal of trust" report having had an an-

[1]Pew Research Center for the People & the Press (2016) asked this in 2016. Harvard School of Public Health/NPR/Robert Wood Johnson Foundation (2012) asked in 2012 "In the past 12 months, have you seen a doctor or nurse practitioner for a routine check-up, or not?" And Axios in 2023 asked, "In the last year, have you had an annual health screening with a physician" (Axios 2023).

nual physical exam in the prior year compared to just 27% of respondents who say they have "None at all."

Supplementary Table 28: Health screening in last 12 months by Trust Info From Personal Doctor

	None at all	Not very much	A fair amount	A great deal	Total
No	73.04	45.62	36.94	19.56	32.01
Yes	26.96	54.38	63.06	80.44	67.99
Total	100.00	100.00	100.00	100.00	100.00

11 Demographics

The tables below present the demographics of the CHIP50 survey 29 and waves 3, 4, and 5 of the Add Health survey (30, 31, and 32).

Supplementary Table 29: CHIP50 Survey (Weighted)

	Statistic	Obs	Mean	SD	Min	Max	Weight
1	BA	21751.00	0.36	0.48	0.00	1.00	1.00
2	White	21751.00	0.65	0.48	0.00	1.00	1.00
3	Black	21751.00	0.13	0.33	0.00	1.00	1.00
4	Hispanic	21751.00	0.16	0.37	0.00	1.00	1.00
5	Male	21751.00	0.49	0.50	0.00	1.00	1.00
6	Age	21751.00	47.62	17.72	18.00	96.00	1.00
7	Chronic Disease	21751.00	0.38	0.49	0.00	1.00	1.00
8	Trump ’24	16541.00	0.51	0.50	0.00	1.00	1.00

Supplementary Table 30: Wave 3, Add Health Survey (Weighted)

	Statistic	Wave	Mean	SD	Min	Max	Obs
1	Birth Year	3.00	1979.06	1.82	1974.00	1983.00	14314.00
2	NH White	3.00	0.66	0.47	0.00	1.00	14312.00
3	NH Black	3.00	0.15	0.36	0.00	1.00	14312.00
4	Hispanic	3.00	0.12	0.32	0.00	1.00	14282.00
5	BA+	3.00	0.14	0.35	0.00	1.00	14309.00
6	Over 100K	3.00	0.00	0.05	0.00	1.00	13433.00
7	Health Ins	3.00	0.75	0.43	0.00	1.00	14235.00
8	Male	3.00	0.51	0.50	0.00	1.00	14322.00
9	Ideology	3.00	3.04	0.75	1.00	5.00	12962.00

Supplementary Table 31: Wave 4, Add Health Survey (Weighted)

	Statistic	Wave	Mean	SD	Min	Max	Obs
1	Birth Year	4.00	1979.05	1.81	1974.00	1983.00	14790.00
2	NH White	4.00	0.66	0.47	0.00	1.00	14788.00
3	NH Black	4.00	0.15	0.36	0.00	1.00	14788.00
4	Hispanic	4.00	0.12	0.33	0.00	1.00	14756.00
5	BA+	4.00	0.30	0.46	0.00	1.00	14796.00
6	Over 100K	4.00	0.15	0.36	0.00	1.00	13824.00
7	Health Ins	4.00	0.80	1.51	0.00	96.00	14783.00
8	Male	4.00	0.51	0.50	0.00	1.00	14800.00
9	Ideology	4.00	2.97	0.92	1.00	5.00	13892.00

Supplementary Table 32: Wave 5, Add Health Survey (Weighted)

	Statistic	Wave	Mean	SD	Min	Max	Obs
1	Birth Year	5.00	1979.05	1.81	1974.00	1983.00	12293.00
2	NH White	5.00	0.66	0.47	0.00	1.00	12293.00
3	NH Black	5.00	0.15	0.36	0.00	1.00	12293.00
4	Hispanic	5.00	0.12	0.33	0.00	1.00	12261.00
5	BA+	5.00	0.37	0.48	0.00	1.00	12275.00
6	Over 100K	5.00	0.31	0.46	0.00	1.00	12122.00
7	Health Ins	5.00	0.90	0.30	0.00	1.00	12144.00
8	Male	5.00	0.50	0.50	0.00	1.00	12297.00
9	Ideology	5.00	3.07	0.95	1.00	5.00	11477.00

12 Supplemental Information References

Axios. 2023. Axios-Ipsos American Health Index—Wave 1, 2023 [Dataset]. Roper #31120137, Version 2. Ipsos [producer]. Cornell University, Ithaca, NY: Roper Center for Public Opinion Research [distributor]. doi:10.25940/ROPER-31120137

Davern, Michael; Bautista, Rene; Freese, Jeremy; Herd, Pamela; and Morgan, Stephen L.; General Social Survey 1972-2024. [Machine-readable data file]. Principal Investigator, Michael Davern; Co-Principal Investigators, Rene Bautista, Jeremy Freese, Pamela Herd, and Stephen L. Morgan. Sponsored by National Science Foundation. NORC ed. Chicago: NORC, 2025: NORC at the University of Chicago [producer and distributor].

Harvard School of Public Health/NPR/Robert Wood Johnson Foundation. 2012. NPR/Robert Wood Johnson Foundation/Harvard School of Public Health Poll: Sick in America, 2012 [Dataset]. Roper #31092354, Version 2. Social Science Research Solutions (SSRS) [producer]. Cornell University, Ithaca, NY: Roper Center for Public Opinion Research [distributor]. doi:10.25940/ROPER-31092354

Pew Research Center for the People & the Press. 2016. Pew Research Center: American Trends Panel Wave 17, 2016 [Dataset]. Roper #31114015, Version 2. Abt SRBI [producer]. Cornell University, Ithaca, NY: Roper Center for Public Opinion Research [distributor]. doi:10.25940/ROPER-31114015
