# Supplementary material for: The political polarization of health outcomes in the USA
Source: Nat Hum Behav. 2026 May 14;10(7):1234–45. doi: 10.1038/s41562-026-02474-9 (PMC13388103; doi:10.1038/s41562-026-02474-9)
Supplement: Supplementary file 2 — Reporting Summary [file 41562_2026_2474_MOESM2_ESM.pdf]

nature portfolio

Corresponding author(s): Neil O'Brian

Last updated by author(s): 02/13/2026

Reporting Summary

Nature Portfolio wishes to improve the reproducibility of the work that we publish. This form provides structure for consistency and transparency in reporting. For further information on Nature Portfolio policies, see our Editorial Policies and the Editorial Policy Checklist.

Statistics

For all statistical analyses, confirm that the following items are present in the figure legend, table legend, main text, or Methods section.

n/a	Confirmed	
☐	☒	The exact sample size (*n*) for each experimental group/condition, given as a discrete number and unit of measurement
☒	☐	A statement on whether measurements were taken from distinct samples or whether the same sample was measured repeatedly
☐	☒	The statistical test(s) used AND whether they are one- or two-sided<br>*Only common tests should be described solely by name; describe more complex techniques in the Methods section.*
☐	☒	A description of all covariates tested
☒	☐	A description of any assumptions or corrections, such as tests of normality and adjustment for multiple comparisons
☐	☒	A full description of the statistical parameters including central tendency (e.g. means) or other basic estimates (e.g. regression coefficient) AND variation (e.g. standard deviation) or associated estimates of uncertainty (e.g. confidence intervals)
☐	☒	For null hypothesis testing, the test statistic (e.g. *F*, *t*, *r*) with confidence intervals, effect sizes, degrees of freedom and *P* value noted<br>*Give P values as exact values whenever suitable.*
☒	☐	For Bayesian analysis, information on the choice of priors and Markov chain Monte Carlo settings
☒	☐	For hierarchical and complex designs, identification of the appropriate level for tests and full reporting of outcomes
☒	☐	Estimates of effect sizes (e.g. Cohen's *d*, Pearson's *r*), indicating how they were calculated

Our web collection on statistics for biologists contains articles on many of the points above.

Software and code

Policy information about availability of computer code

Data collection	We did not draw on any software to collect data, which was provided by third parties.
Data analysis	We used Stata 17 and R for data analysis. Code to produce all results and figures in the paper is available in our replication files at https://osf.io/q3gn5

For manuscripts utilizing custom algorithms or software that are central to the research but not yet described in published literature, software must be made available to editors and reviewers. We strongly encourage code deposition in a community repository (e.g. GitHub). See the Nature Portfolio guidelines for submitting code & software for further information.

Data

Policy information about availability of data

All manuscripts must include a data availability statement. This statement should provide the following information, where applicable:

- Accession codes, unique identifiers, or web links for publicly available datasets
- A description of any restrictions on data availability
- For clinical datasets or third party data, please ensure that the statement adheres to our policy

Add Health restricted-use data are available through contractual agreement, subject to data security and research justification requirements, through the process detailed at https://data.cpc.unc.edu/ projects/2/view; to protect participants' sensitive health data, these cannot be shared publicly. Original survey data is available at https://osf.io/q3gn5

Research involving human participants, their data, or biological material

Policy information about studies with human participants or human data. See also policy information about sex, gender (identity/presentation), and sexual orientation and race, ethnicity and racism.

Reporting on sex and gender	Three analyses in the paper rely on self-reported sex or gender. In the Add Health survey, respondents were asked "what is your sex?" in the initial survey (when they were children/teens in 1994-5, options male and female); in subsequent surveys, interviewers were instructed to confirm that respondents were the sex recorded in the prior survey, asking the respondent if necessary. In the CHIP50 survey, the survey vendor provided what they term "gender" information from respondent self-reports (options male and female). We refer to both as gender in the manuscript, as the survey items may be reasonably understood by respondents as asking about the gender with which they identify rather than their biological sex.<br><br>Because there has historically been little gap between American men and women in most political views, we did not expect findings to differ by gender and have not conducted analyses separately for male and female respondents. Gender entered into our analysis in three places: in Figure 3, as a potential explanation for changes in health; in Table 1, as a potential explanation for changes in mortality; and in Figure 5, as a control variable in explaining trust in medicine. In the former two analyses, gender is not predictive of changes in health/mortality to a substantively or statistically significant degree; in the latter, gender does not change the relationship between political beliefs and trust in medicine.
Reporting on race, ethnicity, or other socially relevant groupings	Three analyses in the paper rely on self-reported race or ethnicity. In the Add Health survey, respondents were asked "are you of Hispanic/Latino origin?" and, separately, "what is your race? (check all that apply)". In the CHIP50 survey, the survey vendor provided a "race" variable based on respondent self-reports. We use these questions to create indicator variables for membership in racial/ethnic groups.<br><br>These same analyses also categorize respondents by their gender (as described above), self-reported income, and self-reported education. Table 1 includes a control for respondent age (measured by their self-reported birth year). In all of these variables, we were limited to the information provided by the original data collectors. We use these measures only to account for their role as alternative or additional predictors of changes in health status, and we do not focus on or draw substantive conclusions about their independent roles in shaping health outcomes.<br><br>We do focus on respondents' self-reported political beliefs in all analyses. In Add Health, we analyze self-reported ideology based on a question asking respondents "In terms of politics, do you consider yourself conservative, liberal, or middle of-the-road?". In CHIP50, we rely on three measures of political beliefs: self-reported ideology (on a 7-point scale ranging from extremely liberal to extremely conservative), self-reported party affiliation (on a 7-point scale ranging from strong Republican to strong Democrat), and self-reported intended vote choice in the 2024 election (Trump, Biden, or someone else). These measures are widely used in political science research and highly predictive of political behavior, including policy attitudes and vote choice.<br><br>Figure 5 also separates out respondents who self-report having been diagnosed with one in a set of chronic diseases, which we selected for their commonness and for the role of regular medical treatment in their management. These responses were used only to identify a subpopulation for whom compliance with medical advice is especially important.
Population characteristics	The Add Health study is a nationally-representative sample of Americans in middle and high school in 1994-5. The CHIP50 study draws on a non-probability sample designed to approximate national characteristics through targeted recruiting, sample quotas, and poststratification weights. See below for further details.
Recruitment	The Add Health study participants were recruited through sampled high schools and middle schools; children not enrolled in school at the time of the study were outside the sampling frame. Only 2% of children were homeschooled in the 1990s, so their exclusion is unlikely to cause major changes in results. The focus of the study on this particular cohort limits our ability to draw conclusions about people of other ages, a matter we address in the paper.<br><br>The CHIP50 data were gathered from a broad set of survey vendors who "use a variety of strategies and incentives to maintain online respondent panels" and take steps in recruitment and sampling to match the demographic composition of the country and the 50 states. This strategy necessarily excludes the roughly 4% of Americans who do not have access to the Internet. However, CHIP50 data is able to recover national baselines on several important outcomes, including COVID-19 vaccination and infection rates and vote shares in the 2020 elections. We therefore do not expect significant biases in our results based on sample composition.
Ethics oversight	Human subjects review boards at the University of Oregon (STUDY00001107) and Stanford University ((protocol 14166)) approved our use of restricted-use data from Add Health. The PIs for Add Health obtained informed consent according to IRB regulations. We received only anonymized survey data from CHIP50, which does not constitute original human subjects research subject to board review. The CHIP50 survey project was approved by the PI's IRB at the University of Rochester (STUDY00009146) and informed consent was obtained.

Note that full information on the approval of the study protocol must also be provided in the manuscript.

Field-specific reporting

Please select the one below that is the best fit for your research. If you are not sure, read the appropriate sections before making your selection.

☐ Life sciences ☒ Behavioural & social sciences ☐ Ecological, evolutionary & environmental sciences

Behavioural & social sciences study design

All studies must disclose on these points even when the disclosure is negative.

Study description	This study draws on quantitative analysis of two surveys, one of which includes both self reports and medical test results, and one of which includes only self reports.
Research sample	The Add Health study is an existing nationally-representative sample of Americans in middle and high school in 1994-5, reinterviewed several times in the following years. The CHIP50 study draws on a non-probability sample designed to approximate national characteristics through targeted recruiting, sample quotas, and poststratification weights; we proposed questions to be added to a wave of this existing survey.
Sampling strategy	The Add Health study participants were recruited through sampled high schools and middle schools, designed to be nationally representative of this cohort. The initial sample contained 20,745 respondents, with attrition and replenishment in each subsequent wave. By waves 4 and 5, the core of our analysis, around 10,000 respondents remained with usable data. Because we did not participate in the study design or collection, we could not choose these sample sizes. The sample is 51% male with an average birth year of 1979. Further demographics can be found in supplemental information section 11.<br><br>The CHIP50 study draws on a non-probability sample designed to approximate national characteristics through targeted recruiting, sample quotas, and poststratification weights. We were able to include questions on a wave of the survey that gathered 21,751 responses; the exact number of responses was again outside our control, but we applied for administration to a large sample to allow for separate analysis of people with chronic illness. The sample is 49% male with an average age of 47. Further demographics can be found in supplemental information section 11.
Data collection	For the Add Health data, waves 1-4 were administered by an in-home interviewer, while wave 5 collected responses via web and mail, with phone and in-person follow-ups for those who did not respond. Trained interviewers also took blood and urine samples and measured height and weight. Precise methods varied in each wave. As we obtained this data later from Add Health, collectors were blind to our hypotheses.<br><br>The CHIP50 survey was administered online, and respondents took the survey independently in the setting and time of their choice.
Timing	The Add Health survey began in 1994, with additional waves of data collection in 1996, 2001-2, 2008, and 2016-18. The CHIP50 survey was collected between 4/2/2024 and 5/6/2024.
Data exclusions	Respondents were excluded from the Add Health analyses only if they were missing responses to variables necessary for the analysis; the exact variables required for inclusion in each analysis are described in the Methods section. We did not pre-establish any exclusions.<br><br>The CHIP50 survey administrators excluded respondents who failed one of several checks for response quality, based on concerns that have arisen in recent years about the attentiveness and automation of online survey responses. These include failing a CAPTCHA test, failing simple attention check questions, and giving nonsensical responses to multiple questions. These criteria apply across CHIP50 studies (i.e. were pre-established before our study), and these decisions were not made by us or with any reference to responses to our survey questions of interest. Respondents were only excluded from our analyses if they were missing responses to variables necessary for the analysis.
Non-participation	In Add Health, 70% of selected schools agreed to participate in the survey, and 70-90% of the sample persisted through each wave in the survey. We do not have information about the causes of schools opting out of the initial survey. Attrition in subsequent waves, as is common across panel surveys, could result from changes in contact information or a loss of interest; Add Health reports differentially high attrition in lower-socioeconomic status groups, though they report the magnitude of this bias to be small.<br><br>In CHIP50, about 15-20% of respondents who start the survey do not finish it in typical waves. We do not retain information about the respondents who opt out of the survey, so we cannot make claims about differential attrition, though post-stratification weights and quotas ensure the sample of complete responses match population characteristics on observable traits.
Randomization	This study did not include randomization. We make only descriptive claims in the study, and while we sometimes control for covariates as a way of establishing that political beliefs are predictive above and beyond those covariates, or use those covariates to understand changes in coalitions, we do not use them to make causal claims.

Reporting for specific materials, systems and methods

We require information from authors about some types of materials, experimental systems and methods used in many studies. Here, indicate whether each material, system or method listed is relevant to your study. If you are not sure if a list item applies to your research, read the appropriate section before selecting a response.

Materials & experimental systems

n/a	Involved in the study	
☒	☐	Antibodies
☒	☐	Eukaryotic cell lines
☒	☐	Palaeontology and archaeology
☒	☐	Animals and other organisms
☒	☐	Clinical data
☒	☐	Dual use research of concern
☒	☐	Plants

Methods

n/a	Involved in the study	
☒	☐	ChIP-seq
☒	☐	Flow cytometry
☒	☐	MRI-based neuroimaging

Plants

Seed stocks

Report on the source of all seed stocks or other plant material used. If applicable, state the seed stock centre and catalogue number. If plant specimens were collected from the field, describe the collection location, date and sampling procedures.

Novel plant genotypes

Describe the methods by which all novel plant genotypes were produced. This includes those generated by transgenic approaches, gene editing, chemical/radiation-based mutagenesis and hybridization. For transgenic lines, describe the transformation method, the number of independent lines analyzed and the generation upon which experiments were performed. For gene-edited lines, describe the editor used, the endogenous sequence targeted for editing, the targeting guide RNA sequence (if applicable) and how the editor was applied.

Authentication

Describe any authentication procedures for each seed stock used or novel genotype generated. Describe any experiments used to assess the effect of a mutation and, where applicable, how potential secondary effects (e.g. second site T-DNA insertions, mosiacism, off-target gene editing) were examined.
