# Supplementary material for: The political polarization of health outcomes in the USA
Source: Nat Hum Behav. 2026 May 14;10(7):1234–45. doi: 10.1038/s41562-026-02474-9 (PMC13388103; doi:10.1038/s41562-026-02474-9)
Supplement: Supplementary file 3 — Peer Review file [file 41562_2026_2474_MOESM3_ESM.pdf]

nature portfolio

Peer Review File

The Political Polarization of Health Outcomes in the United States

Corresponding Author: Dr Elizabeth Elder

Version 0:

Decision Letter:

24th April 2025

Dear Elizabeth,

Thank you agian for submitting your manuscript entitled "The Political Polarization of Health Outcomes". As mentioned in my earlier email, we are returning your manuscript to you in our system with this "revise" decision. This is purely an administrative step, and as soon as you submit the revised files (and assuming there are no questions to resolve), we will proceed with inviting peer reviewers.

We ask that you thoroughly complete the Reporting Summary and Editorial Policy Checklist forms, but please note that we do not require any major formatting changes to your manuscript at this time. As long as information on ethics review/approval, data availability, and competing interests are provided, please do not feel obligated to make any other formatting changes at this stage.

When you are ready, please use the link below to resubmit:

Link Redacted

As soon as we receive these revised files, we will proceed with reviewer invitations. In the meantime, if you have any questions, please don't hesitate to let me know.

Sincerely,
[redacted]

[redacted]
Nature Human Behaviour

Version 1:

Decision Letter:

17th June 2025

Dear Dr. Elder,

Thank you once again for your manuscript, entitled "The Political Polarization of Health Outcomes," and for your patience during the peer review process.

Your manuscript has now been evaluated by 2 reviewers, whose comments are included at the end of this letter. Although the reviewers find your work to be of interest, they also raise some important concerns. We are interested in the possibility of publishing your study in Nature Human Behaviour, but would like to consider your response to these concerns in the form of a revised manuscript before we make a decision on publication.

To guide the scope of the revisions, the editors discuss the referee reports in detail within the team, including with the chief editor, with a view to (1) identifying key priorities that should be addressed in revision and (2) overruling referee requests that are deemed beyond the scope of the current study. We hope that you will find the prioritised set of referee points to be useful when revising your study. Please do not hesitate to get in touch if you would like to discuss these issues further.

In particular, please ensure that you address the following (as well as all other reviewer comments):

1) Address interpretational concerns raised by Reviewer 1.

2) Carry out the additional analyses recommended by Reviewer 1, including reporting annual mortality rates in addition to cumulative mortality.

3) Ensure that the details of your dataset and methodological approach are clear, and address concerns about the clarity of figures.

4) Include discussion of additional existing literature, as recommended by Reviewer 2.

Finally, your revised manuscript must comply fully with our editorial policies and formatting requirements. Failure to do so will result in your manuscript being returned to you, which will delay its consideration. To assist you in this process, I have attached a checklist that lists all of our requirements. Please pay particular attention to our requirements for reporting inferential statistical results and note that all results with $0.05 < p < 0.1$ must be interpreted as null unless an alpha level other than 0.05 was preregistered. If you have any questions about any of our policies or formatting, please don't hesitate to contact me.

In sum, we invite you to revise your manuscript taking into account all reviewer and editor comments. We are committed to providing a fair and constructive peer-review process. Do not hesitate to contact us if there are specific requests from the reviewers that you believe are technically impossible or unlikely to yield a meaningful outcome.

We hope to receive your revised manuscript within three months. I would be grateful if you could contact us as soon as possible if you foresee difficulties with meeting this target resubmission date.

With your revision, please:

• Include a “Response to the editors and reviewers” document detailing, point-by-point, how you addressed each editor and referee comment. If no action was taken to address a point, you must provide a compelling argument. When formatting this document, please respond to each reviewer comment individually, including the full text of the reviewer comment verbatim followed by your response to the individual point. This response will be used by the editors to evaluate your revision and sent back to the reviewers along with the revised manuscript.

• Highlight all changes made to your manuscript or provide us with a version that tracks changes.

• EXTENDED DATA FIGURES
When re-submitting your manuscript, please ensure that any supplementary figures and tables that are crucial to the manuscript's conclusions are converted into Extended Data figures and tables to increase visibility of these data. Extended Data figures and tables are online-only (present in the online PDF and full-text HTML versions of the paper), peer-reviewed display items that provide essential background to the article but are not included in the main article due to space constraints. A maximum of ten Extended Data display items (figures and tables) is permitted.

Please use the link below to submit your revised manuscript and related files:

Link Redacted

Note: This URL links to your confidential home page and associated information about manuscripts you may have submitted, or that you are reviewing for us. If you wish to forward this email to co-authors, please delete the link to your homepage.

We look forward to seeing the revised manuscript and thank you for the opportunity to review your work. Please do not hesitate to contact me if you have any questions or would like to discuss these revisions further.

Sincerely,
[redacted]

[redacted]
Nature Human Behaviour

Reviewer expertise:

Reviewer #1: health economics, public health

Reviewer #2: public policy, politics and health

REVIEWER COMMENTS:

Reviewer #1 (Remarks to the Author):

Review of “The Political Polarization of Health Outcomes”

Increased polarization of health outcomes by voting patterns has been well-documented at the ecological (aggregate) level, with falling or stagnant health outcomes in more conservative areas of the country. Paradoxically, however, the individual-level association between political ideology and health has historically run in the opposite direction: people with worse self-rated health are more likely to vote Democrat. While this study does not “solve” this paradox, it uses novel data to provide novel insights into the individual-level relationship, which seems to be catching up with aggregate trends.

A major contribution of the paper is that it is the first contemporary study to conduct an individual-level, longitudinal analysis of political ideology and health using objectively measured health biomarkers including mortality. Study findings conform to prior literature which found an historical advantage for conservatives with respect to self-rated health. However, the study reports an important and novel result: the individual level association between politics and health has shifted in recent years – conservatives now have worse health and higher mortality than liberals. The authors show that this shift started prior to the COVID-19 pandemic (and accelerated during it), was associated with increased mortality due to non-COVID-19 conditions, and was associated (in a large, novel online survey they conducted) with lower trust and lower utilization of preventive medical care.

In the “MAHA” era, in which the HHS director has rallied skepticism of the medical establishment (perhaps warranted in some instances), this paper provides essential data underscoring the potential human costs of that skepticism and the ways in which political narratives can shape health behaviors and outcomes.

Major comments:

• The authors state the objective of the paper as “to understand the relationship between politics and health in recent decades” (middle of page 2). Can they be more specific about the research questions that guided the analysis and how those questions related to the prior literature (reviewed in the prior two paragraphs)? The questions are implied in the summary of the key findings. However, it would be helpful to state the theoretically motivated research questions guiding the analysis.

• Top of page 5 – Figure 1. In addition to panels A and B, it might be an effective visual to show (as Fig 1C) the changes from Wave 4 to Wave 5. It would also be helpful to know the sample size in these different buckets, as the wide CI suggests the very conservative group is a relatively small share of the sample.

• Regarding Figure 1, all the action is in the most conservative tail of the distribution. This is really interesting. But the authors’ interpretation of the findings departs from the data. For example, they write that “the most liberal respondents were the most healthy”, but liberals’ had similar health to all but the most conservative. Additionally, based on the Figure 1 distribution, it seems odd to group “conservative” with “very conservative”. The changes in this most conservative group are not really explored further in the paper.

• Figure 2 shows average health on the y axis against changes in political affiliation on the x (category) axis. Why have the authors included wave 5 health? If they are interested in how health shapes political affiliation, should they just use baseline (wave 4 health)? Also, can the authors show the opposite: change in health vs. wave 4 political affiliation? (to complement Fig 4 which focuses on mortality) I think there is more they can do to unpack (descriptively) whether the shift from wave 4 to 5 is a result of worsening health among conservatives or increased conservatism of people with poor health, or both.

• In Figure 4, the authors do not comment on the wave 5 data (red dots), but the data suggest that political ideology shifted in such a way that ATTENUATED the relationships between internal mortality and conservative politics (e.g. perhaps sick conservatives became liberal) and between external mortality and liberal politics (e.g. perhaps being exposed to opiod overdoses makes liberal people more conservative). Any potential explanation for this pattern in the data?

• In Figure 4, the Y axis title should specify “Pr(Dead|Conservative)” or ““Pr(Dead|Conservative)” - “Pr(Dead|Liberal)””, as the current label doesn’t indicate whether positive numbers reflect excess mortality for conservatives or liberals

• Figure 4 is both wonderful and a bit misleading due to the focus on cumulative probabilities of death. I understand the focus on cumulative probabilities of death because it keeps the denominator constant, eliminates issues of mortality selection over time, and enables hypothesis testing with higher power. However, the focus on cumulative death makes it difficult for the reader to accurately pinpoint the moment when the relationship changes, as it looks like the relationship remains negative even after it has already turned positive. Specifically: whereas the design of Figure 4 suggests a focus on the statistically significant differences after 2020, if the authors plotted year-on-year (non-cumulative) death rates, it would be clear that the emergence of a conservative health disadvantage starts in 2015 (which incidentally coincides with the first Trump campaign and the nationalization of anti-institutional populism). In sum, I think it would be helpful if the authors could show annual mortality rates in addition to the cumulative.

• The authors deep dive on the COVID-19 period is welcome – because they have additional survey data on that period, because the politicization of health behavior during COVID is widely known, and because it is a nice contribution to show that it extends beyond COVID-related outcomes. However, related to the prior comment, I worry that the focus on the pandemic era distorts the reader’s attention from the root causes of the trends the authors are illuminating. E.g., the authors write (p10, bottom): “there is reason to believe that at least the period since 2020 has changed the relationship between politics and health in the population at large.” They also write (top of p16) “the initial decline in conservatives’ health… reflected a reshuffling of less healthy people between political coalitions”. But if my reading of their data is correct, these statements are not accurate. Based on Figure 4, it looks like the relationship between politics and health changed prior to COVID (starting around 2015 with the first Trump presidency) and the pandemic illustrated the deadly consequences of this change.

• Table 1 – in contrast to Figure 1 – shows a more or less continuous gradient between political ideology and mortality risk during COVID. This difference from Figure 1 is interesting but is hardly remarked on. As a companion to Figure 4, it would be very compelling to show 5-level individual-level ideology-mortality risk gradients for different time periods – not just 2020-2022 – and to present these as a figure (perhaps adjusted for age and sex).

• In Table 1, birth year (age) is included as a continuous variable. Is it appropriate to include age as a simple linear term? Are the findings robust to other specifications?

• On p13, the authors should make more of a meal about controlling for county FE. Most of the compelling prior results are at the county level, so these results are based on completely different source of variation in the data. This could be highlighted for the reader.

• The original survey needs a couple sentences of explanation in the main text (p13), to underscore that it was a large online sample w/sample size X and post-stratification weights to match national demographics. (Or some such.) Details can be provided in the methods and materials, but the reader needs some orientation.

• Figure 5 – the legend was rather confusing as the abbreviations are not defined. The figure needs a detailed explanatory note that

explains each of the series in the legend.
• In Figure 5, it is a bit inconvenient that the political ideology results are much less clean than the voting intention results. It is hard to know what to make of this. The authors emphasize the voting intention results (Trump v Biden), but the rest of the paper focuses on ideology. While these data help advance a hypothesis about loss of trust, in some ways these data open new challenges, making it harder to interpret what comes beforehand. It would be helpful to see (in the Appendix) some data on the correlation between voting intentions, partisanship, and ideology, how these concepts are distinct, and some reflection on what exactly "ideology" is measuring in the main paper results.
• In summarizing the results of their survey analysis, the authors should be a bit more circumspect. They write: "Taken together, these results suggest that right-leaning Americans—as measured by their vote choice, partisanship, and to a lesser extent their ideology—are less willing to visit, trust, and adhere to the advice of their primary care providers." This is true and is an important finding. However, it is hard to evaluate the significance of these findings as an explanation of their primary results, as these findings are not presented alongside alternate hypotheses and the data come from a period several years after the main change that they study.
• Can the authors do more to test whether their "trust" hypothesis is a plausible explanation for the observed trends? Could they assess whether trust has been a predictor of care utilization and health outcomes over time? Could they assess whether trust has diverged for liberals and conservatives and when this happened? In particular, what happened around 2015 that initiated this pattern?
• Can the authors comment more on the shift of less educated people to the conservative coalition. Is this a different causal pathway and potential confounder to control for? Is it constitutive of the same causal pathway? Just thinking outload, but perhaps there is an interaction between education and politics. Perhaps it's not [just] that less educated people with worse health outcomes shift to Republicans. It's that there's an interaction which makes the education gradient much steeper. Liberals favor public health paternalism (and redistributive policies), which protects less educated people from poor health decisions and from structural inequities. Conservatives favor more libertine policies, which leave less educated people to suffer the consequences of their choices and of local policy regimes.

The above comments reflect my belief that this paper makes a strong contribution on an important topic. The authors may decide that some of the additional analyses proposed are out of scope. However, I believe they have the opportunity to tie the pieces of the paper more closely together by addressing these comments.

Reviewer #2 (Remarks to the Author):

Dear Authors and Editors,
Thank you for inviting me to review this phenomenal manuscript. I believe this article should be published with haste. The authors have conducted an extremely rigorous analysis, employing multiple complementary approaches to answer the pressing question of whether and why the health of conservatives is worsening over time (and not just during/post-COVID).
The following are what I see as its particular strengths:
- Answering a novel and intractable question with improved data. As the authors note, there has been rising attention, especially since COVID (but also predating COVID in relation to the opioid crisis), to the relationship between political identity and health outcomes. This literature has suffered from numerous methodological challenges that the authors outline. The authors employ a panel dataset following the same individuals over time, which enables them to establish a high degree of internal validity.
- The authors examine the effects using biomarkers and mortality data and establish biologically plausible mechanisms for the observed effects. Many previous studies on this topic have, by contrast, relied on limited self-reported health data or are limited to COVID-19 related outcomes.
- The authors also make a substantively valuable contribution by examining the magnitude of the effects of political variables, demonstrating that the effects they observe are substantively important.
- The authors are forthright about the limitations of the AddHealth variables on political ideology and they complement their analysis with a large-N original survey that permits them to use alternative measures of their exposure variable (Party ID; vote choice). This is important, because ideology is often found to have low overlap with Party Identification and each may suggest different pathways of influence.
- The authors offer evidence of a potential mechanism driving worsening health among conservatives in the form of low trust in the health care system and consequent effects on health seeking behaviors.
- This last point is particularly important because the exact mechanisms explaining these growing health gaps have continued to be a source of debate with little clear evidence to support different pathways. This finding also appears to comport with the recent growth of the conservative MAHA movement where MAHA followers increasing reject "mainstream" allopathic medicine and appear to prefer increasing non-allopathic remedies.
I have little to suggest to the authors by way of improvement. However, to pick up on the latter point, if the authors were to consider revising anything, I think they might enhance their discussion with some of the literature that suggests that liberals may have also changed their health views (to be more pro-science). See for example:

• Motta, M. (2021). Republicans, Not Democrats, Are More Likely to Endorse Anti-Vaccine Misinformation. American Politics Research, 49(5), 428-438. https://doi.org/10.1177/1532673X211022639 (Original work published 2021)

Additionally, the authors might consider looking at this paper, which found some similar findings in regards to low(er) trust in physicians among Trump supporters:
• Choi, Y., & Fox, A. M. (2022). Mistrust in public health institutions is a stronger predictor of vaccine hesitancy and uptake than Trust in Trump. Social Science & Medicine, 314, 115440.

Version 2:

Decision Letter:

7th August 2025

Dear Elizabeth,

RE: "The Political Polarization of Health Outcomes"

Thank you for submitting your revised manuscript and for all your work on the revision. Thank you also for your patience while waiting for an update. I'm very sorry for the delay in getting back to you about your revision.

Although your manuscript has been revised in response to reviewer comments, it does not fully comply with our editorial policies and formatting requirements. Although we do not require that initial submissions follow our formatting guidelines, we do ask that post-review revised manuscripts adhere to the key points, in order to ensure that reviewers have the opportunity to evaluate a version of your manuscript that is close to what a final form would look like.

Before we can send the manuscript back to our reviewers, we ask that you revise it to ensure that it complies fully with our policies and is formatted according to our requirements. To assist with this process, I have attached another copy of our checklist. At this stage, we do not require that you address every detail (for instance, more minor points about the formatting of titles, references, and word limits are not essential at this stage). However, we do ask that you revise to address the following key points:

1) Please ensure that no claims are made based on marginally significant or non-significant results. For example, when interpreting columns 2 & 3 of Table 2 (lines 213-217), the manuscript states that the earlier pattern of results (increased mortality among the very conservative group) persists when separating the analysis by cause of death. However, column 2 shows a non-significant finding for very conservative indivdiuals (p=0.059), and column 3 shows non-significant coefficients for all groups. In another instance, line 224 states "race, education, and income predict mortality", but the relevant table (Column 4, Table 2) shows a p-value of 0.058 for education. Throughout the text, please carefully check to ensure that interpretations of effects/differences are not based on results that are not statistically significant.

2) For all interpretation of the difference between effect sizes, please ensure that the discussion is supported by a formal statistical test. For instance, if I am understanding correctly, lines 106 to 109 compare the effects of political views and education without a formal statistical test of the difference between these two effects. We would ask that you support this and any similar comparisons with a formal statistical test of the difference in effects.

3) Throughout the text, please ensure that the correlational nature of the evidence is entirely clear, and that no language is used that implies causality or directional effects.

If you are uncertain as to how to address these points, please don't hesitate to contact me.

Please use the link below to submit your revised manuscript and related files:

Link Redacted

Note: This URL links to your confidential home page and associated information about manuscripts you may have submitted, or that you are reviewing for us. If you wish to forward this email to co-authors, please delete the link to your homepage.

Thank you in advance for attending to these requests and I look forward to receiving your revised manuscript.

Sincerely,
[redacted]

[redacted]
Nature Human Behaviour

Version 3:

Decision Letter:

25th September 2025

Dear Dr. Elder,

Thank you once again for your revised manuscript, entitled "The Political Polarization of Health Outcomes," and for your patience during the re-review process.

Your manuscript has now been evaluated by Reviewer 1 from the original round of review. Their feedback is included at the end of this letter. Although the reviewer found your manuscript to have improved during revision, they also raise some important outstanding concerns. We remain very interested in the possibility of publishing your study in Nature Human Behaviour, but would like to consider your response to these outstanding concerns in the form of a revised manuscript before we make a decision on publication.

In particular, we would ask that you revise to address the reviewer's ongoing concerns about the presentation and interpretation of cumulative mortality rates. Please ensure that these results are clearly and accurately presented. As you revise, please also address all other comments from Reviewer 1.

Finally, your revised manuscript must comply fully with our editorial policies and formatting requirements. Failure to do so will result in your manuscript being returned to you, which will delay its consideration. To assist you in this process, I have attached a copy of our checklist that lists all of our requirements. We would ask that you particularly revise to ensure that no causal language is used or implied when interpreting correlational results and that p-values larger than 0.05 are not marked or interpreted as statistically significant. If you have any questions about any of our policies or formatting, please don't hesitate to contact me.

In sum, we invite you to revise your manuscript taking into account all reviewer and editor comments. We are committed to providing a fair and constructive peer-review process. Do not hesitate to contact us if there are specific requests from the reviewers that you believe are technically impossible or unlikely to yield a meaningful outcome.

We hope to receive your revised manuscript within 4-8 weeks. I would be grateful if you could contact us as soon as possible if you foresee difficulties with meeting this target resubmission date.

With your revision, please:

• Include a "Response to the editors and reviewers" document detailing, point-by-point, how you addressed each editor and referee comment. If no action was taken to address a point, you must provide a compelling argument. This response will be used by the editors and reviewers to evaluate your revision.

• Highlight all changes made to your manuscript or provide us with a version that tracks changes.

Please use the link below to submit your revised manuscript and related files:

Link Redacted

Note: This URL links to your confidential home page and associated information about manuscripts you may have submitted, or that you are reviewing for us. If you wish to forward this email to co-authors, please delete the link to your homepage.

We look forward to seeing the revised manuscript and thank you for the opportunity to review your work. Please do not hesitate to contact me if you have any questions or would like to discuss these revisions further.

Sincerely,
[redacted]

[redacted]
Nature Human Behaviour

Reviewer expertise:

Reviewer #1: health economics, public health

Reviewer #2: Did not re-review

REVIEWER COMMENTS:

Reviewer #1 (Remarks to the Author):

Thank you for the opportunity to review this R&R.

The authors have been highly responsive to my original critiques. I continue to believe that this paper makes an important and novel contribution to the literature on political ideology as a social determinant of health. The link between conservative ideology and poor health emerged in public view during the COVID-19 pandemic. The authors show that this relationship had already emerged (starting in 2016) and that it was present not just for COVID-19 related deaths but for other internal causes of death during the pandemic. Further, they show that the correlation between political ideology and health outcomes is NOT mediated by place (county), and advance "trust in the health system" as a plausible explanation. Overall, I found the revision to be clear, careful, and responsive.
A few specifics.
• The new paragraph "This paper explores three questions…" is very effective at setting the stage and clarifies the paper's contributions. Thank you.
• Please specify "which time period" and consider a relative measure of association given the low background death rates in the sentence (p3): "The divide since 2020 is substantial: people who 56 identify as "very conservative" are 1.06 percentage points (two-

tailed t-test; p = .046; CI: .01,2.11) more likely to die in this time period compared to those who identify as "very liberal"…
• I still find the choice to show Fig 4 as cumulative mortality to be misleading, as it focuses attention on later years (COVID) rather than earlier when the emergence of the conservative disadvantage occurs. The supplementary figure Fig A4 shows a pretty clear 2016 discontinuity in excess (internal) death rate among conservatives. In contrast to the data, the interpretation appears to downplay the 2016 discontinuity. On p9, the authors write "However, around 2015, this began to change, and deaths accumulated more rapidly among those on the political right." The phrase "deaths accumulated more rapidly" is a pretty round about way of saying that there was a level shift increase in internal causes mortality on the political right between 2015 and 2016, which has persisted to this day. Further, the authors state that "the relationship between politics and mortality became consistently significant only after 2020." That is true only because they are making inferences on cumulative mortality, and by 2020 enough years of have passed for excess mortality to accumulate to levels of "significance". The data and hypothesis tests shown in the cumulative plot do not correspond to how most readers will interpret that figure, because each annual data point includes all prior years' data. If the authors wish to show this as a cumulative plot then it would make more sense to show respondent age or time since survey as the time-scale rather than year, which gives the impression of a period analysis rather than a cohort analysis. Further note: I appreciated the additional regression analyses, looking at 3-year periods, in the appendix. I do think it would be good to assess whether there was an intercept shift in the ideology-mortality relationship in 2016. However, there seems to be an inconsistency in the results between Figure A4 and Table A10. The coef for very conservative is 0.01 in 2017-2019 and 0.79 in 2020-22. But looking at Fig A4 (for Wave 4), the coefs look qualitatively of similar magnitude. Perhaps the authors can help reconcile this.
• It is frankly shocking to me that the county FE have such a small impact on the political ideology – mortality relationship during COVID-19 (Table 2). We spend so much time talking about how "place matters" and focusing on state and local policy… This is a profoundly important finding that turns the field of social epidemiology a bit on its head. Bravo.
• Figure 5 – I still find this figure quite confusing. Even with the changes, it took me quite a while to make sense out of the legend and understand what the different data series were. Additionally, this is the first time the authors introduce different political exposure variables. I think it would be clearer to drop the first two columns and just show ideology, for a cleaner comparison with the rest of the paper. (The full figure could be an appendix figure.) In addition, I'd suggest showing just ONE data point for each outcome (probably the grey multivariate dots) and drop the rest. The bivariate relationships are not particularly interesting and the results for respondents with chronic diseases are pretty similar to the general population. These suggestions are designed to streamline the figure so that it highlights the main result: conservatives have less trust in the health system.
• I love the new public opinion data added to the response letter on historical patterns in health screenings and trust. Thank you for sharing this. It's too bad the data aren't more detailed, but I do find Fig 1 in the response letter to be compelling and potentially a worthwhile addition to the supplement, if only to corroborate your results in national samples.

Version 4:

Decision Letter:

Our ref: NATHUMBEHAV-25041648D

22nd January 2026

Dear Elizabeth,

Thank you again for submitting your revised manuscript "The Political Polarization of Health Outcomes" (NATHUMBEHAV-25041648D), and for your patience while waiting for a decision. I'm very sorry for the long wait you have experienced.

Your revised manuscript has now been seen by one of the original referees, and their comments are below. As you can see, the reviewer finds that the paper has improved in revision. We will therefore be happy in principle to publish it in Nature Human Behaviour, pending minor revisions to comply with our editorial and formatting guidelines.

We are now performing detailed checks on your paper and will send you a checklist detailing our editorial and formatting requirements within two weeks. Please do not upload the final materials and make any revisions until you receive this additional information from us.

Please do not hesitate to contact me if you have any questions.

Sincerely,
[redacted]

[redacted]
Nature Human Behaviour

Reviewer #1 (Remarks to the Author):

Thank you for the opportunity to review this second revision. The authors have satisfactorily addressed all of my concerns.

Kudos to the authorship team on an excellent contribution.

Version 5:

Decision Letter:

Dear Elizabeth,

I am happy to let you know that your Article "The Political Polarization of Health Outcomes in the United States", has now been accepted for publication in Nature Human Behaviour.

Authors may need to take specific actions to achieve compliance with funder and institutional open access mandates. If your research is supported by a funder that requires immediate open access (e.g. according to Plan S principles or the NIH public access policy) then you should select the gold OA route, and we will direct you to the compliant route where possible. Because authors warrant under our subscription licensing terms that they haven't committed to licensing any version of their article under a licence inconsistent with the terms of our agreement – including the applicable embargo period – publication under the subscription model isn't suitable for authors whose funders require no embargo.

Once your manuscript is typeset and you have completed the appropriate grant of rights, you will receive a link to your electronic proof via email with a request to make any corrections within 48 hours. If, when you receive your proof, you cannot meet this deadline, please inform us at rjsproduction@springernature.com immediately. Once your paper has been scheduled for online publication, the Nature press office will be in touch to confirm the details.

Acceptance of your manuscript is conditional on all authors' agreement with our publication policies (see http://www.nature.com/nathumbehav/info/gta). In particular your manuscript must not be published elsewhere and there must be no announcement of the work to any media outlet until the publication date (the day on which it is uploaded onto our web site).

If you have posted a preprint on any preprint server, please ensure that the preprint details are updated with a publication reference, including the DOI and a URL to the published version of the article on the journal website.

An online order form for reprints of your paper is available at https://www.nature.com/reprints/author-reprints.html. All co-authors, authors' institutions and authors' funding agencies can order reprints using the form appropriate to their geographical region.

We welcome the submission of potential cover material (including a short caption of around 40 words) related to your manuscript; suggestions should be sent to Nature Human Behaviour as electronic files (the image should be 300 dpi at 210 x 297 mm in either TIFF or JPEG format). Please note that such pictures should be selected more for their aesthetic appeal than for their scientific content, and that colour images work better than black and white or grayscale images. Please do not try to design a cover with the Nature Human Behaviour logo etc., and please do not submit composites of images related to your work. I am sure you will understand that we cannot make any promise as to whether any of your suggestions might be selected for the cover of the journal.

You can now use a single sign-on for all your accounts, view the status of all your manuscript submissions and reviews, access usage statistics for your published articles and download a record of your refereeing activity for the Nature journals.

To assist our authors in disseminating their research to the broader community, our SharedIt initiative provides you with a unique shareable link that will allow anyone (with or without a subscription) to read the published article. Recipients of the link with a subscription will also be able to download and print the PDF.

As soon as your article is published, you will receive an automated email with your shareable link.

In approximately 10 business days you will receive an email with a link to choose the appropriate publishing options for your paper and our Author Services team will be in touch regarding any additional information that may be required.

If you have any questions about our publishing options, costs, Open Access requirements, or our legal forms, please contact ASJournals@springernature.com

We look forward to publishing your paper.

With best regards,
[redacted]

[redacted]
Nature Human Behaviour

P.S. Click on the following link if you would like to recommend Nature Human Behaviour to your librarian
http://www.nature.com/subscriptions/recommend.html#forms

** Visit the Springer Nature Editorial and Publishing website at www.springernature.com/editorial-and-publishing-jobs for more information about our career opportunities. If you have any questions please click here.**

Open Access This Peer Review File is licensed under a Creative Commons Attribution 4.0 International License, which permits use, sharing, adaptation, distribution and reproduction in any medium or format, as long as you give appropriate credit to the original author(s) and the source, provide a link to the Creative Commons license, and indicate if changes were made.
In cases where reviewers are anonymous, credit should be given to 'Anonymous Referee' and the source.
The images or other third party material in this Peer Review File are included in the article's Creative Commons license, unless indicated otherwise in a credit line to the material. If material is not included in the article's Creative Commons license and your intended use is not permitted by statutory regulation or exceeds the permitted use, you will need to obtain permission directly from the copyright holder.
To view a copy of this license, visit https://creativecommons.org/licenses/by/4.0/

Responses to Reviewer 1

The reviewer requests a more explicit statement of the motivating research questions and how these questions relate to prior literature and to our analysis. They write: *"The authors state the objective of the paper as "to understand the relationship between politics and health in recent decades" (middle of page 2). Can they be more specific about the research questions that guided the analysis and how those questions related to the prior literature (reviewed in the prior two paragraphs)? The questions are implied in the summary of the key findings. However, it would be helpful to state the theoretically motivated research questions guiding the analysis."*

- We thank the reviewer for prompting us to clearly state our research questions. We now do so in a new paragraph on page 2, which reads:
  - This paper explores three questions. First, we seek to reconcile the existing mixed findings on the relationship between politics and health using longitudinal, individual-level data. Second, we ask how this relationship has changed since 2010, when previous mortality analyses conclude. And finally, we seek potential explanations for these changes, identifying promising candidates for future research.

The reviewer suggests that figure 1 directly show change in health outcomes over time, as well as label the sample sizes of each group. They write: *"Top of page 5 – Figure 1. In addition to panels A and B, it might be an effective visual to show (as Fig 1C) the changes from Wave 4 to Wave 5. It would also be helpful to know the sample size in these different buckets, as the wide CI suggests the very conservative group is a relatively small share of the sample."*

- We appreciate the viewers' suggestions to improve figure 1. We have added Ns to the bottom of the plot as suggested. Rather than adding a panel showing the changes between wave 4 to wave 5, the revised manuscript now includes a table following Figure 1 (Table 1 in revised manuscript) showing the difference between the cross-sectional differences in health by ideology, between waves 4 and 5, which allows for a clearer reporting of the required statistical tests.

The reviewer points out that in Figure 1, the "very conservative" group stands out, while other groups are mostly similar. They suggest the interpretation of the figure reflect this and that this group be investigated further in the paper's other analyses. They write: *"Regarding Figure 1, all the action is in the most conservative tail of the distribution. This is really interesting. But the authors' interpretation of the findings departs from the data. For example, they write that "the most liberal respondents were the most healthy", but liberals had similar health to all but the most conservative. Additionally, based on the Figure 1 distribution, it seems odd to group "conservative" with "very conservative". The changes in this most conservative group are not really explored further in the paper."*

- The reviewer raises an important point: the "very conservative" group is both the most different from other ideological groups and shows the largest change over time, while the other groups show only small changes. We have edited the language on page 5 describing these findings to align with this.

- We agree that these findings are interesting, but we are limited in our ability to explore them further: the relatively small size of the "very conservative" group (especially when limiting to respondents with complete data in both waves) prevents us from breaking out these respondents from other conservatives in figure 2, which also subdivides respondents by their patterns of ideological change. We have added a figure to the appendix (section 2, figure A2) that shows biomarkers among conservative and very conservative respondents based on their patterns of change (where allowable given privacy constraints of the Add Health data); while these analyses suggest there are not hugely different patterns between conservative and very conservative respondents, the small sample sizes prevent us from drawing strong conclusions. A footnote on page 6 in the main text notes this finding.

- As the reviewer notes in another comment, the mortality analyses do not show this pattern: in most models, all ideological groups (except liberals) have significantly higher mortality than the very liberal in 2020-2022. We have therefore not chosen to highlight the distinctiveness of the very conservative in our overall characterizations of the papers' findings in the introduction and conclusion.

The reviewer requests greater exploration of the changes in biomarkers shown in figure 2. They write: *"Figure 2 shows average health on the y axis against changes in political affiliation on the x (category) axis. Why have the authors included wave 5 health? If they are interested in how health shapes political affiliation, should they just use baseline (wave 4 health)? Also, can the authors show the opposite: change in health vs. wave 4 political affiliation? (to complement Fig 4 which focuses on mortality) I think there is more they can do to unpack (descriptively) whether the shift from wave 4 to 5 is a result of worsening health among conservatives or increased conservatism of people with poor health, or both."*

- The revised manuscript makes an effort to clarify Figure 2, which we think does what the reviewer suggests: Figure 2 shows both wave 4 health (in white) and wave 5 health (in gray), and the panels sort respondents based on their ideology in *both* waves. Each panel includes the same set of respondents in both waves. So, the reader can see that respondents who would become more conservative (or liberal) by wave 5 were already slightly less (more) healthy than other respondents in wave 4.

- Because waves are spaced fairly far apart, we cannot precisely identify when respondents' health declined or when their ideology changed, so we cannot say whether (for example) that the group that became conservative was less healthy before or after they became conservative—only that both happened between 2008 (when those who would become more conservative were only slightly less healthy) and 2016 (when they had become both conservative and much less healthy).

- We have revised the language around Figure 2 to clarify its features and its takeaway on page 6.
- We have also added a table to the appendix regressing wave 5 health outcomes on wave 4 ideology, as well as the reverse—regressing wave 5 ideology on wave 4 health outcomes—in SA 1 (Table A5). The data are consistent with the hypothesis that less healthy people in wave 4 are moving to the right over this time frame (although the relationship loses statistical significance when adding controls); wave 4 ideology does not predict change in health over this time-frame. We have added a footnote noting this on page 7 and updated the interpretation of Figure 2 to reflect these findings on page 6-7.
- Should the manuscript move forward, we are happy to work with the editor/reviewer to further update the figure or add detail if we have misunderstood the reviewer's request.

The reviewer points out an interesting pattern in the relationship between mortality and ideology as measured in different waves. They write: *"In Figure 4, the authors do not comment on the wave 5 data (red dots), but the data suggest that political ideology shifted in such a way that ATTENUATED the relationships between internal mortality and conservative politics (e.g. perhaps sick conservatives became liberal) and between external mortality and liberal politics (e.g. perhaps being exposed to opiod overdoses makes liberal people more conservative). Any potential explanation for this pattern in the data?"*

- We agree with the reviewer that this is a notable pattern. To offer a different interpretation, though, the wave 5 estimates are closer to zero for both internal and external causes of death in the initial years after the wave 5 measures were taken. Wave 4 ideology also showed weaker relationships with all three outcomes in the first years after it was taken, then grew closer to wave 3 measures over time. It could be that there is a lag between expressing an ideological position and its translation into mortality—perhaps, for example, if it takes time for ideologically-related loss of trust in medicine to manifest in receiving less care.
- Because the more recent years of the wave 5 measure overlap with the COVID-19 pandemic, we cannot say for sure whether any differences in its relationships are due to maturation of the measure or changes in the political context, so we hesitate to address this in the manuscript.
- We also take the reviewer's point that, if anything, conservative ideology in wave 5 appears to be moving towards predicting *higher* externally-caused deaths, rather than the opposite, as was the case in the past. Because these results are not significant, or significantly different from past years, though, we cannot draw strong conclusions about this. Future years of death data may shed further light upon this.

The reviewer requests a clarified label on figure 4: *"In Figure 4, the Y axis title should specify "Pr(Dead—Conservative)" or ""Pr(Dead—Conservative)" - "Pr(Dead—Liberal)"", as the current label doesn't indicate whether positive numbers reflect excess mortality for conservatives or liberals."*

- We appreciate the reviewer's suggestion and have updated the label on figure 4 to clarify this.

The reviewer, though they recognize the advantages of looking at cumulative mortality over time, argues that showing year-by-year mortality would more clearly show the timing of the emerging health gap. They write: *"...the focus on cumulative death makes it difficult for the reader to accurately pinpoint the moment when the relationship changes, as it looks like the relationship remains negative even after it has already turned positive. Specifically: whereas the design of Figure 4 suggests a focus on the statistically significant differences after 2020, if the authors plotted year-on-year (non-cumulative) death rates, it would be clear that the emergence of a conservative health disadvantage starts in 2015 (which incidentally coincides with the first Trump campaign and the nationalization of anti-institutional populism). In sum, I think it would be helpful if the authors could show annual mortality rates in addition to the cumulative."*

- We appreciate the reviewer's weighing of the merits of showing cumulative vs. year-by-year deaths. We ultimately opted to leave the cumulative analysis in the manuscript for reasons of power, but we have added figure A4 to section 4 in the appendix showing the relationship between death in any individual year and ideology. As the reviewer suspects, the year-to-year estimates are variable and not very precise, given the small numbers of death in each year. Broadly speaking, the coefficients are directionally consistent with conservatism predicting mortality begin to appear in 2016, but this is not consistent. We have added a footnote on page 9 referring to these findings.
- We have also added analyses to the appendix regressing mortality during several three-year periods on ideology and other variables, paralleling the 2020-22 analysis. This is included in SA 6. These analyses suggest the 2020-22 period is substantively different from 2017-2019 and, for wave 4 measures, 2014-16: the gaps in health present in 2020-2022 were not yet present in earlier periods.
- Based on this evidence, we have maintained our claim in the paper that the gap in mortality emerged only after 2020. One potential explanation for this timing is that the gaps in biomarkers observed in 2016-2018 were more immediate consequences of gaps in engagement with the medical system, while gaps in mortality took longer to manifest.

The reviewer points out it is not clear there was a qualitative change in the relationship between ideology and health after the COVID-19 pandemic rather than a continuation of preexisting trends. They write: *"...I worry that the focus on the pandemic era distorts the reader's attention from the root causes of the trends the authors are illuminating. E.g., the authors write (p10, bottom): "there is reason to believe that at least the period since 2020 has changed the relationship between politics and health in the population at large." They also write (top of p16) "the initial decline in conservatives' health... reflected a reshuffling of less healthy people between political coalitions". But if my reading of their data is correct, these statements are not accurate. Based on Figure 4, it looks like the relationship between politics and health changed prior to COVID (starting around 2015 with the first Trump presidency)*

and the pandemic illustrated the deadly consequences of this change."

- We thank the reviewer for raising this important point about the interpretation of our results. We have edited the manuscript in several places to bring our statements of the findings in closer line with the evidence. In the abstract and the discussion (page 18, formerly page 16 as noted by the reviewer), we have removed references to the timing of the sorting and trust mechanisms. And on page 11 (formerly 10), we have removed the reference to the notion that the pandemic changed the relationship between ideology and health.

- There is some evidence from other work on the timing of the political gap in trust: we have added citations to other work showing the gap in trust in medicine as an institution between left and right did not emerge until after the pandemic (page 14). However, we also refer there to work suggested by Reviewer 2 that shows some of the divides in health attitudes (e.g., flu vaccines) predate the pandemic. So, while the scope of the political divide has expanded since the pandemic, we cannot rule out that it played a role in the earlier gaps in health.

- The manuscript emphasizes, in its discussion of Figure 3, that the available evidence suggests that the health divide that emerged in the 2010s was at least partially a result of the changing demographics of who called themselves liberal and conservative, but around half the gap remains unexplained (page 9). This could be the result of gaps in engagement with the healthcare system that predated the pandemic, but as we note on page 15, we lack the data to test this directly.

- As we noted in our response to the previous question, the additional analyses breaking down mortality by year or three-year period suggest the political gap in mortality in fact did not emerge until after 2020, or at least was not at all consistent. As the reviewer points out, though, we cannot say whether the relationship between ideology and death would have grown even absent the pandemic's effects on trust in medicine. Our adjusted statements of our findings no longer claim that this mortality gap is due to that mechanism.

- We have also clarified in our discussion of our original survey that we cannot speak to the origins or timing of the gaps in the Add Health data, since the survey took place after it concluded–see page 15 and page 18.

Recalling the distinctiveness of "very conservative" respondents in figure 1, the reviewer asks about the relationship between individual ideology categories and health over time for the outcome of mortality: *"Table 1 – in contrast to Figure 1 – shows a more or less continuous gradient between political ideology and mortality risk during COVID. This difference from Figure 1 is interesting but is hardly remarked on. As a companion to Figure 4, it would be very compelling to show 5-level individual-level ideology-mortality risk gradients for different time periods – not just 2020-2022 – and to present these as a figure (perhaps adjusted for age and sex)."*

- This is an important distinction from the findings in figure 1, and we have added a sentence on page 11-12 remarking on this.
- As noted above, we have added tables to the appendix regressing mortality in three-year intervals on ideological categories, with and without controls (see SA section 6, table A9 and A10). As we also note above, though, mortality in periods before 2020-2022 had no relationship with any category of ideology. We have therefore opted not to highlight these results further with a figure.

The reviewer asks about alternative specifications of age in the regression models: *"In Table 1, birth year (age) is included as a continuous variable. Is it appropriate to include age as a simple linear term? Are the findings robust to other specifications?"*

- To address this question, SA 7 table A11 replicates what was Table 1 (now Table 2 in revised manuscript), but includes age as a dummy variable based on three-year birth periods (the respondents are born between 1974 and 1983) for maximum flexibility in the relationship between age and health. Results are robust to this specification.

The reviewer suggests we do more to emphasize the motivation and findings of the models with county fixed effects. They write: *"On p13, the authors should make more of a meal about controlling for county FE. Most of the compelling prior results are at the county level, so these results are based on completely different source of variation in the data. This could be highlighted for the reader."*

- We appreciate the reviewer prompting us to highlight these findings. We have added text to page 14 to motivate and emphasize the county fixed effects as the reviewer suggests.

The reviewer requests more explanation of the original survey in the main text, writing: *"The original survey needs a couple sentences of explanation in the main text (p13), to underscore that it was a large online sample w/sample size X and post-stratification weights to match national demographics. (Or some such.) Details can be provided in the methods and materials, but the reader needs some orientation."*

- We have added a footnote to page 15 providing a brief description of the survey sample and referring the reader to the relevant section of the Methods. We are mindful of the word limit, but we can add more details if the reviewer feels they are needed to orient the reader.

The reviewer requests a more detailed label/caption on figure 5, writing: *"Figure 5 – the legend was rather confusing as the abbreviations are not defined. The figure needs a detailed explanatory note that explains each of the series in the legend."*

- Figure 5 now includes an expanded legend that better explains each of the series. We also include phrasing in the main text that more clearly describes the different estimates, and the caption more clearly defines the labels on the y-axis.

The reviewer suggests more be done to unpack the finding that ideology is a weaker predictor of health attitudes and behaviors than other political variables. They write: *"In Figure 5, it is a bit inconvenient that the political ideology results are much less clean than the voting intention results. It is hard to know what to make of this. The authors emphasize the voting intention results (Trump v Biden), but the rest of the paper focuses on ideology. While these data help advance a hypothesis about loss of trust, in some ways these data open new challenges, making it harder to interpret what comes beforehand. It would be helpful to see (in the Appendix) some data on the correlation between voting intentions, partisanship, and ideology, how these concepts are distinct, and some reflection on what exactly "ideology" is measuring in the main paper results."*

- First, to address this empirically, the SA section 10 now includes cross-tabs of partisanship by ideology and vote choice by ideology. Most liberals consider themselves Democrats (about 83%) in these data and very few consider themselves Republicans (less than 5%). The tightening relationship between ideology, partisanship and vote choice in recent decades has been well documented (for example, see Grossman 2024).

  It is unclear why the relationship between trust and political attitudes is weaker for ideology than for vote choice or partisanship. One possibility is that when asked about their ideology, more people say they are moderate (i.e. do not choose liberal or conservative) when compared to people who say they are not attached to a party (i.e. do not choose Democrat or Republican). In our survey data, 20% of respondents call themselves Independent, but 39% call themselves moderate (tables A24 and A25). But most of these moderates have political preferences: 65% of moderates identify as Republicans or Democrats, and 64% indicated support for Trump or Biden (tables A26 and A27). The "moderate" ideological category may therefore conceal some variation in political attitudes that partisanship measures or vote choice can better capture.

- To explore these points, SA table A28 includes attitudes by vote choice, just among ideological moderates. Among moderates, vote choice is quite predictive of trust and engagement with the healthcare system. We note this in a footnote on page 16.

- Another possible explanation, and related to the point above, is that much of this division in trust is driven not by ideology in itself, but by elite cues around the response to COVID-19; Presidents Trump and Biden sent widely varying signals about the trustworthiness of public health measures during the pandemic. Vote choice between these two candidates (and its close cousin, partisanship) would then be a more direct measure of what's driving the change in trust. Ideology, though an increasingly good predictor of vote choice, would be an indirect proxy for the real causal mover. If this is the case, health outcomes may be even more polarized by vote choice or partisanship than by ideology—though we can only measure the latter here. We allude to this in the discussion on page 18.

The reviewer suggests the summary of the survey findings be more mindful of its limitations. They write: *"it is hard to evaluate the significance of these findings as an explanation of their primary results, as these findings are not presented alongside alternate hypotheses and the data come from a period several years after the main change that they study."*

- We appreciate this caution from the reviewer and have added a caveat on page 15 when introducing the survey and on page 18 when discussing the paper's limitations. We now note that because it took place in 2024, this evidence cannot speak directly to attitudes in earlier years, and we clarify its role as proposing and exploring a possible explanation.

Continuing from the above point, the reviewer asks whether more can be done to assess the role of trust in the health gap. They write: *"Can the authors do more to test whether their "trust" hypothesis is a plausible explanation for the observed trends? Could they assess whether trust has been a predictor of care utilization and health outcomes over time? Could they assess whether trust has diverged for liberals and conservatives and when this happened? In particular, what happened around 2015 that initiated this pattern?"*

- The revised manuscript cites prior work that gives at least a partial answer to the reviewer's question: a partisan gap in trust in medicine as an institution did not exist before 2020, but has since emerged (e.g., Blendon and Benson 2021, Brady and Kent 2022). There is unfortunately no comparable time-series data measuring trust in one's personal doctor, adherence to their advice, or belief in medicines. There is some evidence that liberals and conservatives exhibit some slightly different health behaviors (e.g., flu vaccine uptake) before the pandemic, which we now cite as well. We add a sentence on page 14 detailing these trends.

  The revised manuscript also includes a short paragraph in the conclusion on page 18 noting that the timing of our survey and the lack of trust measures on the Add Health survey prevent us from directly testing the timing and predictiveness of the trust gap and how it relates to health outcomes. We identify this as an important area for future work.

- In an effort to address this question, we looked further for data on health care utilization over time by political affiliation. We identified three surveys spanning the 2010s that asked slight variations on whether the respondent had "Gone to a health care provider for a general physical exam."[1] These surveys echo a finding we present (in Figure 5) regarding whether the respondent had seen their primary care provider in the last 12 months.

  In 2012 and 2016, Democrats and Republicans reported seeing their physician for an annual check-up at similar rates. In 2023, Republicans are 8.03 pp. less likely to report having had an annual physical screening ($\beta = -.0803$ two-tailed t-test; $p = .032$; CI: -.15,-.07). We present this graphic below. A similar pattern persists after controlling for available demographics (in 2023, with demographic controls: $\beta = -.0799$ two-tailed t-test; $p = .044$; CI: -.16,-.002).

  The 2023 Axios survey also asks respondents, "How much trust do you have, if any, in information about health topics from the following? ... Your personal doctor." We

[1] Pew asked this in 2016. RWJ asked in 2012 "In the past 12 months, have you seen a doctor or nurse practitioner for a routine check-up, or not?" And Axios in 2023 asked, "In the last year, have you had an annual health screening with a physician."

Figure 1: Had a physical/health screening in last 12 months

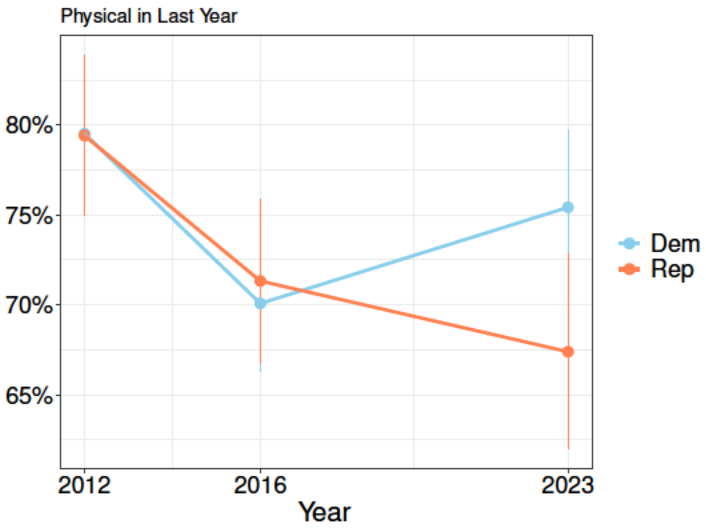

cross-tab the relationship between people's trust in information from their personal doctor with whether they reported having seen a doctor for an annual physical screening. A clear gradient emerges: 80% of respondents who report having "a great deal of trust" report having had an annual physical exam in the prior year compared to just 27% of respondents who say they have "None at all."

Table 1: Health screening in last 12 months by Trust Info From Personal Doctor

	None at all	Not very much	A fair amount	A great deal	Total
No	73.04	45.62	36.94	19.56	32.01
Yes	26.96	54.38	63.06	80.44	67.99
Total	100.00	100.00	100.00	100.00	100.00

We find these data interesting and supportive of the reviewer's notion that something changed between 2015 and 2024. However, they do not allow us to date the timing of the change more precisely, and we are hesitant to include another data source in the paper due to space limitations. We have therefore not included these data in the revised manuscript, instead relying on the added citations for the notion that a gap in engagement grew after COVID-19. However, if this is a sticking point for the manuscript moving forward, we are happy to work with the reviewer and editors to include it in the main text or appendix.

The reviewer asks for greater discussion of educational realignment and its relationship with health. They write: *"Can the authors comment more on the shift of less educated people to the conservative coalition. Is this a different causal pathway and potential confounder to control for? Is it constitutive of the same causal pathway? Just thinking out loud, but perhaps there is an interaction between education and politics. Perhaps it's not [just] that less educated*

people with worse health outcomes shift to Republicans. It's that there's an interaction which makes the education gradient much steeper. Liberals favor public health paternalism (and redistributive policies), which protects less educated people from poor health decisions and from structural inequities. Conservatives favor more libertine policies, which leave less educated people to suffer the consequences of their choices and of local policy regimes."

- We view the educational realignment as part of the causal pathway we explore in the decomposition analysis (figure 3): as liberals have become more educated and the income gap has eroded or reversed, the liberal coalition has become healthier. We have added a sentence to page 8 explicitly stating this as in-line with our proposed explanation.
- The reviewer also raises an interesting question about the interaction between individual partisanship and the aggregate effects of partisan control on health policy. We hope to explore this in future work, but we feel it is outside the scope of this manuscript.

Responses to Reviewer 2

The reviewer suggests we incorporate a discussion of the finding that liberals may have become more pro-medicine or pro-science as conservatives have become less trusting in these institutions. They write: *I think they might enhance their discussion with some of the literature that suggests that liberals may have also changed their health views (to be more pro-science).* They suggest a citation on this point. They also suggest a citation to earlier work showing differences in trust in medicine by party.

- We thank the reviewer for suggesting these additional references. We have added these citations both to the introduction on page 3, as support for the notion that right-leaning Americans are less engaged with the healthcare system. We have also added a sentence to page 14 noting that, as the reviewer points out, conservatives are not the only group changing their attitudes: liberals have become *more* trusting of scientific experts (also referring to their suggested citation). We appreciate these prompts to engage with these pieces.

Responses to Reviewer 1

The reviewer requests a more explicit statement of the motivating research questions and how these questions relate to prior literature and to our analysis. They write: *"The authors state the objective of the paper as "to understand the relationship between politics and health in recent decades" (middle of page 2). Can they be more specific about the research questions that guided the analysis and how those questions related to the prior literature (reviewed in the prior two paragraphs)? The questions are implied in the summary of the key findings. However, it would be helpful to state the theoretically motivated research questions guiding the analysis."*

- We thank the reviewer for prompting us to clearly state our research questions. We now do so in a new paragraph on page 2, which reads:
  - This paper explores three questions. First, we seek to reconcile the existing mixed findings on the relationship between politics and health using longitudinal, individual-level data. Second, we ask how this relationship has changed since 2010, when previous mortality analyses conclude. And finally, we seek potential explanations for these changes, identifying promising candidates for future research.

The reviewer suggests that figure 1 directly show change in health outcomes over time, as well as label the sample sizes of each group. They write: *"Top of page 5 – Figure 1. In addition to panels A and B, it might be an effective visual to show (as Fig 1C) the changes from Wave 4 to Wave 5. It would also be helpful to know the sample size in these different buckets, as the wide CI suggests the very conservative group is a relatively small share of the sample."*

- We appreciate the viewers' suggestions to improve figure 1. We have added Ns to the bottom of the plot as suggested. Rather than adding a panel showing the changes between wave 4 to wave 5, the revised manuscript now includes a table following Figure 1 (Table 1 in revised manuscript) showing the difference between the cross-sectional differences in health by ideology, between waves 4 and 5, which allows for a clearer reporting of the required statistical tests.

The reviewer points out that in Figure 1, the "very conservative" group stands out, while other groups are mostly similar. They suggest the interpretation of the figure reflect this and that this group be investigated further in the paper's other analyses. They write: *"Regarding Figure 1, all the action is in the most conservative tail of the distribution. This is really interesting. But the authors' interpretation of the findings departs from the data. For example, they write that "the most liberal respondents were the most healthy", but liberals had similar health to all but the most conservative. Additionally, based on the Figure 1 distribution, it seems odd to group "conservative" with "very conservative". The changes in this most conservative group are not really explored further in the paper."*

- The reviewer raises an important point: the “very conservative” group is both the most different from other ideological groups and shows the largest change over time, while the other groups show only small changes. We have edited the language on page 5 describing these findings to align with this.

- We agree that these findings are interesting, but we are limited in our ability to explore them further: the relatively small size of the “very conservative” group (especially when limiting to respondents with complete data in both waves) prevents us from breaking out these respondents from other conservatives in figure 2, which also subdivides respondents by their patterns of ideological change. We have added a figure to the appendix (section 2, figure A2) that shows biomarkers among conservative and very conservative respondents based on their patterns of change (where allowable given privacy constraints of the Add Health data); while these analyses suggest there are not hugely different patterns between conservative and very conservative respondents, the small sample sizes prevent us from drawing strong conclusions. A footnote on page 6 in the main text notes this finding.

- As the reviewer notes in another comment, the mortality analyses do not show this pattern: in most models, all ideological groups (except liberals) have significantly higher mortality than the very liberal in 2020-2022. We have therefore not chosen to highlight the distinctiveness of the very conservative in our overall characterizations of the papers’ findings in the introduction and conclusion.

The reviewer requests greater exploration of the changes in biomarkers shown in figure 2. They write: *“Figure 2 shows average health on the y axis against changes in political affiliation on the x (category) axis. Why have the authors included wave 5 health? If they are interested in how health shapes political affiliation, should they just use baseline (wave 4 health)? Also, can the authors show the opposite: change in health vs. wave 4 political affiliation? (to complement Fig 4 which focuses on mortality) I think there is more they can do to unpack (descriptively) whether the shift from wave 4 to 5 is a result of worsening health among conservatives or increased conservatism of people with poor health, or both.”*

- The revised manuscript makes an effort to clarify Figure 2, which we think does what the reviewer suggests: Figure 2 shows both wave 4 health (in white) and wave 5 health (in gray), and the panels sort respondents based on their ideology in *both* waves. Each panel includes the same set of respondents in both waves. So, the reader can see that respondents who would become more conservative (or liberal) by wave 5 were already slightly less (more) healthy than other respondents in wave 4.

- Because waves are spaced fairly far apart, we cannot precisely identify when respondents’ health declined or when their ideology changed, so we cannot say whether (for example) that the group that became conservative was less healthy before or after they became conservative—only that both happened between 2008 (when those who would become more conservative were only slightly less healthy) and 2016 (when they had become both conservative and much less healthy).

- We have revised the language around Figure 2 to clarify its features and its takeaway on page 6-7.
- We have also added a table to the appendix regressing wave 5 health outcomes on wave 4 ideology, as well as the reverse—regressing wave 5 ideology on wave 4 health outcomes—in SA 1 (Table A5). The data are consistent with the hypothesis that less healthy people in wave 4 are moving to the right over this time frame (although the relationship loses statistical significance when adding controls); wave 4 ideology does not predict change in health over this time-frame. We have added a footnote noting this on page 7 and updated the interpretation of Figure 2 to reflect these findings on page 6-7.
- Should the manuscript move forward, we are happy to work with the editor/reviewer to further update the figure or add detail if we have misunderstood the reviewer's request.

The reviewer points out an interesting pattern in the relationship between mortality and ideology as measured in different waves. They write: *"In Figure 4, the authors do not comment on the wave 5 data (red dots), but the data suggest that political ideology shifted in such a way that ATTENUATED the relationships between internal mortality and conservative politics (e.g. perhaps sick conservatives became liberal) and between external mortality and liberal politics (e.g. perhaps being exposed to opiod overdoses makes liberal people more conservative). Any potential explanation for this pattern in the data?"*

- We agree with the reviewer that this is a notable pattern. To offer a different interpretation, though, the wave 5 estimates are closer to zero for both internal and external causes of death in the initial years after the wave 5 measures were taken. Wave 4 ideology also showed weaker relationships with all three outcomes in the first years after it was taken, then grew closer to wave 3 measures over time. It could be that there is a lag between expressing an ideological position and its translation into mortality—perhaps, for example, if it takes time for ideologically-related loss of trust in medicine to manifest in receiving less care.
- Because the more recent years of the wave 5 measure overlap with the COVID-19 pandemic, we cannot say for sure whether any differences in its relationships are due to maturation of the measure or changes in the political context, so we hesitate to address this in the manuscript.
- We also take the reviewer's point that, if anything, conservative ideology in wave 5 appears to be moving towards predicting *higher* externally-caused deaths, rather than the opposite, as was the case in the past. Because these results are not significant, or significantly different from past years, though, we cannot draw strong conclusions about this. Future years of death data may shed further light upon this.

The reviewer requests a clarified label on figure 4: *"In Figure 4, the Y axis title should specify "Pr(Dead—Conservative)" or ""Pr(Dead—Conservative)" - "Pr(Dead—Liberal)"", as the current label doesn't indicate whether positive numbers reflect excess mortality for conservatives or liberals."*

- We appreciate the reviewer's suggestion and have updated the label on figure 4 to clarify this.

The reviewer, though they recognize the advantages of looking at cumulative mortality over time, argues that showing year-by-year mortality would more clearly show the timing of the emerging health gap. They write: *"...the focus on cumulative death makes it difficult for the reader to accurately pinpoint the moment when the relationship changes, as it looks like the relationship remains negative even after it has already turned positive. Specifically: whereas the design of Figure 4 suggests a focus on the statistically significant differences after 2020, if the authors plotted year-on-year (non-cumulative) death rates, it would be clear that the emergence of a conservative health disadvantage starts in 2015 (which incidentally coincides with the first Trump campaign and the nationalization of anti-institutional populism). In sum, I think it would be helpful if the authors could show annual mortality rates in addition to the cumulative."*

- We appreciate the reviewer's weighing of the merits of showing cumulative vs. year-by-year deaths. We ultimately opted to leave the cumulative analysis in the manuscript for reasons of power, but we have added figure A4 to section 4 in the appendix showing the relationship between death in any individual year and ideology. As the reviewer suspects, the year-to-year estimates are variable and not very precise, given the small numbers of death in each year. Broadly speaking, the coefficients are directionally consistent with conservatism predicting mortality begin to appear in 2016, but this is not consistent. We have added a footnote on page 9 referring to these findings.
- We have also added analyses to the appendix regressing mortality during several three-year periods on ideology and other variables, paralleling the 2020-22 analysis. This is included in SA 6. These analyses suggest the 2020-22 period is substantively different from 2017-2019 and, for wave 4 measures, 2014-16: the gaps in health present in 2020-2022 were not yet present in earlier periods.
- Based on this evidence, we have maintained our claim in the paper that the gap in mortality emerged only after 2020. One potential explanation for this timing is that the gaps in biomarkers observed in 2016-2018 were more immediate consequences of gaps in engagement with the medical system, while gaps in mortality took longer to manifest.

The reviewer points out it is not clear there was a qualitative change in the relationship between ideology and health after the COVID-19 pandemic rather than a continuation of preexisting trends. They write: *"...I worry that the focus on the pandemic era distorts the reader's attention from the root causes of the trends the authors are illuminating. E.g., the authors write (p10, bottom): "there is reason to believe that at least the period since 2020 has changed the relationship between politics and health in the population at large." They also write (top of p16) "the initial decline in conservatives' health... reflected a reshuffling of less healthy people between political coalitions". But if my reading of their data is correct, these statements are not accurate. Based on Figure 4, it looks like the relationship between politics and health changed prior to COVID (starting around 2015 with the first Trump presidency)*

and the pandemic illustrated the deadly consequences of this change."

- We thank the reviewer for raising this important point about the interpretation of our results. We have edited the manuscript in several places to bring our statements of the findings in closer line with the evidence. In the abstract and the discussion (page 19, formerly page 16 as noted by the reviewer), we have removed references to the timing of the sorting and trust mechanisms. And on page 11 (formerly 10), we have removed the reference to the notion that the pandemic changed the relationship between ideology and health.

- There is some evidence from other work on the timing of the political gap in trust: we have added citations to other work showing the gap in trust in medicine as an institution between left and right did not emerge until after the pandemic (page 15). However, we also refer there to work suggested by Reviewer 2 that shows some of the divides in health attitudes (e.g., flu vaccines) predate the pandemic. So, while the scope of the political divide has expanded since the pandemic, we cannot rule out that it played a role in the earlier gaps in health.

- The manuscript emphasizes, in its discussion of Figure 3, that the available evidence suggests that the health divide that emerged in the 2010s was at least partially a result of the changing demographics of who called themselves liberal and conservative, but around half the gap remains unexplained (page 9). This could be the result of gaps in engagement with the healthcare system that predated the pandemic, but as we note on page 16, we lack the data to test this directly.

- As we noted in our response to the previous question, the additional analyses breaking down mortality by year or three-year period suggest the political gap in mortality in fact did not emerge until after 2020, or at least was not at all consistent. As the reviewer points out, though, we cannot say whether the relationship between ideology and death would have grown even absent the pandemic's effects on trust in medicine. Our adjusted statements of our findings no longer claim that this mortality gap is due to that mechanism.

- We have also clarified in our discussion of our original survey that we cannot speak to the origins or timing of the gaps in the Add Health data, since the survey took place after it concluded–see page 16 and page 19.

Recalling the distinctiveness of "very conservative" respondents in figure 1, the reviewer asks about the relationship between individual ideology categories and health over time for the outcome of mortality: *"Table 1 – in contrast to Figure 1 – shows a more or less continuous gradient between political ideology and mortality risk during COVID. This difference from Figure 1 is interesting but is hardly remarked on. As a companion to Figure 4, it would be very compelling to show 5-level individual-level ideology-mortality risk gradients for different time periods – not just 2020-2022 – and to present these as a figure (perhaps adjusted for age and sex)."*

- This is an important distinction from the findings in figure 1, and we have added a sentence on page 12 remarking on this.
- As noted above, we have added tables to the appendix regressing mortality in three-year intervals on ideological categories, with and without controls (see SA section 6, table A9 and A10). As we also note above, though, mortality in periods before 2020-2022 had no relationship with any category of ideology. We have therefore opted not to highlight these results further with a figure.

The reviewer asks about alternative specifications of age in the regression models: *"In Table 1, birth year (age) is included as a continuous variable. Is it appropriate to include age as a simple linear term? Are the findings robust to other specifications?"*

- To address this question, SA 7 table A11 replicates what was Table 1 (now Table 2 in revised manuscript), but includes age as a dummy variable based on three-year birth periods (the respondents are born between 1974 and 1983) for maximum flexibility in the relationship between age and health. Results are robust to this specification.

The reviewer suggests we do more to emphasize the motivation and findings of the models with county fixed effects. They write: *"On p13, the authors should make more of a meal about controlling for county FE. Most of the compelling prior results are at the county level, so these results are based on completely different source of variation in the data. This could be highlighted for the reader."*

- We appreciate the reviewer prompting us to highlight these findings. We have added text to page 15 to motivate and emphasize the county fixed effects as the reviewer suggests.

The reviewer requests more explanation of the original survey in the main text, writing: *"The original survey needs a couple sentences of explanation in the main text (p13), to underscore that it was a large online sample w/sample size X and post-stratification weights to match national demographics. (Or some such.) Details can be provided in the methods and materials, but the reader needs some orientation."*

- We have added a footnote to page 16 providing a brief description of the survey sample and referring the reader to the relevant section of the Methods. We are mindful of the word limit, but we can add more details if the reviewer feels they are needed to orient the reader.

The reviewer requests a more detailed label/caption on figure 5, writing: *"Figure 5 – the legend was rather confusing as the abbreviations are not defined. The figure needs a detailed explanatory note that explains each of the series in the legend."*

- Figure 5 now includes an expanded legend that better explains each of the series. We also include phrasing in the main text that more clearly describes the different estimates, and the caption more clearly defines the labels on the y-axis.

The reviewer suggests more be done to unpack the finding that ideology is a weaker predictor of health attitudes and behaviors than other political variables. They write: *"In Figure 5, it is a bit inconvenient that the political ideology results are much less clean than the voting intention results. It is hard to know what to make of this. The authors emphasize the voting intention results (Trump v Biden), but the rest of the paper focuses on ideology. While these data help advance a hypothesis about loss of trust, in some ways these data open new challenges, making it harder to interpret what comes beforehand. It would be helpful to see (in the Appendix) some data on the correlation between voting intentions, partisanship, and ideology, how these concepts are distinct, and some reflection on what exactly "ideology" is measuring in the main paper results."*

- First, to address this empirically, the SA section 10 now includes cross-tabs of partisanship by ideology and vote choice by ideology. Most liberals consider themselves Democrats (about 83%) in these data and very few consider themselves Republicans (less than 5%). The tightening relationship between ideology, partisanship and vote choice in recent decades has been well documented (for example, see Grossman 2024).

  It is unclear why the relationship between trust and political attitudes is weaker for ideology than for vote choice or partisanship. One possibility is that when asked about their ideology, more people say they are moderate (i.e. do not choose liberal or conservative) when compared to people who say they are not attached to a party (i.e. do not choose Democrat or Republican). In our survey data, 20% of respondents call themselves Independent, but 39% call themselves moderate (tables A24 and A25). But most of these moderates have political preferences: 65% of moderates identify as Republicans or Democrats, and 64% indicated support for Trump or Biden (tables A26 and A27). The "moderate" ideological category may therefore conceal some variation in political attitudes that partisanship measures or vote choice can better capture.

- To explore these points, SA table A28 includes attitudes by vote choice, just among ideological moderates. Among moderates, vote choice is quite predictive of trust and engagement with the healthcare system. We note this in a footnote on page 17.

- Another possible explanation, and related to the point above, is that much of this division in trust is driven not by ideology in itself, but by elite cues around the response to COVID-19; Presidents Trump and Biden sent widely varying signals about the trustworthiness of public health measures during the pandemic. Vote choice between these two candidates (and its close cousin, partisanship) would then be a more direct measure of what's driving the change in trust. Ideology, though an increasingly good predictor of vote choice, would be an indirect proxy for the real causal mover. If this is the case, health outcomes may be even more polarized by vote choice or partisanship than by ideology—though we can only measure the latter here. We allude to this in the discussion on page 19.

The reviewer suggests the summary of the survey findings be more mindful of its limitations. They write: *"it is hard to evaluate the significance of these findings as an explanation of their primary results, as these findings are not presented alongside alternate hypotheses and the data come from a period several years after the main change that they study."*

- We appreciate this caution from the reviewer and have added a caveat on page 16 when introducing the survey and on page 19 when discussing the paper's limitations. We now note that because it took place in 2024, this evidence cannot speak directly to attitudes in earlier years, and we clarify its role as proposing and exploring a possible explanation.

Continuing from the above point, the reviewer asks whether more can be done to assess the role of trust in the health gap. They write: *"Can the authors do more to test whether their "trust" hypothesis is a plausible explanation for the observed trends? Could they assess whether trust has been a predictor of care utilization and health outcomes over time? Could they assess whether trust has diverged for liberals and conservatives and when this happened? In particular, what happened around 2015 that initiated this pattern?"*

- The revised manuscript cites prior work that gives at least a partial answer to the reviewer's question: a partisan gap in trust in medicine as an institution did not exist before 2020, but has since emerged (e.g., Blendon and Benson 2021, Brady and Kent 2022). There is unfortunately no comparable time-series data measuring trust in one's personal doctor, adherence to their advice, or belief in medicines. There is some evidence that liberals and conservatives exhibit some slightly different health behaviors (e.g., flu vaccine uptake) before the pandemic, which we now cite as well. We add a sentence on page 15 detailing these trends.

  The revised manuscript also includes a short paragraph in the conclusion on page 19 noting that the timing of our survey and the lack of trust measures on the Add Health survey prevent us from directly testing the timing and predictiveness of the trust gap and how it relates to health outcomes. We identify this as an important area for future work.

- In an effort to address this question, we looked further for data on health care utilization over time by political affiliation. We identified three surveys spanning the 2010s that asked slight variations on whether the respondent had "Gone to a health care provider for a general physical exam."[1] These surveys echo a finding we present (in Figure 5) regarding whether the respondent had seen their primary care provider in the last 12 months.

  In 2012 and 2016, Democrats and Republicans reported seeing their physician for an annual check-up at similar rates. In 2023, Republicans are 8.03 pp. less likely to report having had an annual physical screening ($\beta = -.0803$ two-tailed t-test; $p = .032$; CI: -.15,-.07). We present this graphic below. A similar pattern persists after controlling for available demographics (in 2023, with demographic controls: $\beta = -.0799$ two-tailed t-test; $p = .044$; CI: -.16,-.002).

  The 2023 Axios survey also asks respondents, "How much trust do you have, if any, in information about health topics from the following? ... Your personal doctor." We

[1] Pew asked this in 2016. RWJ asked in 2012 "In the past 12 months, have you seen a doctor or nurse practitioner for a routine check-up, or not?" And Axios in 2023 asked, "In the last year, have you had an annual health screening with a physician."

Figure 1: Had a physical/health screening in last 12 months

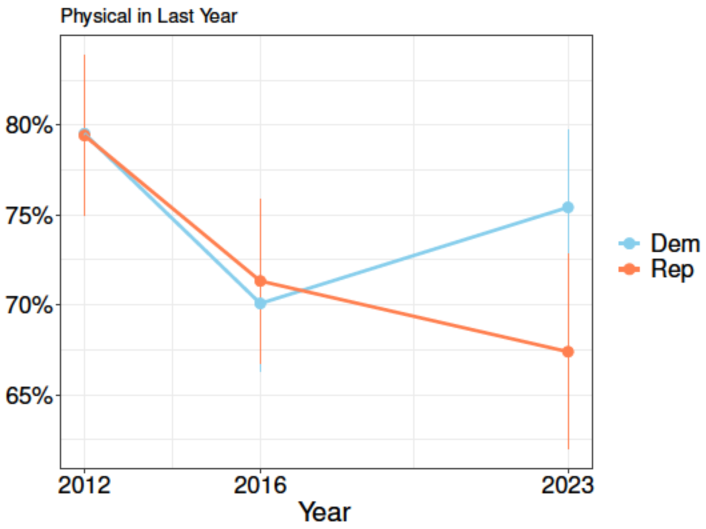

cross-tab the relationship between people's trust in information from their personal doctor with whether they reported having seen a doctor for an annual physical screening. A clear gradient emerges: 80% of respondents who report having "a great deal of trust" report having had an annual physical exam in the prior year compared to just 27% of respondents who say they have "None at all."

Table 1: Health screening in last 12 months by Trust Info From Personal Doctor

	None at all	Not very much	A fair amount	A great deal	Total
No	73.04	45.62	36.94	19.56	32.01
Yes	26.96	54.38	63.06	80.44	67.99
Total	100.00	100.00	100.00	100.00	100.00

We find these data interesting and supportive of the reviewer's notion that something changed between 2015 and 2024. However, they do not allow us to date the timing of the change more precisely, and we are hesitant to include another data source in the paper due to space limitations. We have therefore not included these data in the revised manuscript, instead relying on the added citations for the notion that a gap in engagement grew after COVID-19. However, if this is a sticking point for the manuscript moving forward, we are happy to work with the reviewer and editors to include it in the main text or appendix.

The reviewer asks for greater discussion of educational realignment and its relationship with health. They write: *"Can the authors comment more on the shift of less educated people to the conservative coalition. Is this a different causal pathway and potential confounder to control for? Is it constitutive of the same causal pathway? Just thinking out loud, but perhaps there is an interaction between education and politics. Perhaps it's not [just] that less educated*

people with worse health outcomes shift to Republicans. It's that there's an interaction which makes the education gradient much steeper. Liberals favor public health paternalism (and redistributive policies), which protects less educated people from poor health decisions and from structural inequities. Conservatives favor more libertine policies, which leave less educated people to suffer the consequences of their choices and of local policy regimes."

- We view the educational realignment as part of the causal pathway we explore in the decomposition analysis (figure 3): as liberals have become more educated and the income gap has eroded or reversed, the liberal coalition has become healthier. We have added a sentence to page 8 explicitly stating this as in-line with our proposed explanation.

- The reviewer also raises an interesting question about the interaction between individual partisanship and the aggregate effects of partisan control on health policy. We hope to explore this in future work, but we feel it is outside the scope of this manuscript.

Responses to Reviewer 2

The reviewer suggests we incorporate a discussion of the finding that liberals may have become more pro-medicine or pro-science as conservatives have become less trusting in these institutions. They write: *I think they might enhance their discussion with some of the literature that suggests that liberals may have also changed their health views (to be more pro-science).* They suggest a citation on this point. They also suggest a citation to earlier work showing differences in trust in medicine by party.

- We thank the reviewer for suggesting these additional references. We have added these citations both to the introduction on page 3, as support for the notion that right-leaning Americans are less engaged with the healthcare system. We have also added a sentence to page 15 noting that, as the reviewer points out, conservatives are not the only group changing their attitudes: liberals have become *more* trusting of scientific experts (also referring to their suggested citation). We appreciate these prompts to engage with these pieces.

Responses to Reviewer 1

The reviewer writes that the revision was *highly responsive to my original critiques*, and they *continue to believe that this paper makes an important and novel contribution to the literature on political ideology as a social determinant of health.* We appreciate the reviewer's favorable appraisal of the revision and the paper overall.

The reviewer asks that the paper *specify "which time period" and consider a relative measure of association given the low background death rates* on page 3 when summarizing findings on conservatives' higher death rates.

- We thank the reviewer for pointing out that this sentence could be difficult to interpret. We have replaced it with the following text:
- *while only 0.2 percent of "very liberal" respondents died of internal causes between 2020 and 2022, the chance for people who identified as "very conservative" was 1.14 percentage points higher*
- The new phrasing highlights the low background death rates among liberal respondents. It also makes clearer that the comparison is between the most liberal and most conservative respondents in the years 2020-2022, rather than comparing across time periods. We hope this addresses the reviewer's concern.

The reviewer recommends the year-by-year mortality figure in the appendix replace the cumulative mortality figure in the main text. They write that the cumulative figure is *misleading, as it focuses attention on later years (COVID) rather than earlier when the emergence of the conservative disadvantage occurs.* They point out that the year-by-year figure directly shows the timing of the gap in mortality, rather than the cumulative figure, which allows only *roundabout* statements of the timing and places undeserved emphasis on the years after 2020 *only because they are making inferences on cumulative mortality, and by 2020 enough years of have passed for excess mortality to accumulate to levels of "significance".* They further argue that the cumulative plot *do[es] not correspond to how most readers will interpret that figure, because each annual data point includes all prior years' data.*

- We agree with the reviewer that establishing the timing of the emergence of the mortality gap is important to the paper's contribution. We also appreciate the reviewer pointing out that the cumulative figure may confuse readers by including past deaths in each year's estimate. We therefore decided to replace the cumulative figure with a non-cumulative one.
- While we agree with the reviewer that year-by-year estimates present the most granular picture of the mortality gap over time, we are also hesitant to present results that rely on very few deaths in any given year, given the age of this population.
- To strengthen our statistical power beyond the year-by-year plot while avoiding the features of the cumulative plot that the reviewer found misleading, we have replaced

the mortality figure in the main text with a version that presents coefficients for deaths occurring in 3-year bins. The new figure shows the relationship between ideology and mortality separately for deaths occurring in (e.g.) 2020-22, 2017-19, and so on. The year-by-year and original cumulative plots are in the appendix.

- As the reader can now see, only deaths that occurred between 2020 and 2022 showed a significant relationship with ideology, though internally-caused deaths between 2017 and 2019 were significantly predicted by wave 4 ideology. Because the other years' coefficients do not statistically differ from one another, we unfortunately cannot be any more precise about the relative size of these gaps over time.

- While we agree with the reviewer that there are suggestive signs in the year-by-year plot that the mortality gap began earlier, the relative imprecision and variability of those estimates prevent us from making any further statistically-supported claims about the timing of the gap beyond what the three-year bins allow.

- We hope this updated figure addresses the reviewer's concerns about clarity and interpretation.

The reviewer *appreciated the additional regression analyses, looking at 3-year periods, in the appendix*. However, they note a discrepancy between the plotted coefficients for 2017-2019 and the coefficient on that group of years in the binned mortality table.

- We appreciate the reviewer bringing this discrepancy to our attention. It was caused by a mistake in which the 2020-2022 mortality measure had been rescaled to range from 0-100, while earlier years were scaled to range from 0-1. All mortality analyses have now been updated to range from 0-100 (so that coefficient can be interpreted as a percent out of 100 and alleviates decimals with leading 0s). We regret the error and are grateful for the reviewer's careful eye. These tables – death in three-year windows – have been replaced by Figure 4 in the main-text and SA 5 which measure deaths over three-year time-spans.

The reviewer found Figure 5 *quite confusing*, noting that , *it took me quite a while to make sense out of the legend and understand what the different data series were.* They recommend *to drop the first two columns and just show ideology, for a cleaner comparison with the rest of the paper.* They further recommend *showing just ONE data point for each outcome (probably the grey multivariate dots) and drop the rest. The bivariate relationships are not particularly interesting and the results for respondents with chronic diseases are pretty similar to the general population* in order to highlight the main result.

- We appreciate the reviewer pushing us to clarify this figure further. We have followed the reviewer's suggestion to show only one data point for each outcome (the multivariate models, as the reviewer recommended) and have moved the remaining models to the appendix; we agree with the reviewer that the additional models do not add key information to the main result.

- However, we ultimately decided to leave all three measures of political leanings in the figure (ideology, partisanship, and vote choice). Many readers of the manuscript, including the reviewer in the first round of review, have expressed interest in differences between these measures in their relationship with health outcomes, and many have found the absence of political measures beyond ideology in the Add Health data to be an important limitation. For these reasons, we feel it is important to show that at least the relationship between politics and health-related attitudes and behaviors is consistent across multiple measures of political leanings.
- We hope that limiting the figure to show only a single estimate per predictor-outcome pair, along with the expanded caption added in the first revision, has made the figure sufficiently readable. We have also rearranged the figure so that ideology is the first predictor shown when reading the figure left-to-right, so that its flow with the earlier results in the manuscript is smoother.

The reviewer expresses that they *love the new public opinion data added to the response letter on historical patterns in health screenings and trust*, and they suggest we add it to the appendix, *if only to corroborate your results in national samples.*

- We are glad the reviewer found this data helpful, and we have added it to appendix section 10. We refer the reader to the section in a footnote on page 15.
